# A new genus and eight newly recorded genera of Braconinae Nees (Hymenoptera, Braconidae) from China, with descriptions of fourteen new species

**DOI:** 10.3897/zookeys.1038.55258

**Published:** 2021-05-19

**Authors:** Yang Li, Cornelis van Achterberg, Xue-xin Chen

**Affiliations:** 1 State Key Lab of Rice Biology, Zhejiang University, Hangzhou 310058, China Zhejiang University Hangzhou China; 2 Ministry of Agriculture Key Lab of Molecular Biology of Crop Pathogens and Insects, Zhejiang University, Hangzhou 310058, China Zhejiang University Hangzhou China; 3 Zhejiang Provincial Key Laboratory of Biology of Crop Pathogens and Insects, Zhejiang University, Hangzhou 310058, China Zhejiang University Hangzhou China; 4 Institute of Insect Sciences, College of Agriculture and Biotechnology, Zhejiang University, Hangzhou 310058, China Zhejiang University Hangzhou China

**Keywords:** Bathyaulacini, Braconini, Euurobraconini, new combination, new record

## Abstract

A new genus, *Parallobracon***gen. nov.**, of the subfamily Braconinae (Hymenoptera, Braconidae) is described to include *Parallobraconprolatus***sp. nov.** Eight genera *Chaoilta* Cameron, *Cyanopterus* Haliday, *Gammabracon* Quicke, *Ischnobracon* Baltazar, *Monilobracon* Quicke, *Pseudospinaria* Enderlein, *Vipiomorpha* Tobias, and *Zaglyptogastra* Ashmead (Hymenoptera: Braconidae: Braconinae) are newly recorded from China, their 21 species are revised, and 13 new species (*Chaoiltabreviceps***sp. nov.**, Cyanopterus (Ipobracon) lucidus**sp. nov.**, Cyanopterus (Ipobracon) transversus**sp. nov.**, *Gammabraconuniformis***sp. nov.**, *Gammabraconwangi***sp. nov.**, *Ischnobraconguttatus***sp. nov.**, *Monilobraconlongitudinalis***sp. nov.**, *Monilobraconmarginatus***sp. nov.**, *Parallobraconprolatus***sp. nov.**, *Vipiomorphasulcata***sp. nov.**, *Vipiomorphayunnanensis***sp. nov.**, *Zaglyptogastraexilis***sp. nov.**, and *Zaglyptogastratricolor***sp. nov.**) are described and illustrated. BracomorphaPapp, 1971, is included assubgenusinCyanopterus Haliday, 1835 (**syn. nov.**) and *Cyanopterusninghais* Wang, Chen, Wu et He, 2009, is a new combination. Keys to the Chinese species of the genera *Chaoilta*, *Cyanopterus*, *Gammabracon*, *Ischnobracon*, *Monilobracon*, *Vipiomorpha*, and *Zaglyptogastra* are provided.

## Introduction

Bathyaulacini Quicke (Hymenoptera: Braconidae: Braconinae) is a small tribe which includes three genera (*Annectobracon* Chishti & Quicke, 1995, *Bathyaulax* Szépligeti, 1906 and *Ischnobracon* Baltazar, 1963). To date, only one genus (*Annectobracon* Chishti & Quicke, 1995) was reported from China (Chishti and Quicke 1995), but in this paper, we report one newly recorded genus: *Ischnobracon*, which is a small genus with eleven described species, mainly occurring in the Oriental region (Yu et al. 2016). The biology of this genus is still unknown.

*Zaglyptogastra* Ashmead, 1900, is a relatively large genus in the tribe Euurobraconini Ashmead (Hymenoptera: Braconidae: Braconinae) with 40 described species, mainly distributed in the Afrotropical region (Yu et al. 2016). Euurobraconini is a small tribe with three genera (*Euurobracon* Ashmead, 1900, *Pseudodicrogenium* Fahringer, 1936, and *Zaglyptogastra*), and so far, only one genus (*Euurobracon*) was reported from China (Yu et al. 2016). The biology is known of only one species: *Z.cristata* (Szépligeti) has been reared from *Tryphochariaprinceps* (Blackburn) (Coleoptera: Cerambycidae) (Quicke 1983).

*Chaoilta* Cameron, 1899, and *Cyanopterus* Haliday, 1835, are two relatively large genera in the tribe Braconini Nees (Hymenoptera: Braconidae: Braconinae) with 42 and 139 described species worldwide, respectively (Yu et al. 2016). *Chaoilta* occurs mainly in the Oriental and Australasian regions, and *Cyanopterus* mainly in the Afrotropical, Palaearctic and Neotropical regions (Yu et al. 2016). Most species of *Cyanopterus* are ectoparasitoids of coleopteran larvae (including species of Cerambycidae and Curculionidae) (Yu et al. 2016); the biology of *Chaoilta* is still unknown. In this paper, two *Chaoilta* species are found in China, of which one species is new to science (*Chaoiltabreviceps* sp. nov.), and five *Cyanopterus* species are found in China, of which three species are new to science (Cyanopterus (Ipobracon) lucidus sp. nov., Cyanopterus (Ipobracon) prolatus sp. nov. and Cyanopterus (Ipobracon) transversus sp. nov.). A new genus, *Parallobracon* gen. nov., in this tribe is found and described here with the type species, *Parallobraconprolatus* sp. nov. The biology of the new genus is unknown.

*Pseudospinaria* Enderlein, 1905, and *Vipiomorpha* Tobias, 1962 are two small genera in the tribe Braconini with only two and three described species, respectively (Yu et al. 2016). *Pseudospinaria* is endemic to the Oriental region, and *Vipiomorpha* is known from the Afrotropical and Palaearctic regions (Yu et al. 2016). *Vipiomorpha* Tobias is reported from the Oriental region for first time. The biology of both genera is still unknown.

*Gammabracon* Quicke, 1984, and *Monilobracon* Quicke, 1984, are two genera that are not assigned to any tribe within the Braconinae so far. Both are small genera, with five and six described species, respectively (Yu et al. 2016). *Gammabracon* is endemic to the Oriental region, and *Monilobracon* occurs in the Afrotropical, Australasian, Oriental and Palaearctic regions (Yu et al. 2016). The biology of both genera is still unknown. In addition to the description of new genera and species, keys to Chinese species of seven genera (*Chaoilta*, *Cyanopterus*, *Gammabracon*, *Ischnobracon*, *Monilobracon*, *Vipiomorpha*, and *Zaglyptogastra*) are provided.

In this paper one new genus, *Parallobracon* gen. nov., including one new species, *Parallobraconprolatus* sp. nov. is described. Eight genera *Chaoilta* Cameron, *Cyanopterus* Haliday, *Gammabracon* Quicke, *Ischnobracon* Baltazar, *Monilobracon* Quicke, *Pseudospinaria* Enderlein, *Vipiomorpha* Tobias and *Zaglyptogastra* Ashmead are newly recorded from China, and 13 new species (*Chaoiltabreviceps* sp. nov., Cyanopterus (Ipobracon) lucidus sp. nov., Cyanopterus (Ipobracon) transversus sp. nov., *Gammabraconuniformis* sp. nov., *Gammabraconwangi* sp. nov., *Ischnobraconguttatus* sp. nov., *Monilobraconlongitudinalis* sp. nov., *Monilobraconmarginatus* sp. nov., *Parallobraconprolatus* sp. nov., *Vipiomorphasulcata* sp. nov., *Vipiomorphayunnanensis* sp. nov., *Zaglyptogastraexilis* sp. nov. and *Zaglyptogastratricolor* sp. nov.) are described.

## Materials and methods

For the identification of the subfamily Braconinae, see van Achterberg (1990, 1993) and Chen and van Achterberg (2019), for the terminology and measurements used in this paper, see van Achterberg (1988, 1993), and for additional references, see Yu et al. (2016). The medial length of the third metasomal tergite is measured from the posterior border of the second suture to the posterior margin of the tergite (T).

Photographs were made with a Keyence VHX-2000 digital microscope and the photos were slightly processed (mainly cropped and the background modified) in Photoshop CS6. For the descriptions and measurements, a Leica M125 stereomicroscope was used.

The specimens are deposited in the Institute of Zoology, Chinese Academy of Sciences, Beijing (**IZCAS**), Shanghai Entomological Museum, Chinese Academy of Sciences, Shanghai (**SHEM**) and in Institute of Insect Sciences, Zhejiang University, Hangzhou (**ZJUH**).

## Taxonomic accounts and keys

### Genus Chaoilta

Taxon classificationAnimaliaHymenopteraBraconidae

Cameron, 1899

D1DE99F3-E0B6-5359-8F68-2B8362A9D0A9

**Figure 1.**
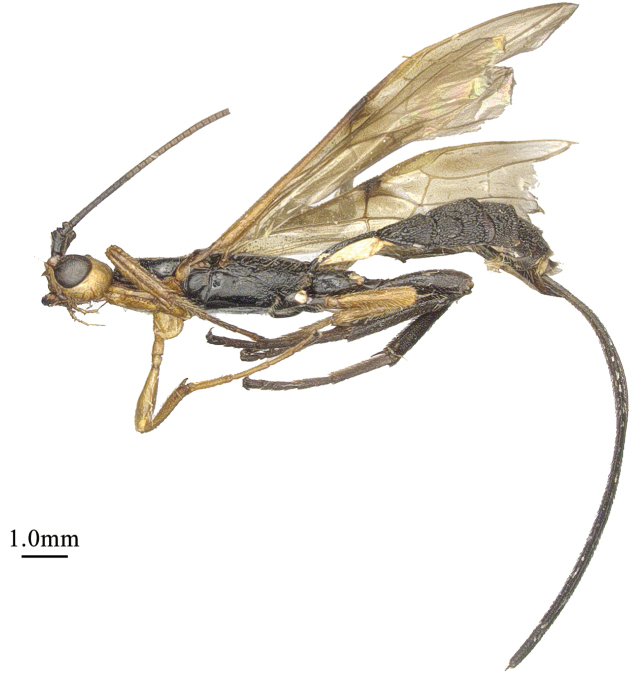
*Chaoiltabreviceps* sp. nov., ♀, holotype, habitus, lateral view.

**Figure 2.**
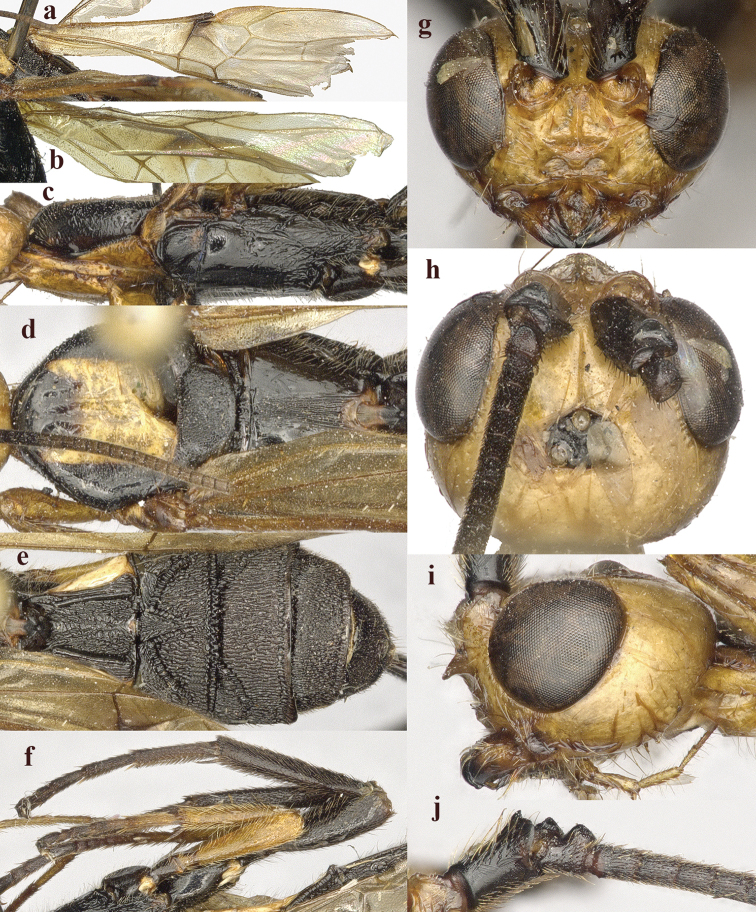
*Chaoiltabreviceps* sp. nov., ♀, holotype. **a** fore wing **b** hind wing **c** mesosoma, lateral view **d** mesosoma, dorsal view **e** metasoma, dorsal view **f** hind leg, lateral view **g** head, anterior view **h** head, dorsal view **i** head, lateral view **j** scapus outer side, lateral view.

**Figure 3.**
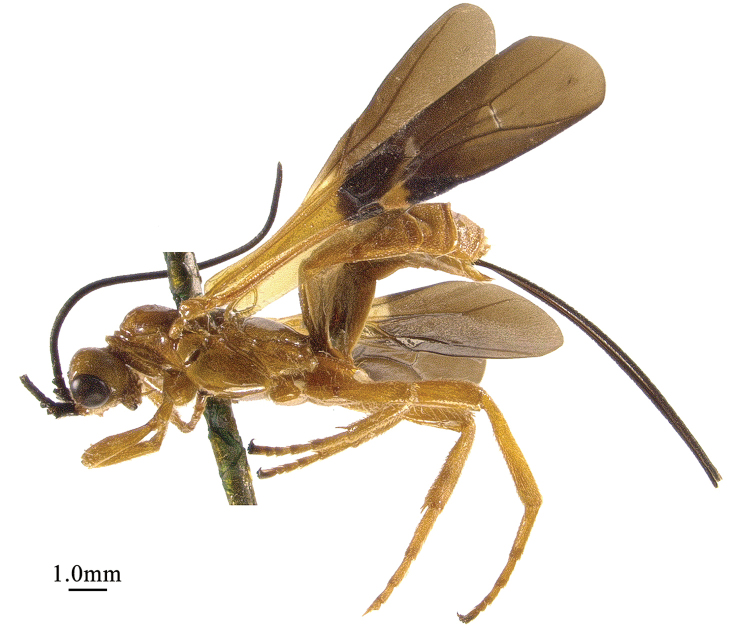
*Chaoiltahimalayensis* (Cameron, 1899), ♀, habitus, lateral view.

**Figure 4.**
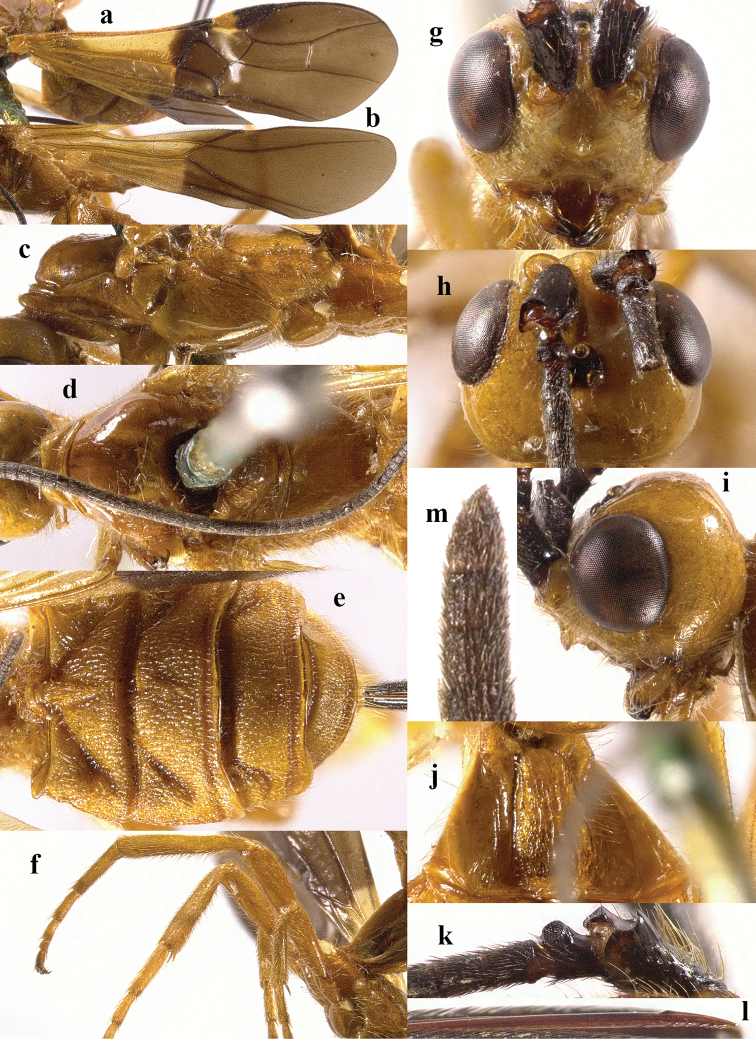
*Chaoiltahimalayensis* (Cameron, 1899). ♀ **a** fore wing **b** hind wing **c** mesosoma, lateral view **d** mesosoma, dorsal view **e** metasoma, dorsal view **f** hind leg, lateral view **g** head, anterior view **h** head, dorsal view **i** head, lateral view **j** first metasomal tergite, dorsal view **k** scapus outer side, lateral view **l** apex of ovipositor, lateral view **m** apex of antenna.

Figures 1 , 2 , 3 , 4

Chaoilta Cameron, 1899: 80; Szépligeti 1904: 17; Baltazar 1962: 749; Quicke 1981: 76, 1987: 106, 1991: 76. Type species: Chaoiltalammellata Cameron, 1899 (Monobasic).
Platybracon Szépligeti, 1900: 49. Type species: Platybracondepressus Szépligeti, 1900 (Monobasic). Synonymised by Roman 1913: 48.
Blastomorpha Szépligeti, 1900: 50. Type species: Blastomorphadecorata Szépligeti, 1900 (Designated by Viereck, 1914: 22). Synonymised by Cameron 1903: 121.
Iphioilta Ramakrishna Ayyar, 1928: 60. Type species: Iphioiltamalabarica Ramakrishna Ayyar, 1928 (Original designation). Synonymised by Quicke 1987: 106.

#### Diagnosis.

Body medium-sized to large; terminal antennomere slightly acute apically; median antennomeres often longer than wide, rarely slightly wider than long; pedicellus petiolate; scapus highly modified, lower inner apical rim of inner side of scapus interrupted because of a second small, more apical area; eye glabrous, weakly emarginated; face with a more or less strongly produced medially, transverse ledge, sometimes with a medial horn-like and distally concave projection above this; clypeus moderately narrow and without dorsal carina; malar suture moderately developed; labio-maxillary complex normal, not elongate; frons broadly impressed, with some setae and a median groove; mesosoma largely smooth and shiny, often more or less strongly dorsoventrally compressed; notauli largely absent; scutellar sulcus narrow and crenulate; propodeum flattened; angle between veins 1-SR and C+SC+R of fore wing more than 75°; vein r-m of forewing usually with two bullae; apex of hind wing vein C+SC+R with one thickened bristle; base of hind wing with glabrous area distal to vein cu-a; claws simple; fore tarsus at least 1.6× longer than fore femur (excluding trochantellus); fourth tarsal segment with numerous long bristles apico-ventrally, almost reaching apex of telotarsus; hind femur and tibia with relatively dense and long setae ventrally; lateral areas of T I wide and completely flattened; T II with deep oblique lateral grooves connected to wide sublateral grooves; antero-lateral grooves of T III short, and medial part of tergite 1.5× wider than its lateral parts; T III–V with distinct oblique antero-lateral grooves; ovipositor with dorsal nodus and ventral serrations subapically.

#### Biology.

Unknown.

#### Distribution.

Afrotropical; Australasian; Oriental.

#### Note.

This genus is newly recorded from China.

### Key to Chinese species of the genus *Chaoilta* Cameron

**Table:** 

1	Metasomal tergites yellow (Fig. 4e); mesoscutum brownish yellow anteriorly and laterally (Fig. 4d); basal half of fore and hind wings membrane yellow and apical half dark brown (Fig. 4a, b), with a pale streak from base of pterostigma to vein 2-SR+M (Fig. 4a); apical 2/3 of pterostigma blackish brown (Fig. 4a); propodeum largely glabrous, with weak longitudinal striae medio-posteriorly (Fig. 4d); [China; India]	***C.himalayensis***
–	Metasomal tergites black (Fig. 2e); mesoscutum black anteriorly and laterally (Fig. 2d); fore and hind wings membrane slightly infuscate (Fig. 2a, b), and with a darker area below parastigma (Fig. 2a); pterostigma entirely pale yellow (Fig. 2a); propodeum with setae and punctures laterally, and with strong longitudinal striae medially (Fig. 2d); [China]	***C.breviceps* sp. nov.**

### Chaoilta breviceps sp. nov.

Taxon classificationAnimaliaHymenopteraBraconidae

75224D53-5F33-535A-8241-FD9B984B8D68

http://zoobank.org/3C5AD8DD-C704-42A7-BFE0-67DF18E7BE90

Figures 1 , 2

#### Material examined.

***Holotype***: ♀, China, Yunnan Prov., Xishuangbanna Meng’a, 1000 m, 23.V.1958, Pu Fuji, No. IOZ(E)1964614 (IZCAS). Paratype. 1♀, China, Yunnan Prov., Xiaomengyang, 810 m, 30.III.1957, Zang Lingchao, No. IOZ(E)1964548 (IZCAS).

#### Diagnosis.

This new species is very similar to *C.lammellata* Cameron, 1899 [India], but can be separated from the latter by the following characters: mesoscutum yellow, middle lobe anteriorly and lateral lobes laterally black (entirely yellow in *C.lammellata*); ovipositor sheath 0.86–0.88 × as long as body (1.35 × in *C.lammellata*); scape entirely blackish brown (reddish brown basally and its apical half black in *C.lammellata*); propodeum with strong longitudinal striae medially, and with punctures laterally (smooth in *C.lammellata*); T V weakly sculptured (smooth in *C.lammellata*).

#### Description.

Holotype, ♀, length of body 11.1 mm, of fore wing 9.8 mm, of ovipositor sheath 9.6 mm.

***Head*.** Antenna incomplete, with 26 antennomeres remaining; median antennomeres 1.1× wider than long; third antennomere 1.1 and 1.2× longer than fourth and fifth, respectively, the latter 1.3× longer than wide; length of maxillary palp 1.1× height of head; malar suture with short setae, and with fine sculpture (Fig. 2i); clypeus height: inter-tentorial distance: tentorio-ocular distance = 3: 10: 8; clypeus with sparse long setae; eye weakly emarginated (Fig. 2g); face with a moderately developed transverse protrusion, dorsal side of protrusion with a medio-longitudinal carina which with a few branches (Fig. 2g, i); eye height: shortest distance between eyes: head width = 17: 21: 43; frons smooth, strongly and broadly depressed behind antennal sockets, with some short setae, sparsely and weakly punctate laterally, and with a strong median groove (Fig. 2h); vertex with some fine punctures, and largely glabrous except for a few short setae; minimum distance between posterior ocelli: minimum diameter of elliptical posterior ocellus: minimum distance between posterior ocellus and eye = 5: 6: 14; length of malar space 0.6× basal width of mandible; in dorsal view length of eye 1.3× temple (Fig. 2h).

***Mesosoma*.** Length of mesosoma 3.4× its height (Fig. 2c); pronotum emarginated medio-apically, and with dense setae postero-dorsally; notauli largely absent (Fig. 2d); middle lobe of mesoscutum weakly convex anteriorly, mesoscutum largely glabrous, but with long setae along imaginary notaulic courses (Fig. 2d); scutellar sulcus deep and narrow, crenulate (Fig. 2d); scutellum sculptured, more or less flattened; metanotum flattened medially (Fig. 2d); propodeum largely smooth, but with some longitudinal striae medially, and with a few weak punctures and long setae laterally (Fig. 2d).

***Wings*.** Fore wing (Fig. 2a): SR1: 3-SR: r = 27: 17: 3; 1-SR+M bent after arising from 1-M, 1.7× longer than 1-M; 2-SR: 3-SR: r-m = 9: 17: 6; cu-a interstitial. Hind wing (Fig. 2b): SC+R1 1.5× longer than 1r-m.

***Legs*.** Length of fore femur: tibia: tarsus = 24: 27: 53; length of hind femur: tibia: basitarsus = 37: 60: 25; length of femur, tibia and basitarsus of hind leg 3.1, 7.5 and 5.2× their maximum width, respectively (Fig. 2f).

***Metasoma*.** Length of T I 1.3× its apical width, median area convex and coarsely sculptured (Fig. 2e); lateral grooves of T I completely smooth (Fig. 2e); T II largely coarsely sculptured (Fig. 2e); apical width of T II 2.8× its median length, triangular medio-basal area of T II medium-sized, attached to short medio-longitudinal carina, but absent near posterior margin of T II, grooves besides medio-basal area strongly crenulate; antero-lateral areas of T II coarse, anterior grooves wide and distinctly crenulate (Fig. 2e); second suture deep, crenulate, straight medially, and becoming narrower laterally (Fig. 2e); T III and T IV coarsely sculptured, with distinct antero-lateral areas; T III–V with crenulate transverse subposterior groove (Fig. 2e); sculpture and antero-lateral areas of T V relatively weak; T VI weakly sculptured; T VII largely smooth except a few weak punctures; hypopygium acute apically, far beyond level of apex of metasoma; ovipositor sheath 0.98 × as long as fore wing.

***Colour*.** Largely black (Fig. 1); head largely pale yellow, except for antenna, eyes, stemmaticum and apex of mandible black (Fig. 2g); anteriorly and laterally mesoscutum black and remainder pale yellow; tegula, pronotum, propleuron, fore legs (except for black claws) and middle legs (except for coxae, trochanters, second-fifth segments of tibia and claws black, first segment of tibia infuscate apically) yellow (Fig. 2c, d, f); wing membrane infuscate, area below parastigma brownish, pterostigma and veins (except for forewing vein 1-SR+M half basally, 1-SR and 1-M dark brown) pale yellow (Fig. 2a, b).

***Variation*.** Length of body of female 8.0–11.1 mm, of fore wing of female 7.3–9.8 mm, and of ovipositor sheath 7.0–9.6 mm; length of mesosoma 2.4–3.4× its height; fore wing vein cu-a slightly postfurcal; hind wing vein SC+R1 1.5–1.9× longer than vein 1r-m; ovipositor sheath 0.96–0.98 × as long as fore wing; area below parastigma sometimes paler, only greyish brown.

#### Biology.

Unknown.

#### Distribution.

China (Yunnan).

#### Etymology.

Named after the short head, especially in anterior view: *brevis* is Latin for short and -*ceps* is Latin for head.

### Chaoilta himalayensis

Taxon classificationAnimaliaHymenopteraBraconidae

(Cameron, 1899)

6CBC6C42-970D-5547-957D-2F48D4CD8646

Figures 3 , 4

Bracon himalayensis Cameron, 1899: 70; Szépligeti 1904: 36; Ramakrishna Ayyar 1924: 354.
Chaoilta himalayensis (Cameron): Baltazar, 1972: 263; Shenefelt 1978: 1667; van Achterberg and O’Toole 1993: 7.

#### Material examined.

1♀, China, Hainan Prov., Qiongzhong, 400 m, 16.VII.1960, Li Suofu, No. IOZ(E)1964594 (IZCAS); 1♀, China, Hainan Prov., Tongshi, 340 m, 31.VII.1960, Li Suofu, No. IOZ(E)1964592 (IZCAS); 1♀, China, Hainan Prov., Mt. Wuzhi, 27.IV.1984, Gu Maobin, No. IOZ(E)1964583 (IZCAS); 1♀, China, Hainan Prov., Baisha, 18.III.1959, Jin Gentao, No. 34023664 (SHEM); 1♀, China, Yunnan Prov., Cheli, 540 m, 12.III.1957, Wang Shuyong, No. IOZ(E)1964542 (IZCAS).

#### Biology.

Unknown.

#### Distribution.

China (Hainan, Yunnan); India.

#### Note.

This species is newly recorded from China.

### Genus Cyanopterus

Taxon classificationAnimaliaHymenopteraBraconidae

Haliday, 1835

15126ED4-8B8D-538F-B5D9-25A51756D3B7

Figures 5 , 6 , 7 , 8 , 9 , 10 , 11 , 12

Cyanopterus Haliday, 1835: 22; Szépligeti 1904: 21; Telenga 1936: 343; Watanabe 1937: 21; Tobias 1971: 210; Shenefelt 1978: 1676; Marsh 1979: 170; Quicke 1987: 109. Type species: Ichneumonflavator Fabricius, 1793 (Monobasic).
Ipobracon Thomson, 1892: 1787 (as subgroup of Bracon Fabricius, 1804); Shenefelt 1978: 1808; Quicke 1987: 109. Type species: Braconnigrator Zetterstedt, 1838 (Original designation). Synonymised by Quicke 1985: 46.
Bracambus Thomson, 1892: 1787 (as subgroup of Bracon Fabricius, 1804); Shenefelt 1978: 1676; Quicke 1987: 109. Type species: Vipiolongipalpis Thomson, 1892 (Monobasic and Original designation) (= Ichneumonflavator Fabricius, 1804). Synonymised by Szépligeti 1904: 21.
Bracomorpha Papp, 1971: 276; Quicke 1985: 358, 1987: 104. Type species: Bracomorphatorkai Papp, 1971 (Monobasic). Syn. nov.
Cyanopteridea Viereck, 1911: 476; Shenefelt 1978: 1677; Quicke 1987: 109. Type species: Iphiaulaxclypeolus Szépligeti, 1905 (Original designation). Synonymised by Watanabe 1937: 21.
Coeloidimorpha Viereck, 1913: 558; Shenefelt 1978: 1677; Quicke 1987: 109. Type species: Bracon (Melanobracon) webbi Viereck, 1909 (Original designation) (= Braconlaevis Provancher, 1880). Synonymised by Muesebeck and Walkley 1951: 159.
Atanycolimorpha Viereck, 1913: 557; Shenefelt 1978: 1436; Quicke 1987: 109. Type species: Atanycolimorphawinnemanae Viereck, 1913 (Original designation) (= Braconprovancheri Dalla Torre, 1898). Synonymised by Quicke 1987: 109.
Notaulobracon Fahringer, 1929: 237; Shenefelt 1978: 1809. Type species: Braconnigrator Zetterstedt, 1838 (Original designation). Synonymised by van Achterberg 1997: 30.

#### Diagnosis.

Body medium-sized; terminal antennomere often strongly acute apically; in lateral view scapus without double margin at inner side apically and concave apico-laterally, ventrally longer than dorsally; eye glabrous, weakly emarginated; face smooth or superficially granulate, sometimes with a few sparse punctures; clypeus moderately narrow, often flattened and without dorsal carina; malar suture moderately developed, often with long and dense setae; labio-maxillary complex normal, not elongate; frons weakly depressed, with some setae and a median groove; mesosoma largely smooth and shiny; notauli present only anteriorly; scutellar sulcus narrow and crenulate; propodeum largely smooth, without medio-longitudinal carina or groove; angle between veins 1-SR and C+SC+R of fore wing more than 75°; fore wing vein 1-SR+M straight or slightly curved subbasally; fore wing vein cu-a interstitial or slightly postfurcal; hind wing vein SC+R1 longer than vein 1r-m; basal lobes of claws largely rounded; metasoma often largely smooth and shiny; length of T I less than 1.5× its apical width; T II usually with a large medio-basal area, and with oblique lateral grooves connected to wide sublateral grooves; antero-lateral grooves of T III often wide and short; T III–V with or without antero-lateral areas; ovipositor with dorsal nodus and ventral serrations subapically.

#### Biology.

Most species are larval ectoparasitoids of Coleoptera (especially Cerambycidae and Curculionidae, but also some species of Buprestidae and Bostrichidae), and of Lepidoptera (mainly Sesiidae, Pyralidae, Erebidae, and Tortricidae) (Webb 1909; Viereck 1912; Fahringer 1926, 1934; Ramakrishna Ayyar 1928; Myers 1932; Watanabe 1937; Györfi 1941; Grobler 1957; De Santis and Esquivel 1966; Fulmek 1968; Papp 1971; Tobias 1971, 1976, 1986; Uhthoff-Kaufmann 1990; Campadelli and Scaramozzino 1994; Cordo et al. 1995; Papp 2009; Wang et al. 2009; Yu et al. 2016).

#### Distribution.

Cosmopolitan.

#### Note.

Tobias and Belokobylskij (2000) divided this genus into three subgenera: *Cyanopterus* Haliday, 1835, *Ipobracon* Thomson, 1892, and *Paravipio* Papp, 1967; in this paper we include *Bracomorpha* Papp, 1971, as a subgenus; in China we have not yet found *Ipobracon* and *Cyanopterus* s. s.; and *Paravipio* is new to China. *Bracomorpha* may be easily confused with *Acampyloneurus* van Achterberg, 1992, but the latter has the lower ovipositor valve without teeth and the upper valve without nodus, T II with slightly converging sublateral depressions and the dorsal carina of the clypeus present. In addition, the type species has the first subdiscal cell of the fore wing distinctly (ca. 1.5×) higher than length of vein m-cu and scapus without apical ledge at inner side. In *Bracomorpha* the lower ovipositor valve has minute apical teeth and the upper valve has a minute nodus, the dorsal carina of the clypeus absent, T II with nearly parallel sublateral depressions or depressions largely absent, the first subdiscal cell is narrower than length of vein m-cu or subequal and scapus with more or less developed narrow apical ledge at inner side.

**Figure 5.**
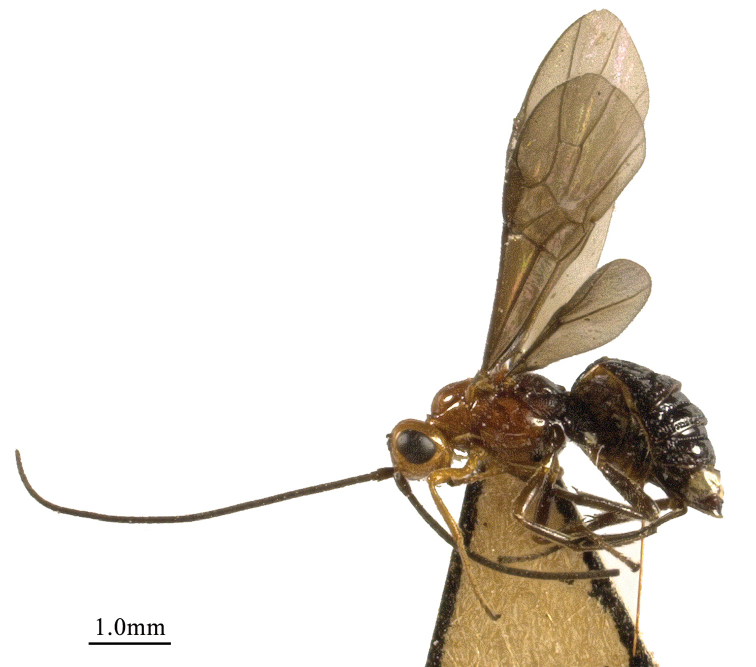
Cyanopterus (Bracomorpha) lucidus sp. nov., ♀, holotype, habitus, lateral view.

### Key to subgenera and Chinese species of the genus *Cyanopterus* Haliday

**Table:** 

1	Sublateral depressions of T II diverging posteriorly (Fig. 12e) (sometimes only anteriorly present and shallow); medio-basal area of T II narrow basally (Fig. 12e) (in *C.flavator* only basal part present); vein r of fore wing gradually merging into vein 3-SR (Fig. 12a)	**2**
–	Sublateral depressions of T II subparallel posteriorly (Figs 6e, 8e, 10e) (sometimes only present in anterior half of T); medio-basal area of T II wide basally (Figs 6e, 8e, 10e); vein r of fore wing more or less angled with vein 3-SR (Figs 6a, 8a, 10a)	**3**
2	Medio-basal area of T II complete (Fig. 12e); T III with distinct and smooth antero-lateral grooves (Fig. 12e); subgenus Paravipio Papp, 1967; [marginal cell remains distinctly removed from wing apex (Fig. 12a); second metasomal suture smooth (Fig. 12e)] [China; Korea; Russia]	***C.* (*P.*) *jakuticus***
–	Medio-basal area of T II only anteriorly developed; T III without distinct antero-lateral grooves; [not yet found in China]	**subgenusCyanopterus Haliday, 1835**
3	Antero-lateral grooves of T III absent; latero-basal triangular areas of T II not differentiated; [not yet found in China]	**subgenusIpobracon Thomson, 1892**
–	Antero-lateral grooves of T III present (Figs 6e, 8e, 10e); latero-basal triangular areas of T II distinctly differentiated (Figs 6e, 8e, 10e); subgenusBracomorpha Papp, 1971, stat. nov.	**4**
4	Medio-basal area of T II rounded apically, shield-shaped (Fig. 10e); T III–V without subposterior transverse groove (Fig. 10e); mesoscutum dark brown anteriorly and postero-laterally (Fig. 10d) [China; Russia; Ukraine]	**C. (B.) tricolor**
–	Medio-basal area of T II acute apically and triangular (Figs 6e, 8e); T III–V with distinct subposterior transverse grooves (Figs 6e, 8e); mesoscutum reddish yellow anteriorly and postero-laterally (Figs 6d, 8d)	**5**
5	Scape distinctly protruding ventrally and slender (Fig. 6i); triangular medio-basal area of T II strongly tapering apically (Fig. 6e); vein 1-SR+M of fore wing bent basally (Fig. 6a) [China]	**C. (B.) lucidus sp. nov.**
–	Scape not distinctly protruding ventrally and robust (Fig. 8i); triangular medio-basal area of T II gradually tapering apically (Fig. 8e); vein 1-SR+M of fore wing straight basally (Fig. 8a)	**6**
6	Head largely reddish yellow; median area of T I largely smooth except for coarsely sculptured posteriorly; subposterior transverse grooves of T III–V coarsely largely punctate especially laterally; fore leg largely dark yellow, but claws dark brown [China]	**C. (B.) ninghais**
–	Head largely black (Fig. 8g, h); median area of T I largely coarsely sculptured except for smooth anteriorly (Fig. 8j); subposterior transverse grooves of T III–V largely smooth (Fig. 8e); fore leg entirely dark brown [China]	**C. (B.) transversus sp. nov.**

### Cyanopterus (Bracomorpha) lucidus sp. nov.

Taxon classificationAnimaliaHymenopteraBraconidae

C764A216-0F9D-5181-965A-A797FEB37DB5

http://zoobank.org/575B3F79-DE6B-4363-AE30-D85326625C81

Figures 5 , 6

#### Material examined.

***Holotype***: ♀, China, Zhejiang Prov., Lin’an, Mt. Tianmu, 11.VI.1993, Wang Jianping, No. 935484 (ZJUH). Paratypes. 1♀, same data as holotype, No. 935465 (ZJUH); 1♀, China, Zhejiang Prov., Lin’an, Mt. Tianmu, 11.VI.1993, Xu Zaifu, No. 935252 (ZJUH).

#### Diagnosis.

This new species is very similar to C. (I.) praecinctus (Shestakov, 1936) [Korea; Russia], but can be separated from the latter by the following characters: in dorsal view length of eye 1.7× temple, temples distinctly narrowed behind eyes (in dorsal view length of eye 1.5× temple and temples weakly narrowed behind eyes in C. (I.) praecinctus); head with black dorsal marking small and narrowed to stemmaticum, not reaching frons (large and reaching frons); triangular medio-basal area of T II strongly tapering apically (gradually tapering apically).

#### Description.

Holotype, ♀, length of body 5.1 mm, of fore wing 4.7 mm, of ovipositor sheath 1.6 mm.

**Figure 6.**
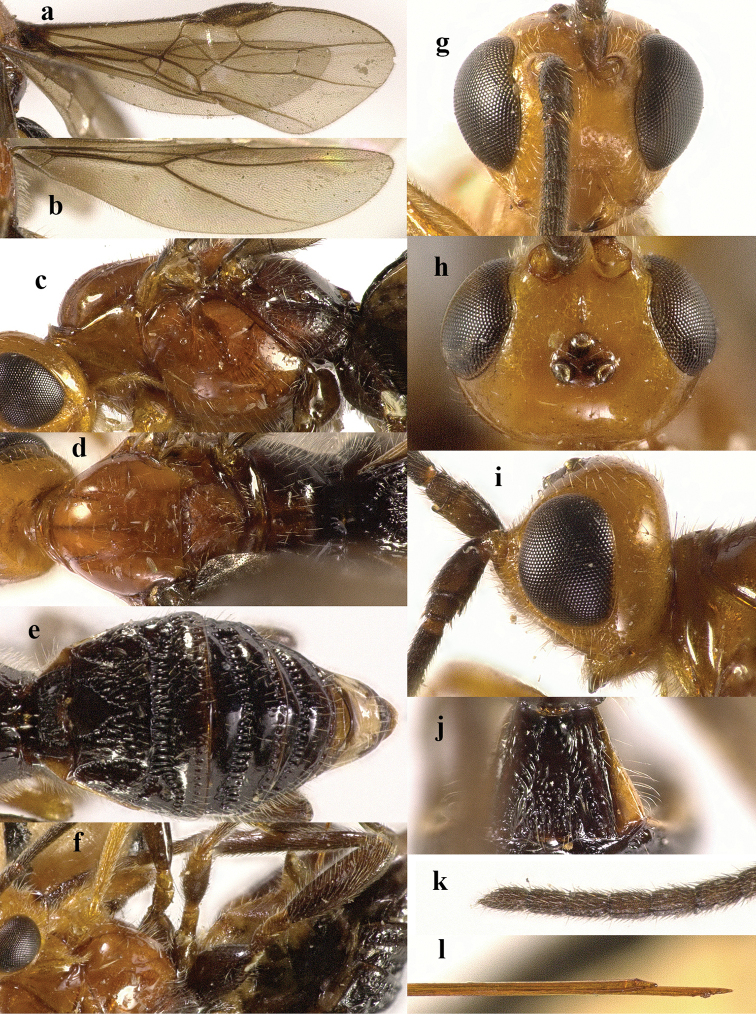
Cyanopterus (Bracomorpha) lucidus sp. nov., ♀, holotype **a** fore wing **b** hind wing **c** mesosoma, lateral view **d** mesosoma, dorsal view **e** metasoma, dorsal view **f** hind leg, lateral view **g** head, anterior view **h** head, dorsal view **i** head, lateral view **j** first metasomal tergite, dorsal view **k** apex of antenna **l** apex of ovipositor, lateral view.

***Head*.** Antenna with 42 antennomeres; terminal antennomere slender and acute, 2.3× longer than its maximum width (Fig. 6k); third antennomere 1.8× longer than its maximum width, 1.1 and 1.2× longer than fourth and fifth, respectively, the latter 1.4× longer than wide; malar suture with sparse short setae, and with fine punctures (Fig. 6i); clypeus height: inter-tentorial distance: tentorio-ocular distance = 3: 10: 7; clypeus with sparse long setae; eye weakly emarginated (Fig. 6g); face largely glabrous except for a few short setae, and with some sparse punctures (Fig. 6g); eye height: shortest distance between eyes: head width = 13: 13: 30; frons largely smooth, weakly concave behind antennal sockets, with a median groove (Fig. 6h); vertex smooth, but with some sparse short setae; minimum distance between posterior ocelli: minimum diameter of elliptical posterior ocellus: minimum distance between posterior ocellus and eye = 3: 4: 6; temples largely glabrous except for a few short setae, and directly narrowed behind eyes (Fig. 6h).

***Mesosoma*.** Length of mesosoma 1.4× its height (Fig. 6c); notauli impressed in anterior half of mesoscutum (Fig. 6d); mesoscutum smooth, with sparse setae (Fig. 6d); scutellar sulcus rather wide, moderately deep, and with crenulae (Fig. 6d); scutellum with dense short setae posteriorly; metanotum strongly convex medially, and with a short median carina anteriorly (Fig. 6d); propodeum smooth, without longitudinal carinae or groove, with sparse setae medially, and dense long setae laterally (Fig. 6d).

***Wings*.** Fore wing (Fig. 6a): SR1: 3-SR: r = 35: 23: 6; 1-SR+M weakly curved after arising from 1-M, and 1.5× longer than 1-M; 2-SR: 3-SR: r-m = 11: 23: 9; angle between 1-SR and C+SC+R ca. 85°; m-cu straight; 2-SR+M rather short; cu-a interstitial. Hind wing (Fig. 6b): SC+R1: 2-SC+R: 1r-m = 35: 7: 15.

***Legs*.** Length of fore femur: tibia: tarsus = 22: 26: 37; length of hind femur: tibia: basitarsus = 30: 44: 16; length of femur, tibia and basitarsus of hind leg 3.4, 8.0 and 5.3× their maximum width, respectively (Fig. 6f); hind tibial spurs 0.3 and 0.4× as long as hind basitarsus.

***Metasoma*.** Length of T I 1.1× its apical width, median area convex and sculptured (Fig. 6j); lateral grooves of T I sparsely crenulate (Fig. 6j); T II largely sculptured, but smooth posteriorly (Fig. 6e); triangular medio-basal area of T II large and smooth, with a few short oblique carinae connected laterally, and acute apically, but not attached with medio-longitudinal carina; antero-lateral areas of T II developed and smooth, anterior grooves moderately wide and crenulate (Fig. 6e); second suture deep and crenulate, wide and straight medially, narrow laterally (Fig. 6e); T III–V with antero-lateral areas, and crenulate transverse subposterior groove (Fig. 6e); T III–VII largely smooth, and with spare long setae posteriorly; hypopygium acute apically, not reaching level of apex of metasoma; ovipositor sheath 0.3× as long as fore wing.

***Colour*.** Head and mesosoma largely reddish yellow (Fig. 5); antenna (scapus and pedicellus paler), eyes, mandible apically, stemmaticum, metapleuron dorsally and propodeum (except medio-anteriorly) blackish brown (Fig. 6c, d, g, h); fore leg (except for telotarsus and claws dark brown) reddish yellow, middle and hind legs dark brown (Fig. 6f); metasoma and ovipositor sheath blackish brown (Figs 5, 6e); wing membrane infuscate, pterostigma and veins dark brown (Fig. 6a, b).

***Variation***. Length of body of female 5.1–6.2 mm, of fore wing of female 4.7–5.7 mm, and of ovipositor sheath 1.5–1.7 mm; antenna of female with 42–50 antennomeres; crenulate transverse subposterior grooves of T III–V are sometimes absent medially; scutellum, metanotum, metapleuron and propodeum sometimes uniformly black.

#### Biology.

Unknown.

#### Distribution.

China (Zhejiang).

#### Etymology.

Named after the shiny face: *lucidus* is Latin for shining.

### Cyanopterus (Bracomorpha) ninghais

Taxon classificationAnimaliaHymenopteraBraconidae

Wang, Chen, Wu & He, 2009 comb. nov.

6F946403-3A41-5F32-8E78-C5C37C9F3F9D

Bracomorpha ninghais Wang, Chen, Wu & He in Wang et al. 2009: 944.

#### Biology.

The type series have been reared from a larva of *Monochamusalternatus* Hope, 1842 (Coleoptera: Cerambycidae) (Wang et al. 2009).

#### Distribution.

China (Zhejiang).

#### Note.

Wang et al. (2009) first reported the species from Zhejiang (SE. China), but no specimens were available for our study.

### Cyanopterus (Bracomorpha) transversus sp. nov.

Taxon classificationAnimaliaHymenopteraBraconidae

413EADC5-65D1-5FC1-B824-A29E34C4E7D3

http://zoobank.org/44660842-83FC-482F-A63F-C39DC3DEA589

Figures 7 , 8

#### Material examined.

***Holotype***: ♀, China, Zhejiang Prov., Mt. Tianmu, 23.VI.1984, Zhu Xiliang, No. 842005 (ZJUH). Paratypes. 1♀, China, Zhejiang Prov., Anji, Mt. Longwang, 31.VIII.1983, Chen Xuexin, No. 939811 (ZJUH); 1♀, China, Zhejiang Prov., Mt. West Tianmu, 4.IX.1987, Chen Xuexin, No. 877070 (ZJUH); 1♀, China, Zhejiang Prov., Songyang, 18–31.VII.1989, He Junhua, No. 895329 (ZJUH); 1♀, China, Zhejiang Prov., Mt. West Tianmu, 3.IX.1987, Wang Xingeng, No. 876768 (ZJUH); 1♀, China, Henan Prov., Mt. Jigong, 11.VII.1997, Chen Xuexin, No. 973715 (ZJUH); 1♀, China, Fujian Prov., Mt. Meihua, 1000–1400m, 23–24.VII.1988, Fan Jinjiang, No. 886653 (ZJUH).

**Figure 7.**
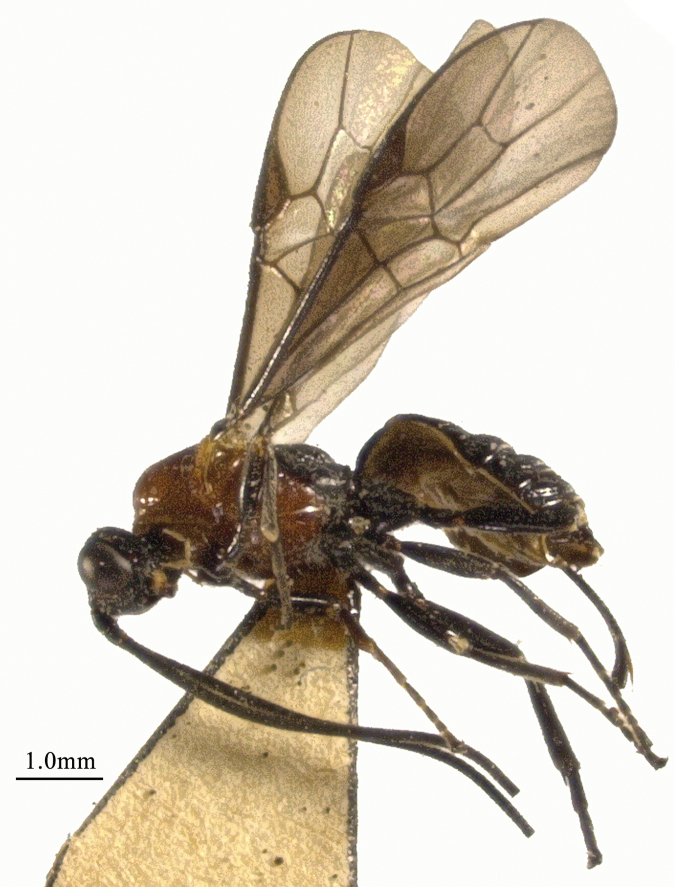
Cyanopterus (Bracomorpha) transversus sp. nov., ♀, holotype, habitus, lateral view.

#### Diagnosis.

This new species is very similar to C. (I.) bohayicus Belokobylskij, 2000 [Russia], but can be separated from the latter by the following characters: T I 1.0–1.1× longer than apical width (1.35 × in C. (I.) bohayicus); fore leg blackish brown (yellowish brown); triangular medio-basal area of T II gradually tapering apically (strongly tapering apically); ovipositor sheath 0.2–0.3× as long as fore wing (0.4×).

#### Description.

Holotype, ♀, length of body 6.2 mm, of fore wing 6.5 mm, of ovipositor sheath 1.6 mm.

**Figure 8.**
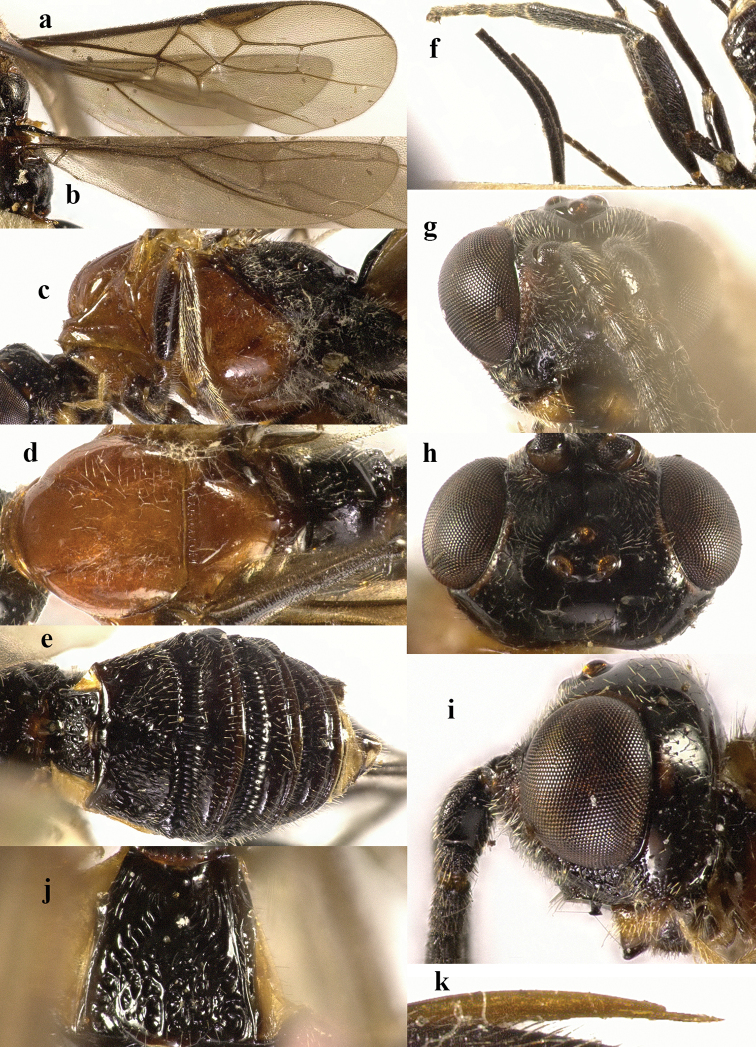
Cyanopterus (Bracomorpha) transversus sp. nov., ♀, holotype **a** fore wing **b** hind wing **c** mesosoma, lateral view **d** mesosoma, dorsal view **e** metasoma, dorsal view **f** hind leg, lateral view **g** head, anterior view **h** head, dorsal view **i** head, lateral view **j** first metasomal tergite, dorsal view **k** apex of ovipositor, lateral view.

***Head.*** Antenna with 55 antennomeres; apical antennomere acute, 2.4× longer than its maximum width; third antennomere 1.6× longer than its maximum width, 1.3 and 1.4× longer than fourth and fifth antennomers, respectively, the latter 1.1× longer than wide; malar suture with dense short setae (Fig. 8i); clypeus height: inter-tentorial distance: tentorio-ocular distance = 3: 8: 5; clypeus with sparse long setae; eye weakly emarginated (Fig. 8g); face granulate, with dense and long setae (Fig. 8g); eye height: shortest distance between eyes: head width = 14: 17: 32; frons largely smooth, weakly concave behind antennal sockets, with a strong median groove (Fig. 8h); vertex smooth, but with short setae especially laterally; minimum distance between posterior ocelli: minimum diameter of elliptical posterior ocellus: minimum distance between posterior ocellus and eye = 1: 1: 2; temples largely smooth except for a few weak punctures, with sparse setae, and directly narrowed behind eyes (Fig. 8h).

***Mesosoma.*** Length of mesosoma 1.5× its height (Fig. 8c); notauli impressed in anterior half of mesoscutum (Fig. 8d); mesoscutum smooth, with some sparse setae (Fig. 8d); scutellar sulcus rather wide, moderately deep, and with crenulae (Fig. 8d); scutellum with dense short setae posteriorly; metanotum strongly convex medially, and with a short median carina anteriorly (Fig. 8d); propodeum smooth, without longitudinal carinae or groove, with sparse setae medially, and with dense long setae laterally (Fig. 8d).

***Wings*.** Fore wing (Fig. 8a): SR1: 3-SR: r = 42: 31: 7; 1-SR+M more or less straight, and 1.6× longer than 1-M; 2-SR: 3-SR: r-m = 13: 31: 11; angle between 1-SR and C+SC+R ca. 80°; m-cu straight; 2-SR+M rather short; cu-a slightly postfurcal. Hind wing (Fig. 8b): SC+R1: 2-SC+R: 1r-m = 31: 8: 17.

***Legs*.** Length of fore femur: tibia: tarsus = 25: 27: 41; length of hind femur: tibia: basitarsus = 33: 46: 18; length of femur, tibia and basitarsus of hind leg 3.7, 7.7 and 4.5× their maximum width, respectively (Fig. 8f); hind tibial spurs 0.35 and 0.40 × as long as hind basitarsus.

***Metasoma*.** Length of T I equal to its apical width, median area convex and coarsely sculptured (Fig. 8j); lateral grooves of T I strongly crenulate (Fig. 8j); T II largely sculptured except posteriorly (Fig. 8e); triangular medio-basal area of T II large and smooth, with some short oblique carinae laterally, and acute apically, but without medio-longitudinal carina; antero-lateral areas of T II developed and smooth, anterior grooves moderately wide and sparsely crenulate (Fig. 8e); second suture deep and crenulate, wide and straight medially, narrow laterally (Fig. 8e); T III–V with antero-lateral areas, and crenulate transverse subposterior groove (Fig. 8e); T III–VII largely smooth, and with sparse short setae; hypopygium acute apically, not reaching level of apex of metasoma; ovipositor sheath 0.2× as long as fore wing.

***Colour*.** Head largely black, mandible (except for apically) and maxillary palps basally yellowish brown, surrounding area of eyes reddish yellow (Fig. 8g, h); mesosoma largely reddish yellow (Fig. 8c), metanotum, metapleuron and propodeum black (Fig. 8c, d); legs, metasoma and ovipositor sheath black (Figs 7, 8e, f); wing membrane greyish brown, pterostigma and veins dark brown (Fig. 8a, b).

***Variation*.** Length of body of female 5.6–6.5 mm, of fore wing of female 6.0–6.9 mm, and of ovipositor sheath 1.4–2.3 mm; ovipositor sheath 0.5–0.6× as long as fore wing; length of mesosoma 1.4–1.7× its height; length of T I 1.0–1.1× its apical width; ovipositor sheath 0.2–0.3× as long as fore wing; fore femur and tibia sometimes somewhat reddish yellow.

#### Biology.

Unknown.

#### Distribution.

China (Henan, Fujian, Zhejiang).

#### Etymology.

Named after the transverse head, especially so in dorsal view: *transversus* is Latin for transverse.

### Cyanopterus (Bracomorpha) tricolor

Taxon classificationAnimaliaHymenopteraBraconidae

(Ivanov, 1896)

9C556369-5B48-52DB-964B-FEA3F0CE48E5

Figures 9 , 10

Iphiaulax tricolor Ivanov, 1896: 177; Szépligeti 1904: 22.
Iphiaulacidea tricolor (Ivanov): Fahringer, 1926: 212.
Ipobracon tricolor (Ivanov): Telenga, 1936: 96; Tobias 1971: 207; Shenefelt 1978: 1936.
Cyanopterus (Ipobracon) tricolor (Ivanov): Tobias & Belokobylskij, 2000: 176.

#### Material examined.

9♀♀, 17♂♂, China, Heilongjiang Prov., Yichun, ?.?.1985, Jin Liyuan, No. 864299 (five specimens), 864298 (three specimens), 864300 (five specimens), 864301(six specimens), 864732 (two specimens), 864735, 864736 (three specimens), 864738 (ZJUH); 2♀♀, 1♂, China, Helongjiang Prov., Daicen, 24.VII.1977, He Junhua, No. 771723, 771674, 771671 (ZJUH); 1♀, 2♂♂, China, Jilin Prov., Mt. Changbai, 10.VIII.1977, He Junhua, No. 771350, 771486, 771480 (ZJUH).

**Figure 9.**
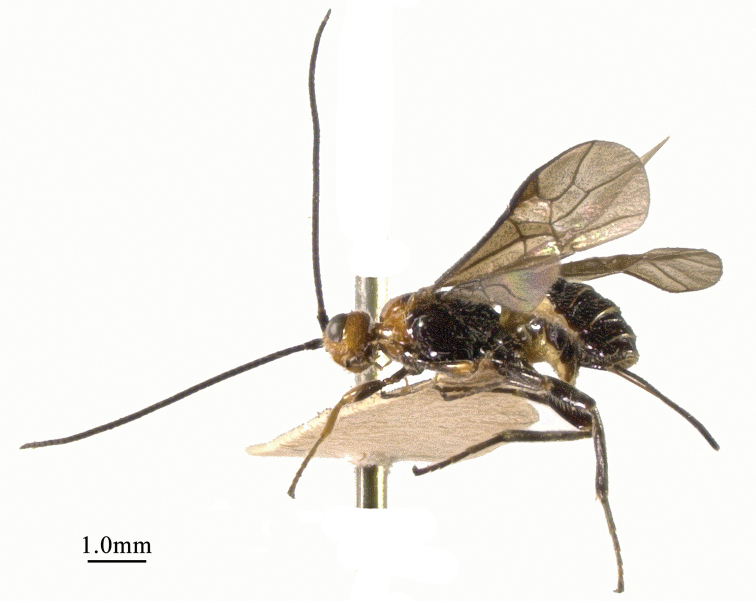
Cyanopterus (Bracomorpha) tricolor (Ivanov, 1896), ♀, habitus, lateral view.

#### Biology.

Unknown.

#### Distribution.

China (Heilongjiang, Jilin); Russia; Ukraine.

#### Note.

This species is newly recorded from China.

**Figure 10.**
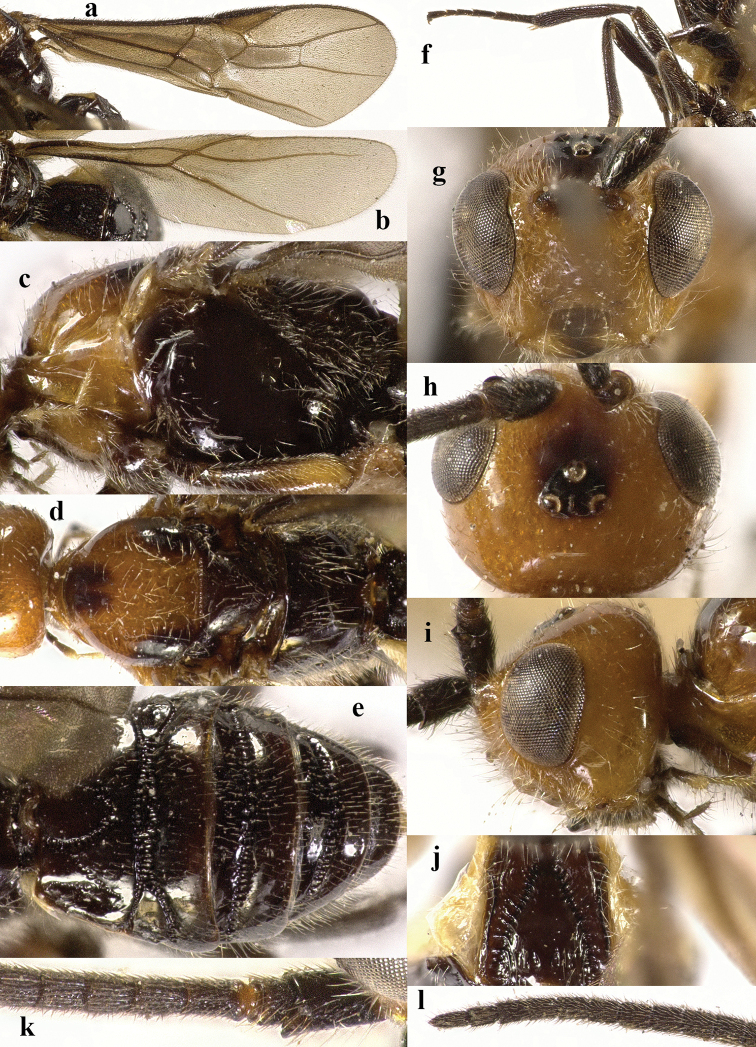
Cyanopterus (Bracomorpha) tricolor (Ivanov, 1896). ♀ **a** fore wing **b** hind wing **c** mesosoma, lateral view **d** mesosoma, dorsal view **e** metasoma, dorsal view **f** hind leg, lateral view **g** head, anterior view **h** head, dorsal view **i** head, lateral view **j** first metasomal tergite, dorsal view **k** scapus outer side, lateral view **l** apex of antenna.

### Cyanopterus (Paravipio) jakuticus

Taxon classificationAnimaliaHymenopteraBraconidae

(Tobias, 1973)

E1D4DBD6-6F82-5B7A-8AD4-134077A2ACC9

Figures 11 , 12

Ipobracon jakuticus Tobias in Tobias and Abdinbekova 1973: 435; Shenefelt 1978: 1821.
Cyanopterus (Ipobracon) jakuticus (Tobias): Papp, 1996: 155.
Cyanopterus (Paravipio) jakuticus (Tobias): Tobias & Belokobylskij, 2000: 171.

#### Material examined.

1♀, China, Jilin Prov., Mt. Changbai, 10.VIII.1977, He Junhua, No. 771330 (ZJUH); 1♂, China, Helongjiang Prov., Daicen, 24.VII.1977, He Junhua, No. 771793 (ZJUH).

#### Biology.

Unknown.

#### Distribution.

China (Heilongjiang, Jilin); Korea; Russia.

#### Note.

This species is newly recorded from China.

**Figure 11.**
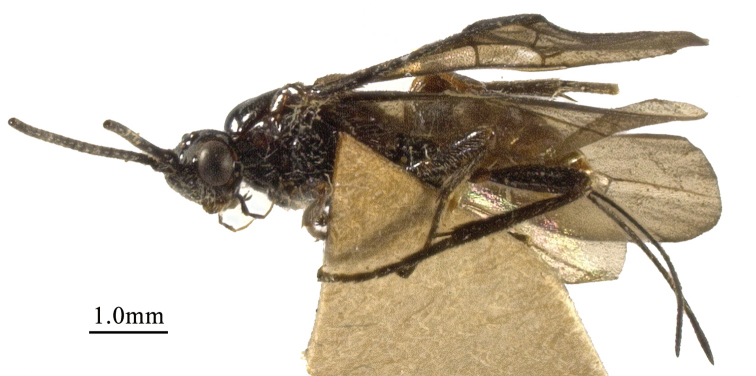
Cyanopterus (Paravipio) jakuticus (Tobias, 1973), ♀, habitus, lateral view.

**Figure 12.**
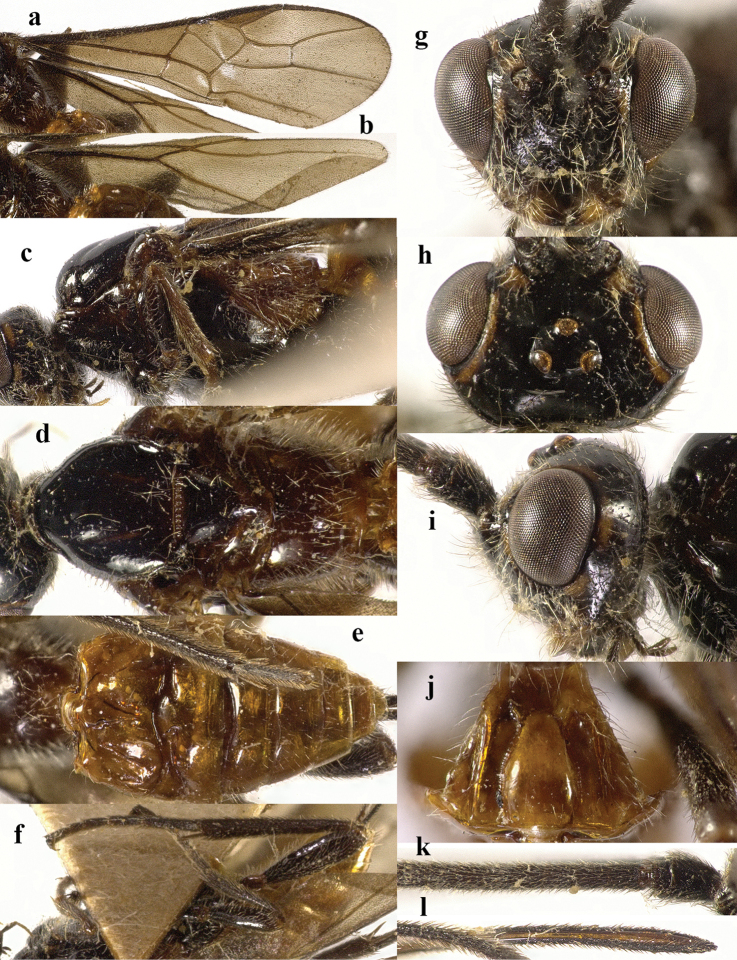
Cyanopterus (Paravipio) jakuticus (Tobias, 1973). ♀. **a** fore wing **b** hind wing **c** mesosoma, lateral view **d** mesosoma, dorsal view **e** metasoma, dorsal view **f** hind leg, lateral view **g** head, anterior view **h** head, dorsal view **i** head, lateral view **j** first metasomal tergite, dorsal view **k** scapus outer side, lateral view **l** apex of ovipositor, lateral view.

### Genus Gammabracon

Taxon classificationAnimaliaHymenopteraBraconidae

Quicke, 1984

E7F4D6CA-25CB-5A09-BFFF-8008562BED4E

**Figure 13.**
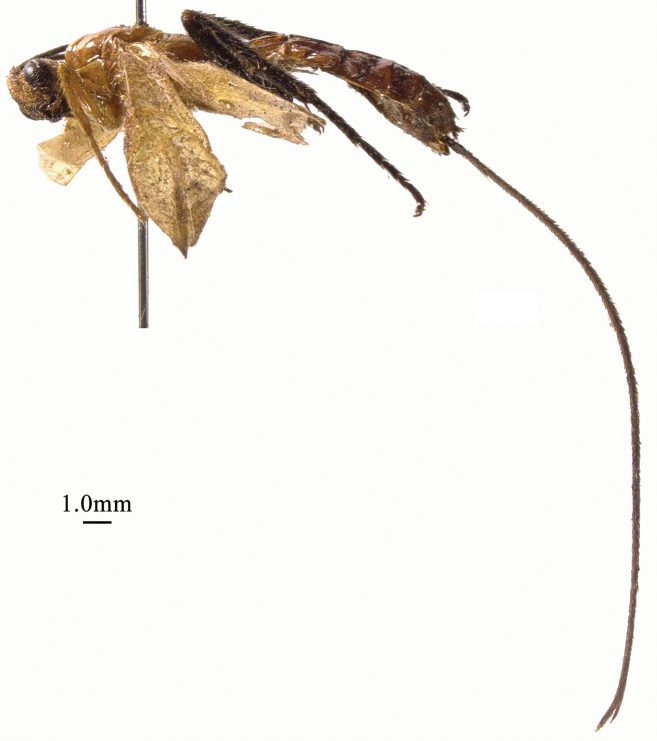
*Gammabraconuniformis* sp. nov., ♀, holotype, habitus, lateral view.

**Figure 14.**
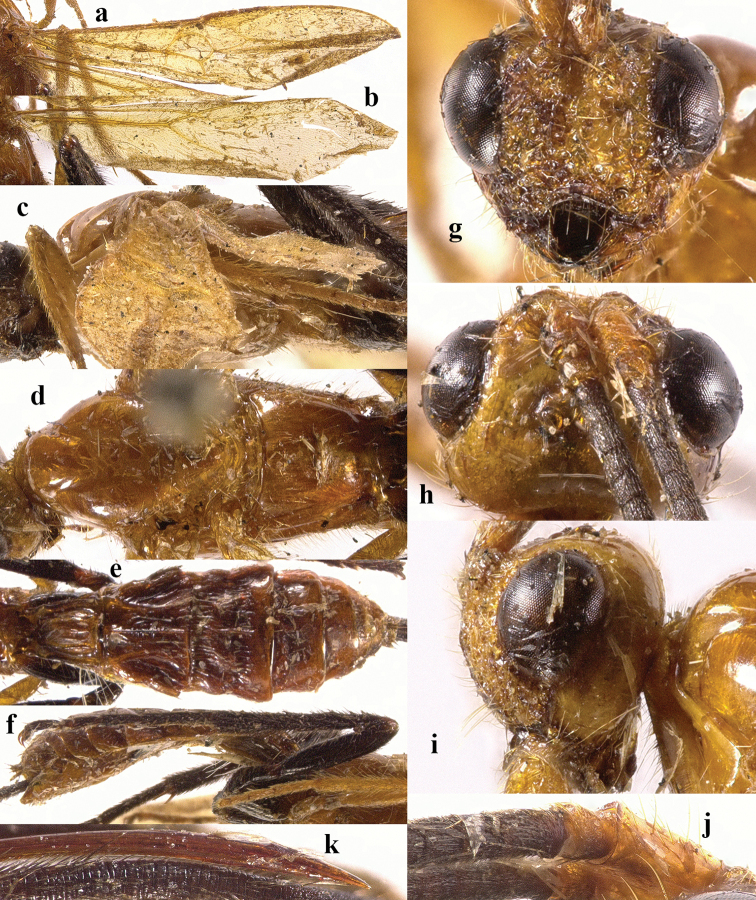
*Gammabraconuniformis* sp. nov., ♀, holotype **a** fore wing **b** hind wing **c** mesosoma, lateral view **d** mesosoma, dorsal view **e** metasoma, dorsal view **f** hind leg, lateral view **g** head, anterior view **h** head, dorsal view **i** head, lateral view **j** scapus outer side, lateral view **k** apex of ovipositor, lateral view.

**Figure 15.**
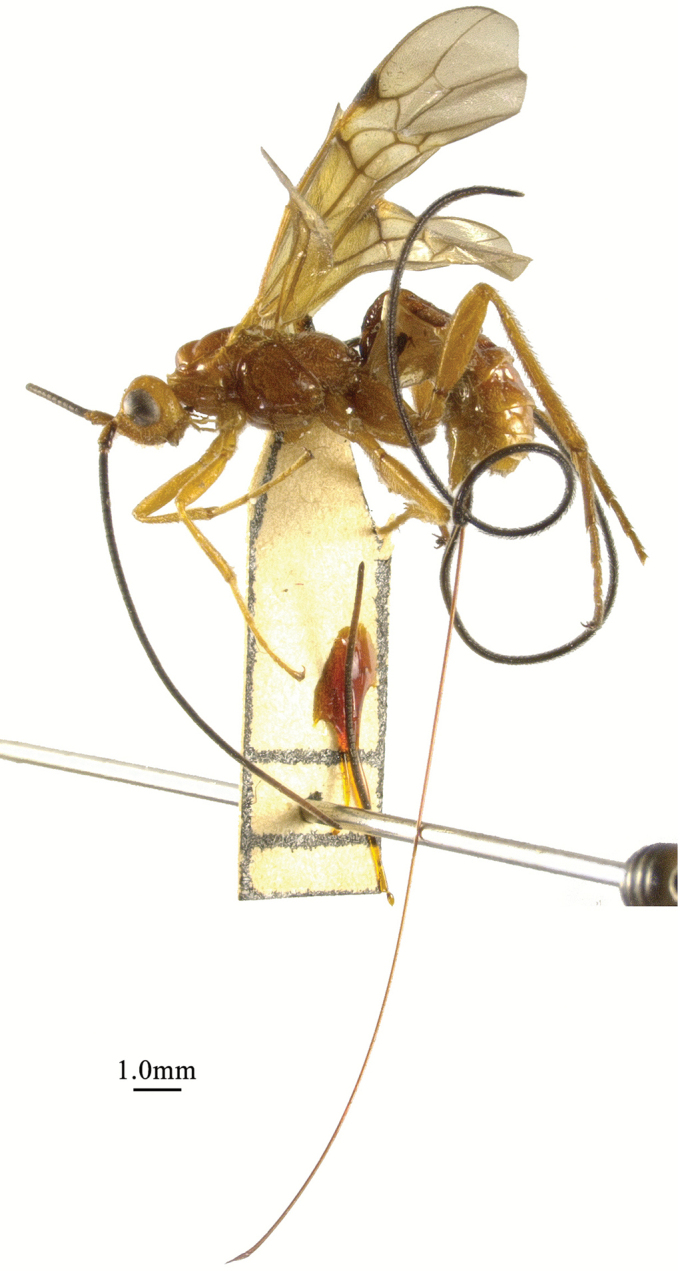
*Gammabraconwangi* sp. nov., ♀, holotype, habitus, lateral view.

**Figure 16.**
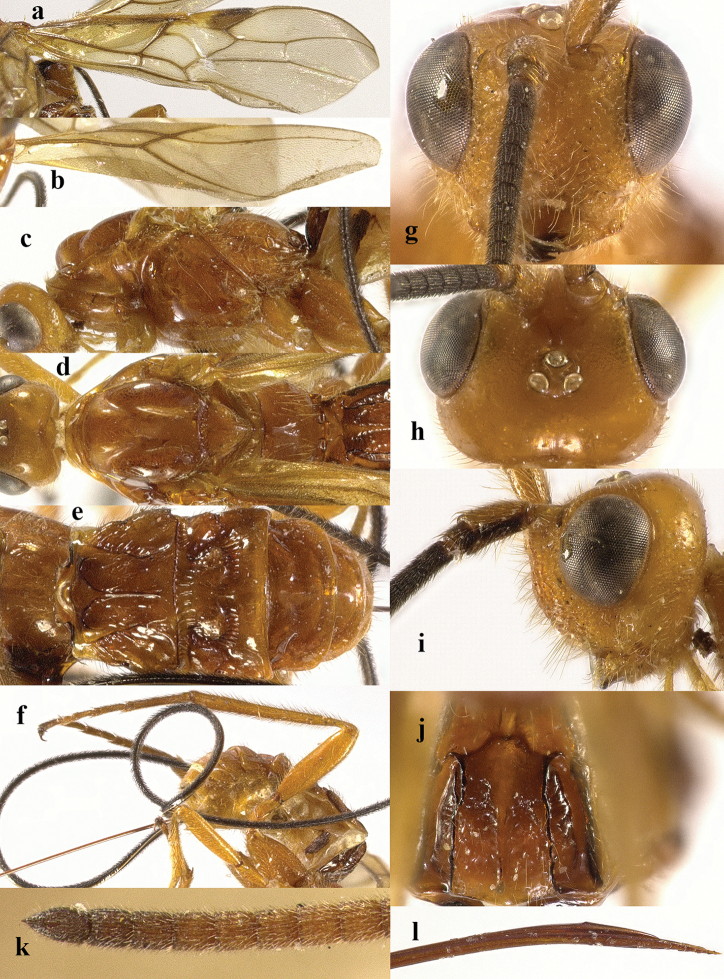
*Gammabraconwangi* sp. nov., ♀, holotype. **a** fore wing **b** hind wing **c** mesosoma, lateral view **d** mesosoma, dorsal view **e** metasoma, dorsal view **f** hind leg, lateral view **g** head, anterior view **h** head, dorsal view **i** head, lateral view **j** first metasomal tergite, dorsal view **k** apex of antenna **l** apex of ovipositor, lateral view.

Figures 13 , 14 , 15 , 16

Gammabracon Quicke, 1984a: 73, 1987: 113. Type species: Gammabraconscrobi Quicke, 1984 (Monobasic and original designation).

#### Diagnosis.

Body large; terminal antennomere often strongly acute apically; median antennomeres usually weakly wider than long; in lateral view scapus without double margin or with narrow ledge at inner side apically and slightly concave apico-laterally, ventrally longer than dorsally; eye glabrous, not or weakly emarginate; face strongly sculptured, depressed below and between the antennal sockets; clypeus moderately narrow, rugose and often without dorsal carina; malar suture moderately developed, often rugose; labio-maxillary complex normal, not elongate; frons strongly depressed, with a weak median groove; middle lobe of mesoscutum protruding strongly in front of lateral lobes; notauli developed and complete; scutellar sulcus narrow and crenulate; scutellum sometimes with an emargination medio-anteriorly; metanotum convex medially, and sometimes with a short and somewhat protruding median carina; propodeum often smooth, without medio-longitudinal carina or groove; angle between veins 1-SR and C+SC+R of fore wing more than 75°; vein 1-SR+M of fore wing evenly and strongly arched, forms with bases of vein 1-SR+M and 1-M a widened inverted “Y”; vein m-cu of fore wing widened; second submarginal cell of fore wing relatively long and parallel-sided; vein cu-a of fore wing interstitial or slightly postfurcal; hind wing vein 1r-m often distinctly shorter than SC+R1; claws simple; T I with parallel angulate sides of medial area, and comparatively flat, usually with lateral and medio-longitudinal carinae; T II usually with a triangular medio-basal triangular area connected to a medio-longitudinal carina apically, but absent near posterior margin of T II; second suture crenulate; hypopygium rather acute apically, usually beyond level of apex of metasoma; ovipositor normal, distinctly longer than body, subapically upper valve with nodus, and its lower valve with teeth ventrally.

#### Biology.

Unknown.

#### Distribution.

Oriental.

#### Note.

This genus is new to China.

### Key to Chinese species of the genus *Gammabracon* Quicke

**Table:** 

1	Pterostigma uniformly yellow (Fig. 14a); hind legs blackish brown (Fig. 14f); hind wing vein SC+R1 1.7× longer than vein 1r-m (Fig. 14b); hind femur 4.8× as long as maximal width (Fig. 14f); fore wing vein cu-a interstitial (Fig. 14a); medio-basal area of T II relatively small (Fig. 14e) [China]	***G.uniformis* sp. nov.**
–	Basal third of pterostigma yellow, and apical 2/3 dark brown (Fig. 16a); hind legs yellow, tarsus infuscate (Fig. 16f); hind wing vein SC+R1 2.2× longer than vein 1r-m (Fig. 16b); hind femur 3.9× as long as maximal width (Fig. 16f); fore wing vein cu-a slightly postfurcal (Fig. 16a); medio-basal area of T II relatively large (Fig. 16e) [China]	***G.wangi* sp. nov.**

### Gammabracon uniformis sp. nov.

Taxon classificationAnimaliaHymenopteraBraconidae

94FBA6E9-FA6F-50A4-B30F-C9F218C30D7E

http://zoobank.org/F790A8C5-5EB7-44AA-A599-17FF139CA41D

Figures 13 , 14

#### Material examined.

***Holotype***: ♀, China, Hainan Prov., Jianfengling, 6.V.1983, Gu Maobin, No. IOZ(E)1964585 (IZCAS).

#### Diagnosis.

This new species is very similar to *Gammabraconscrobi* Quicke, 1984 [Indonesia], but can be separated from the latter by the following characters: wing membrane uniformly yellow (basal half yellow and apical half greyish brown in *G.scrobi*); anterior margin of scutellum with a shallow pit and metanotum without short carina anteriorly (anterior margin of scutellum with a deep pit and metanotum with short carina anteriorly); apical half of medio-longitudinal carina of T I absent (medio-longitudinal carina of T I complete); T IV striate anteriorly, remainder of tergite smooth (entirely smooth).

#### Description.

Holotype, ♀, length of body 13.2 mm, of fore wing 12.2 mm, of ovipositor sheath 20.7 mm.

***Head*.** Antenna incomplete, with 48 antennomeres remaining; third antennomere 1.5 and 1.6× longer than fourth and fifth, respectively; third and fourth antennomeres 1.8 and 1.3× longer than wide, respectively; length of maxillary palp 0.8× height of head; malar suture with sparse short setae (Fig. 14i); clypeus height: inter-tentorial distance: tentorio-ocular distance = 7: 11: 9; clypeus coarsely rugose, with sparse long setae; eye not emarginated (Fig. 14g); face coarsely sculptured, with some sparse and long setae (Fig. 14g); frons smooth, distinctly depressed behind antennal sockets, with a median groove (Fig. 14h); vertex smooth, with a few short setae; minimum distance between posterior ocelli: minimum diameter of elliptical posterior ocellus: minimum distance between posterior ocellus and eye = 2: 3: 8; temples largely smooth except for a few weak punctures, and with sparse short setae laterally, subparallel immediately behind eyes (Fig. 14h); in dorsal view length of eye 1.7× temple.

***Mesosoma*.** Length of mesosoma 1.7× its height (Fig. 14c); notauli distinctly impressed (Fig. 14d); scutellar sulcus wide and deep, with crenulae (Fig. 14d); scutellum with a weak emargination medio-anteriorly, and with some short setae posteriorly; metanotum convex medially, but without median carina anteriorly; propodeum largely smooth except for a few crenulae posteriorly, with sparse setae medially, and with dense long setae laterally (Fig. 14d).

***Wings*.** Fore wing (Fig. 14a): SR1: 3-SR: r = 43: 26: 8; m-cu 1.2× longer than 3-CU1; cu-a interstitial. Hind wing (Fig. 14b): SC+R1 1.7× longer than 1r-m; anterior margin nearly not concave beyond the subbasal cell.

***Legs*.** Length of fore femur: tibia: tarsus = 37: 43: 57; length of hind femur: tibia: basitarsus = 44: 63: 26; length of femur, tibia and basitarsus of hind leg 4.8, 10.5 and 6.5× their maximum width, respectively (Fig. 14f); hind tibial spurs 0.3 and 0.2× as long as hind basitarsus.

***Metasoma*.** Length of T I 1.4× its apical width, median area convex and with a few longitudinal carinae (Fig. 14e); lateral grooves of T I smooth (Fig. 14e); T II strongly longitudinally rugose but antero-lateral areas smooth (Fig. 14e); medio-basal area of T II connected to medio-longitudinal carina apically but absent near posterior margin of T II, medio-longitudinal carina with some transverse crenulae laterally; antero-lateral areas of T II rather small, anterior grooves wide, with a few sparse crenulae (Fig. 14e); second suture deep and wide, with crenulae, more or less straight medially (Fig. 14e); T III longitudinally rugose but posteriorly and antero-lateral areas smooth, with a strong medio-longitudinal carina not reaching posterior margin of T, median area weakly raised and posteriorly defined by a deep transverse crenulate groove; T IV largely smooth but longitudinally rugose medially; T III and T IV with antero-lateral areas and grooves (T V weak); T V–VII smooth; hypopygium rather acute apically, reaching just beyond the level of apex of metasoma; ovipositor sheath 1.7× longer than fore wing.

***Colour*.** Largely yellow (Fig. 13); antennomeres except scape and pedicel, eye, mandible apically, fore and middle tarsi apically, hind leg, claws, ovipositor sheath black (Figs 13, 14g, f); metasomal tergites reddish yellow (Fig. 14e); wing membrane, pterostigma, and veins yellow (Fig. 14a, b).

#### Biology.

Unknown.

#### Distribution.

China (Hainan).

#### Etymology.

Named after the all yellow wing membrane, pterostigma and veins: *uniformis* is Latin for uniform.

### Gammabracon wangi sp. nov.

Taxon classificationAnimaliaHymenopteraBraconidae

23C86D05-2063-5B24-A9E0-E3FDCB975C20

http://zoobank.org/ADE461FA-1F50-4F3D-A55A-B2705BDB5369

Figures 15 , 16

#### Material examined.

***Holotype***: ♀, China, Fujian Prov., Kangshang, 9.IX.1993, Wang Jiashe, No. 854314 (ZJUH).

#### Diagnosis.

This new species is very similar to *Gammabraconuniformis* sp. nov., but can be separated from the latter by the following characters: basal third of pterostigma yellow, and apical 2/3 dark brown (uniformly yellow in *G.uniformis*); hind leg yellow, tarsus infuscate (blackish brown); hind wing vein SC+R1 2.2× longer than vein 1r-m (1.7× vein 1r-m); hind femur 3.9× as long as its maximum width (4.8×); fore wing vein cu-a slightly postfurcal (interstitial); medio-basal area of T II relatively large (small).

#### Description.

Holotype, ♀, length of body 10.5 mm, of fore wing 9.7 mm, of ovipositor sheath 17.1 mm.

***Head*.** Antenna with 61 antennomeres; apical antennomere strongly acute, 1.9× longer than its maximum width (Fig. 16k); penultimate antennomere 1.3× longer than its maximum width, and 0.7× as long as apical antennomere; median antennomeres 1.2× longer than wide; third antennomere 1.3 and 1.4× longer than fourth and fifth, respectively; third and fourth antennomeres 1.8 and 1.4× longer than wide, respectively; length of maxillary palp 0.9× height of head; malar suture with dense short setae (Fig. 16i); clypeus height: inter-tentorial distance: tentorio-ocular distance = 6: 11: 10; clypeus coarsely rugose, with sparse long setae; eye weakly emarginate (Fig. 16g); face coarsely sculptured, with some sparse and long setae (Fig. 16g); frons smooth, distinctly depressed behind antennal sockets, with a median groove (Fig. 16h); vertex smooth, with a few short setae; minimum distance between posterior ocelli: minimum diameter of elliptical posterior ocellus: minimum distance between posterior ocellus and eye = 5: 7: 18; temples largely smooth except for a few weak punctures, and with sparse long setae laterally, subparallel immediately behind eyes (Fig. 16h); in dorsal view length of eye 1.6× temple.

***Mesosoma*.** Length of mesosoma 1.8× its height (Fig. 16c); notauli distinctly impressed (Fig. 16d); scutellar sulcus wide and deep, with crenulae (Fig. 16d); scutellum with a weak emargination medio-anteriorly, and with some short setae posteriorly; metanotum convex medially, but without median carina anteriorly; propodeum largely smooth except for a few crenulae posteriorly, with sparse setae medially, and with dense long setae laterally (Fig. 16d).

***Wings*.** Fore wing (Fig. 16a): SR1: 3-SR: r = 47: 31: 8; 2-SR: 3-SR: r-m = 19: 31: 10; m-cu 1.4× longer than 3-CU1; cu-a weakly postfurcal. Hind wing (Fig. 16b): SC+R1 2.2× longer than 1r-m; anterior margin weakly concave beyond the subbasal cell.

***Legs*.** Length of fore femur: tibia: tarsus = 34: 38: 52; length of hind femur: tibia: basitarsus = 45: 77: 28; length of femur, tibia and basitarsus of hind leg 3.9, 11.8 and 9.3× their maximum width, respectively (Fig. 16f); hind tibial spurs 0.3 and 0.2× as long as hind basitarsus.

***Metasoma*.** Length of T I 1.1× its apical width, median area convex and coarsely sculptured but posteriorly smooth, with a medio-longitudinal carina posteriorly (Fig. 16j); lateral grooves of T I smooth (Fig. 16j); T II largely smooth, but with some oblique carinae besides medio-longitudinal carina (Fig. 16e); T II medio-basal area connected to medio-longitudinal carina apically, but absent near posterior margin of T; antero-lateral areas of T II small, strongly acute apically, anterior grooves wide, with some strong crenulae (Fig. 16e); second suture deep and wide, with crenulae, more or less straight medially (Fig. 16e); T III largely smooth, median area strongly raised and posteriorly defined by a deep sinuate transverse crenulate groove, median area with a few weak punctures laterally; T IV with transverse depression medially; T III and T IV with antero-lateral areas and grooves (of T V weak); T IV–VII smooth; hypopygium rather acute apically, protruding just beyond level of apex of metasoma; ovipositor sheath 1.8× longer than fore wing.

***Colour*.** Largely yellow (Fig. 15); antenna black (scapus yellow, with black stripe outer side) apical antennomeres (except for first-third antennomeres black) reddish yellow (Fig. 16i, k); eye, mandible apically, claws, part of dorsal carina and dorso-lateral carinae of T I, medio-longitudinal carina and lateral margins of medio-basal area of T II, ovipositor sheath black (Figs 15, 16e, g, j); hind tarsus infuscate (Fig. 16f); wing membrane yellow, pterostigma dark brown but basally yellow, veins largely brown, but fore wing vein 1-SR+M yellow (basally narrowly yellowish brown) (Fig. 16a, b).

#### Biology.

Unknown.

#### Distribution.

China (Fujian).

#### Etymology.

Named after the name of the collector of holotype.

### Genus Ischnobracon

Taxon classificationAnimaliaHymenopteraBraconidae

Baltazar, 1963

42114741-DB4F-57AD-A4FB-19902502DD70

**Figure 17.**
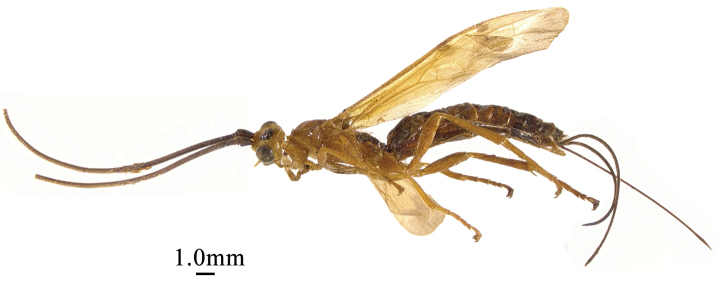
*Ischnobraconguttatus* sp. nov., ♀, holotype, habitus, lateral view.

**Figure 18.**
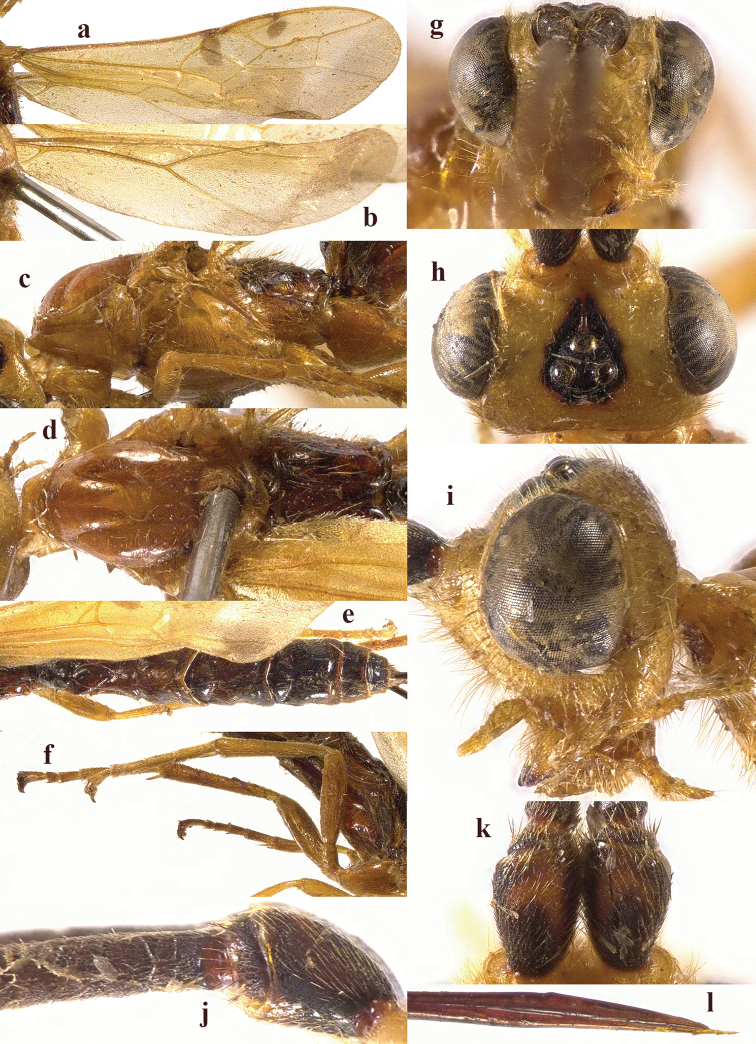
*Ischnobraconguttatus* sp. nov., ♀, holotype **a** fore wing **b** hind wing **c** mesosoma, lateral view **d** mesosoma, dorsal view **e** metasoma, dorsal view **f** hind leg, lateral view **g** head, anterior view **h** head, dorsal view **i** head, lateral view **j** scapus outer side, lateral view **k** scapus outer side, dorsal view **l** apex of ovipositor, lateral view.

**Figure 19.**
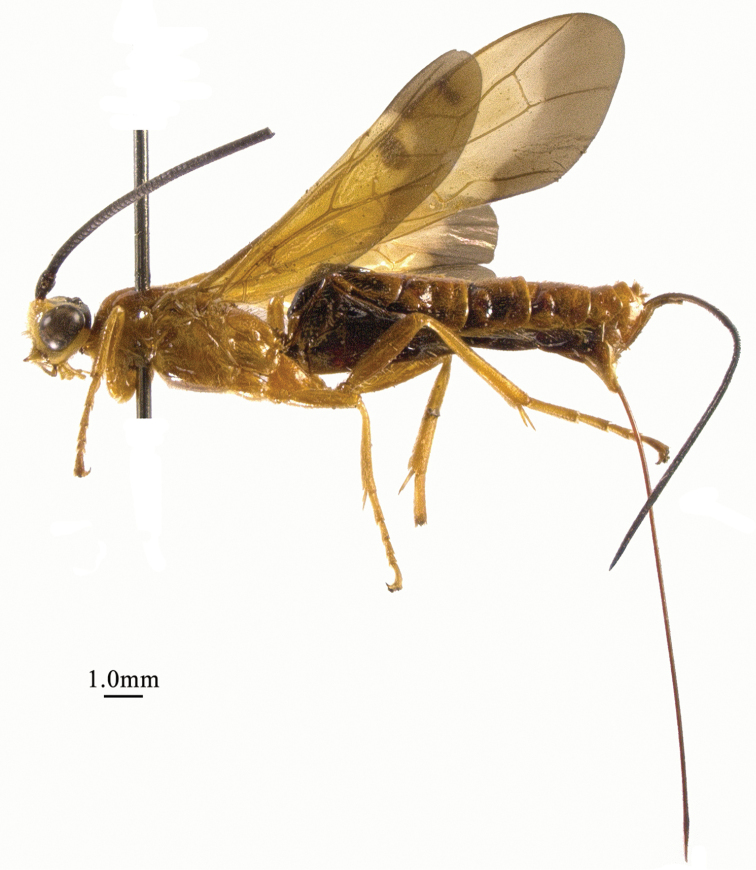
*Ischnobraconhannongbuai* Quicke et Butcher, 2010, ♀, habitus, lateral view.

**Figure 20.**
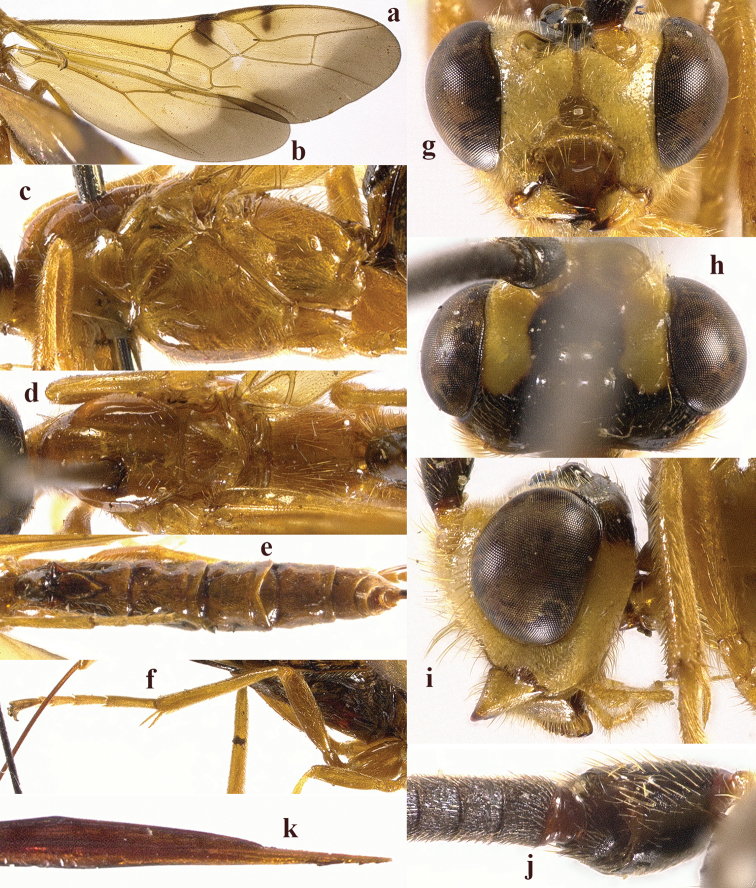
*Ischnobraconhannongbuai* Quicke et Butcher, 2010. ♀ **a** fore wing **b** hind wing **c** mesosoma, lateral view **d** mesosoma, dorsal view **e** metasoma, dorsal view **f** hind leg, lateral view **g** head, anterior view **h** head, dorsal view **i** head, lateral view **j** scapus outer side, lateral view **k** apex of ovipositor, lateral view.

**Figure 21.**
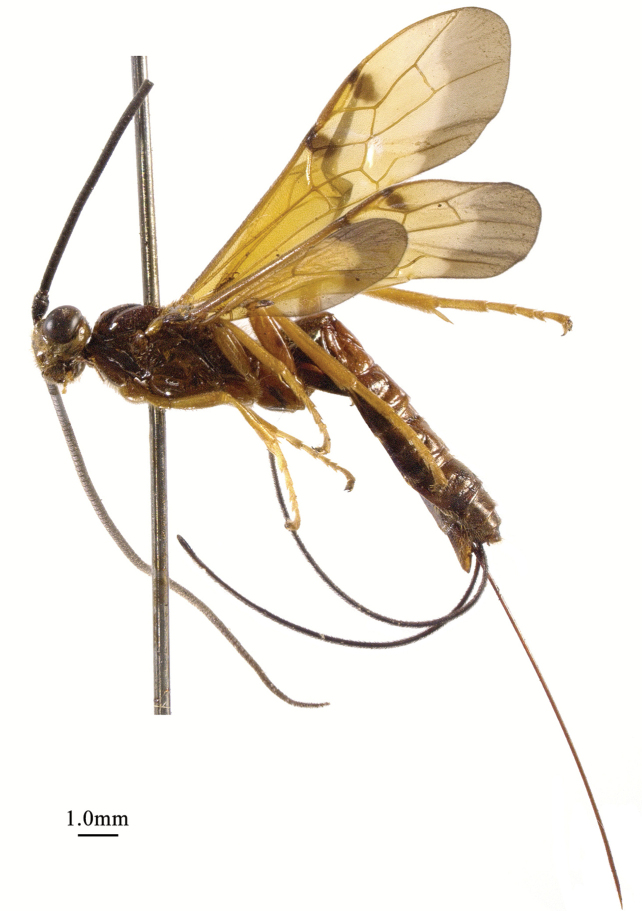
*Ischnobraconv-macula* (Cameron, 1899), ♀, habitus, lateral view.

**Figure 22.**
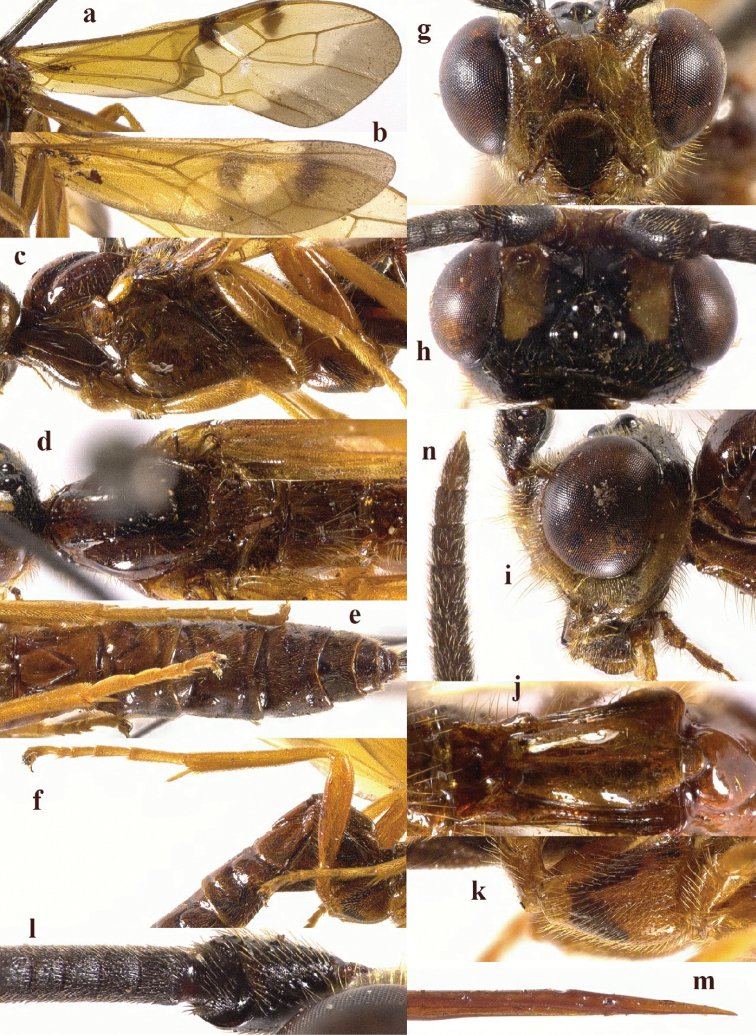
*Ischnobraconv-macula* (Cameron, 1899). ♀ **a** fore wing **b** hind wing **c** mesosoma, lateral view **d** mesosoma, dorsal view **e** metasoma, dorsal view **f** hind leg, lateral view **g** head, anterior view **h** head, dorsal view **i** head, lateral view **j** first metasomal tergite, dorsal view **k** hind femur, lateral view **l** scapus outer side, lateral view **m** apex of ovipositor, lateral view **n**. apex of antenna.

Figures 17 , 18 , 19 , 20 , 21 , 22

Ischnobracon Baltazar, 1963: 588; Quicke 1987: 117; Butcher and Quicke 2010: 2188. Type species: Ischnobraconbakeri Baltazar, 1963.

#### Diagnosis.

Body medium-sized to large; antenna longer than fore wing; terminal antennomere pointed, but not acute apically, median antennomeres distinctly wider than long; in lateral view scapus without double margin at inner side apically and strongly concave apico-laterally, ventrally ca. as long as or rarely slightly shorter than dorsally; eye large and glabrous, weakly emarginate; face weakly and sparsely punctate; clypeus moderately narrow and without dorsal carina, above clypeus with shallow triangular depression; malar suture moderately shallow; labio-maxillary complex normal, not or slightly elongate; frons weakly depressed, with some setae and a median groove; mesosoma smooth and shiny; notauli only impressed anteriorly; scutellar sulcus completely smooth; median area of metanotum relatively small; propodeum smooth and flattened; angle between veins 1-SR and C+SC+R of fore wing less than 40°; vein 1-SR+M of forewing usually straight, rarely slightly curved after arising from 1-M; forewing vein 3-CU1 usually more or less expanded posteriorly; second submarginal cell of fore wing distinctly expanded distally; forewing vein cu-a postfurcal or more or less interstitial; hind wing vein 2-SC+R usually interstitial or distinctly transverse (but longitudinal in *I.indiscretus*); hind wing with five to eight basal bristles; claws simple; fourth tarsal segment more or less protruding at inner side apically; metasoma in dorsal view relatively slender; T II with large rhombic medio-basal area and laterally depressed, resulting in a narrow medial part; T III often with large and raised antero-lateral areas; T III–V without deep oblique antero-lateral grooves; ovipositor with weak dorsal nodus and small ventral serrations subapically.

#### Biology.

Unknown.

#### Distribution.

Australasian; Oriental.

#### Note.

This genus is newly recorded from China.

### Key to Chinese species of the genus *Ischnobracon* Baltazar

**Table:** 

1	Black dorsal patch of head not reaching eye orbits and guttiform (Fig. 18h); antenna blackish brown basally (Fig. 18j), antennomeres lightened medially to distally, scape with a reversed U-shaped yellow patch dorsally (Fig. 18k) [China]	***I.guttatus* sp. nov.**
–	Black dorsal patch of head reaching eye orbits (Figs 20h, 22h); antenna entirely black, scape without yellow patch as above (Figs 20j, 22l, n)	**2**
2	Hind coxa with distinct piceous spot on outer side of coxa (Fig. 22k), and with or without a small dark patch on lower part of femur (Fig. 22f); base of hind wing with narrow glabrous areas on either side of vein cu-a (Fig. 22b) [China; India]	***I.v-macula***
–	Hind coxa and femur entirely yellow, without dark spots (Fig. 20f); base of hind wing largely with setae, only narrowly glabrous just distal to posterior half of vein cu-a (Fig. 20b) [China; Indonesia; Myanmar; Thailand]	***I.hannongbuai***

### Ischnobracon guttatus sp. nov.

Taxon classificationAnimaliaHymenopteraBraconidae

4E8B61C8-D865-58A1-8A6C-13DAE93CECFF

http://zoobank.org/7979DF17-3122-46D1-BFB2-F1E4F9F4922F

Figures 17 , 18

#### Material examined.

***Holotype***: ♀, China, Hainan Prov., Jianfengling, 29.IV.1983, Gu Maobin, No. IOZ(E)1964612 (IZCAS).

#### Diagnosis.

This new species is very similar to *I.baltazarae* Quicke & Butcher, 2010 [Philippines], but can be separated from the latter by the following characters: scape blackish brown with a reversed U-shaped yellow spot dorsally (yellow with black lateral streak in *I.baltazarae*); second submarginal cell of fore wing relatively short, vein 2-M 2.8× as long as vein 2-SR (3.45 × vein 2-SR); fore wing vein cu-a distinctly curved and postfurcal (straight and interstitial); base of hind wing with rather narrow glabrous areas on either side of vein cu-a (hind wing subbasal cell glabrous on posterior half and with large glabrous area distal to vein cu-a); hind femur yellow, without black mark (hind femur black ventro-distally).

#### Description.

Holotype, ♀, length of body 14.8 mm, of fore wing 12.0 mm, of ovipositor sheath 9.0 mm.

***Head*.** Antenna incomplete, with 88 antennomeres remaining; median antennomeres 1.6× wider than their length; third antennomere 1.1 and 1.2× longer than fourth and fifth respectively, the latter being 1.4× wider than its length; scapus 1.5× longer than its apical width (Fig. 18j); tentorio-ocular distance: inter-tentorial distance: distance between clypeus and antennal sockets = 7: 9: 14; shortest distance between eyes: head width: eye height = 18: 35: 17; shortest distance between posterior ocelli: shortest distance between posterior ocellus and eye: width of head behind eyes (occiput) = 4: 9: 34; occiput with sparse setae medially, and with dense long setae laterally (Fig. 18h).

***Mesosoma*.** Length of mesosoma twice its height (Fig. 18c); notauli only impressed on anterior third of mesoscutum (Fig. 18d).

***Wings*.** Fore wing (Fig. 18a): r: SR1: 3-SR = 8: 17: 19; 1-SR+M more or less straight; 2-SR: 3-SR: r-m = 8: 17: 8; 2-M 2.8× longer than 2-SR; cu-a strongly curved and postfurcal. Hind wing (Fig. 18b): base with a rather narrow glabrous area distal to cu-a; 2-SC+R distinctly transverse; 1r-m 1.9× longer than SC+R1.

***Legs*.** Length of fore femur: tibia: tarsus = 24: 27: 38; fore basitarsus 4.2× longer than its maximum width; fore tarsus ventro-apically with rather dense and long setae; length of hind femur: tibia: basitarsus = 33: 48: 16; length of femur, tibia and basitarsus of hind leg 3.9, 8.0 and 4.0× their maximum width, respectively (Fig. 18f).

***Metasoma*.** Metasomal tergites smooth (Fig. 18e); length of T I 1.6× its apical width, raised median area not depressed medially (Fig. 18e); median length of T II 1.1× its apical width; antero-lateral areas of T III large, apical width of T III 1.2× its median length (Fig. 18e); tergites with dense and long setae especially posteriorly except for the raised areas (Fig. 18e); ovipositor sheath 0.7× as long as fore wing.

***Colour*.** Largely yellow (Fig. 17); eye, mandible apically, metanotum and propodeum blackish brown (Fig. 18d, g); antenna blackish brown, becoming yellow towards apex (Fig. 17); scapus with a reverse yellow U-shaped spot dorsally (Fig. 18k); around stemmaticum with a drop-shaped black spot (Fig. 18h); ovipositor sheath black (Fig. 17); wing membrane largely yellow but smoky grey apically; stigmal spot brown; marginal cell with a small brown spot anteriorly; pterostigma yellow; veins largely yellow (Fig. 18a, b).

#### Biology.

Unknown.

#### Distribution.

China (Hainan).

#### Etymology.

Named after the surrounded area of stemmaticum with a drop-shaped black spot: *guttatus* is Latin for drop-shaped.

### Ischnobracon hannongbuai

Taxon classificationAnimaliaHymenopteraBraconidae

Quicke & Butcher, 2010

8E8D6C52-3A1B-5119-9FF1-F12AC3BCEAAD

Figures 19 , 20

Ischnobracon hannongbuai Quicke & Butcher in Butcher and Quicke 2010: 2201.

#### Material examined.

1♀, China, Yunnan Prov., Xishuangbanna, Damenglong, 650 m, 11.VII.1958, Hong Chunpei, No. IOZ(E)1964525 (IZCAS); 1♀, China, Yunnan Prov., Xiaomengyang, 900 m, 5.V.1957, Pu Fuji, No. IOZ(E) 1964596 (IZCAS); 1♀, China, Yunnan Prov., Yiwubanna, MengLun, 650 m, 3.VIII.1959, Li Suofu, No. IOZ(E) 1964526 (IZCAS); 1♀, China, Yunnan Prov., Xishuangbanna, Meng’a, 1050 m, 16.X.1958, Chen Zhizi, No. IOZ(E) 1964628 (IZCAS).

#### Biology.

Unknown.

#### Distribution.

China (Yunnan); Indonesia; Myanmar; Thailand.

#### Note.

This species is new to China.

### Ischnobracon v-macula

Taxon classificationAnimaliaHymenopteraBraconidae

(Cameron, 1899)

219D3745-E7A7-597F-8B72-945ECDE75844

Figures 21 , 22

Bracon v-macula Cameron, 1899: 62.
Elphea v-macula (Cameron): Dover, 1925: 39.
Stenobracon v-macula (Cameron): Fahringer, 1928: 28.
Ischnobracon v-macula (Cameron): Baltazar, 1972: 265; Butcher et Quicke 2010: 2208.
Bracon orientalis Cameron, 1899: 63.
Ischnobracon orientalis (Cameron): Baltazar, 1972: 265 (synonymy, lectotype designation); Shenefelt 1978: 1690.

#### Material examined.

1♀, China, Yunnan Prov., Xishuangbanna, Xiaomengyang, 850 m, 22.X.1957, Wang Shuyong, No. IOZ(E)1964524 (IZCAS); 1♀, China, Yunnan Prov., Xishuangbanna, Mengla, 620–650 m, 10.VI.1959, Li Suofu, No. IOZ(E) 1964523 (IZCAS); 1♀, China, Yunnan Prov., Cheli, 620 m, 18.IV.1957, Zang Lingchao, No. IOZ(E) 1964537 (IZCAS).

#### Biology.

Unknown.

#### Distribution.

China (Yunnan); India.

#### Note.

This species is newly recorded from China.

### Genus Monilobracon

Taxon classificationAnimaliaHymenopteraBraconidae

Quicke, 1984

573F2845-8AE0-52DA-88F3-B60D7A7E2844

**Figure 23.**
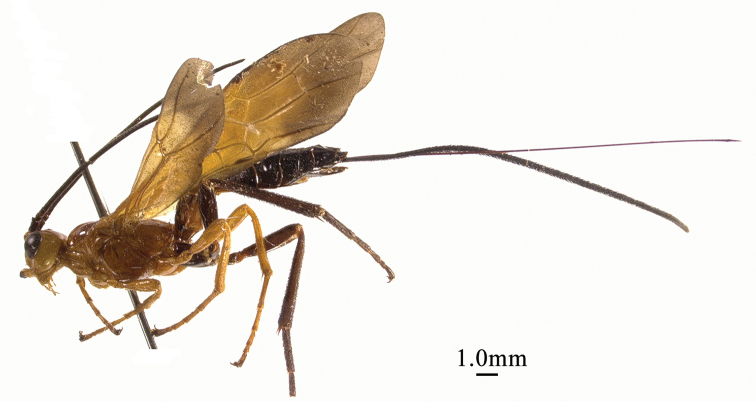
*Monilobraconlongitudinalis* sp. nov., ♀, holotype, habitus, lateral view.

**Figure 24.**
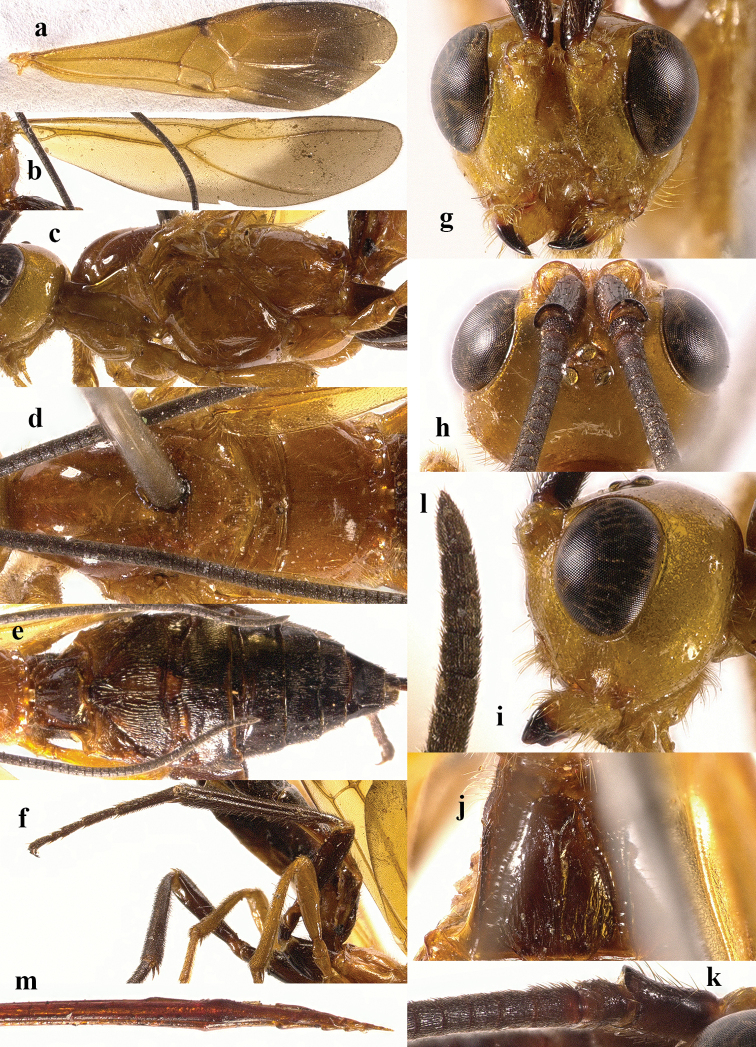
*Monilobraconlongitudinalis* sp. nov., ♀, holotype **a** fore wing **b** hind wing **c** mesosoma, lateral view **d** mesosoma, dorsal view **e** metasoma, dorsal view **f** hind leg, lateral view **g** head, anterior view **h** head, dorsal view **i** head, lateral view **j** first metasomal tergite, dorsal view **k** scapus outer side, lateral view **l** apex of antenna **m** apex of ovipositor, lateral view.

**Figure 25.**
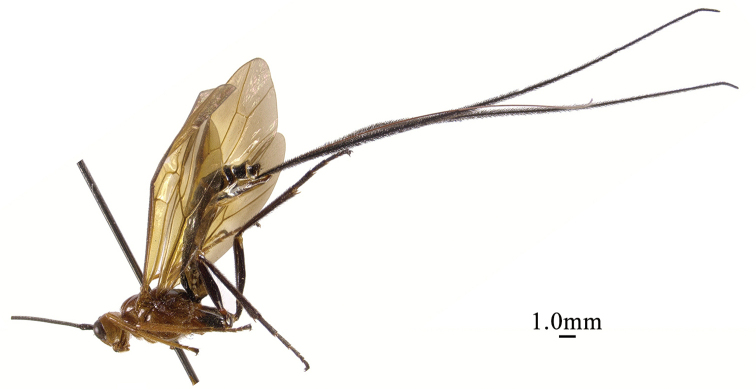
*Monilobraconmarginatus* sp. nov., ♀, holotype, habitus, lateral view.

**Figure 26.**
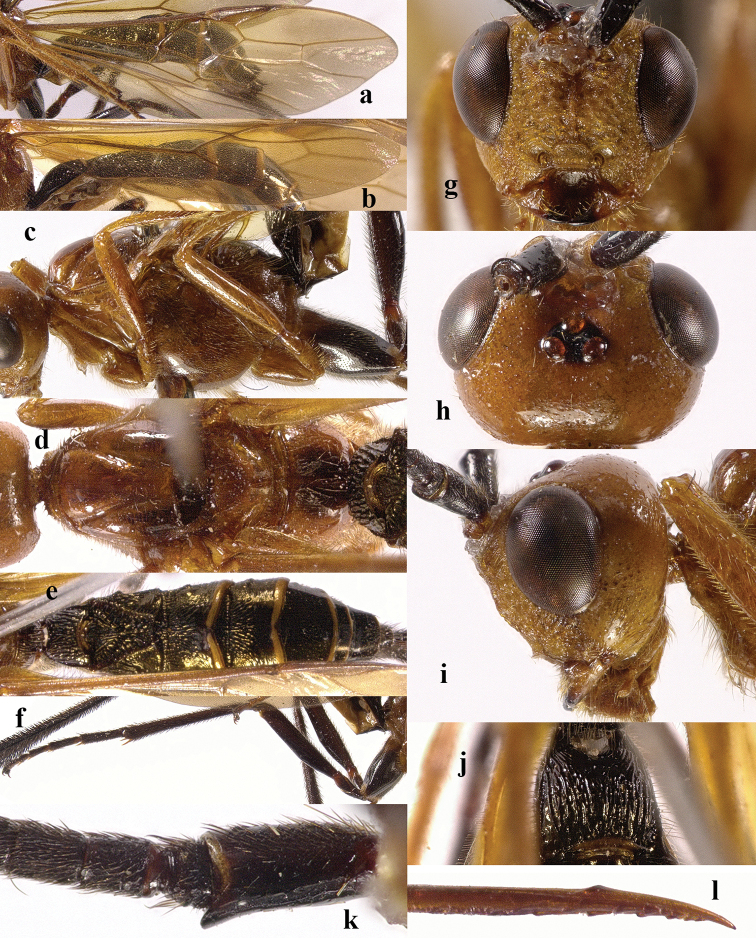
*Monilobraconmarginatus* sp. nov., ♀, holotype **a** fore wing **b** hind wing **c** mesosoma, lateral view **d** mesosoma, dorsal view **e** metasoma, dorsal view **f** hind leg, lateral view **g** head, anterior view **h** head, dorsal view **i** head, lateral view **j** first metasomal tergite, dorsal view **k** scapus outer side, lateral view **l** apex of ovipositor, lateral view.

Figures 23 , 24 , 25 , 26

Monilobracon Quicke, 1984b: 39, 1987: 120; Papp 1998: 234. Type species: Monilobraconspeciosus Quicke, 1984 (monobasic and original designation).

#### Diagnosis.

Body medium-sized to large; terminal antennomere slightly acute apically; scapus often with a strong secondary edge on its inner side apically, distinctly removed from pedicellus and with more or less oblique and usually angulate outer side basally, and pedicellus more or less petiolate basally in dorsal view or pedicellus and scapus both strongly compressed; outer side of scapus distinctly angulate and oblique subbasally, but sometimes rounded; in lateral view scapus often ventrally longer than dorsally; lateral of antennal sockets usually with deep depression (often as oblique groove, but sometimes weak); eye glabrous, not or weakly emarginate; face medio-dorsally with a reversed Y-shaped impression or with a narrow groove, but sometimes obsolescent; clypeus moderately narrow, partly or completely flattened and without dorsal carina; malar suture moderately developed; labio-maxillary complex normal, not elongate; frons often strongly depressed, with a developed median groove; mesosoma largely smooth and shiny; notauli at least present anteriorly, sometimes complete; scutellar sulcus crenulate; metanotum strongly convex medially; propodeum largely smooth, sometimes with a short medio-longitudinal groove anteriorly, and often with crenulae posteriorly; angle between veins 1-SR and C+SC+R of fore wing more than 60°; fore wing vein 1-SR+M variable, more or less straight, or weakly curved to distinctly angled after arising from vein 1-M; hind wing vein C+SC+R with only one basal bristle; claws simple; metasomal tergites usually largely strongly sculptured, rarely smooth; T I with a medio-longitudinal carina posteriorly, dorsolateral carinae present; T II usually with a weakly to strongly raised medio-basal triangular area connected to medio-longitudinal carina apically, but absent near posterior margin of T II; T II–IV partly coarsely striate-rugose; hypopygium rather acute apically; ovipositor with dorsal nodus and ventral serrations subapically, often longer than body.

#### Biology.

Unknown.

#### Distribution.

Afrotropical; Australasian; Oriental; Palaearctic.

#### Note.

This genus is newly recorded from China.

### Key to Chinese species of the genus *Monilobracon* Quicke

**Table:** 

1	Stemmaticum yellow (Fig. 24h); wing membrane with a small stigmal spot below parastigma (Fig. 24a); medio-basal area of T II relatively small and sub-lateral areas large (Fig. 24e); T IV smooth (Fig. 24e); metasomal tergites blackish brown, but sub-lateral areas of T II partly yellowish (Fig. 24e); ovipositor sheath 1.1–1.3× longer than fore wing (Fig. 23) [China]	***M.longitudinalis* sp. nov.**
–	Stemmaticum blackish brown (Fig. 26h); wing membrane without a stigmal spot (Fig. 26a); medio-basal area of T II relatively large, without sub-lateral areas (Fig. 26e); basal half of T IV with striae (Fig. 26e); metasomal tergites blackish brown, but posterior margins of T III–VII whitish yellow (Fig. 26e); ovipositor sheath 2.0× longer than fore wing (Fig. 25) [China]	***M.marginatus* sp. nov.**

### Monilobracon longitudinalis sp. nov.

Taxon classificationAnimaliaHymenopteraBraconidae

D1379F0A-A56B-51EC-936A-0EDC55F8F385

http://zoobank.org/FF922A5B-ED10-4BE8-851B-5541E3B33C9A

Figures 23 , 24

#### Material examined.

***Holotype***: ♀, China, Yunnan Prov., Xishuangbanna, Xiaomengyang, 850 m, 20.X.1958, Pu Fuji, No. IOZ(E)1964555 (IZCAS). Paratypes. 2♀, same data as holotype, but No. IOZ(E)1964575 and IOZ(E)1964576 (IZCAS). 1♀, id., but 19.VIII.1958, Zhang Yiran, No. IOZ(E)1964557 (IZCAS).

#### Diagnosis.

This new species is very similar to *Monilobraconquadriceps* (Smith, 1858) [Malaysia], but can be separated from the latter by the following characters: hind coxae blackish brown (reddish brown in *M.quadriceps*); wing membrane yellow, greyish brown apically, without subhyaline spot (with few subhyaline spots); T I blackish brown laterally (lateral areas yellow); fore wing vein 1-SR+M more or less straight (weakly curved).

#### Description.

Holotype, ♀, length of body 15.2 mm, of fore wing 14.8 mm, of ovipositor sheath 16.6 mm.

***Head*.** Antenna with 64 antennomeres; apical antennomere pointed and slightly acute, 2.1× longer than its maximum width (Fig. 24l); penultimate antennomere 1.2× longer than its maximum width, and 0.6× as long as apical antennomere; median antennomeres 0.9× longer than wide; third antennomere 1.3 and 1.4× longer than fourth and fifth, respectively, the latter 1.2× longer than wide; length of maxillary palp 0.8× height of head; malar suture with sparse short setae, and with fine punctures (Fig. 24i); clypeus height: inter-tentorial distance: tentorio-ocular distance = 7: 10: 8; clypeus with sparse long setae; eye weakly emarginated (Fig. 24g); face largely glabrous except for a few short setae, and with some sparse punctures (Fig. 24g); eye height: shortest distance between eyes: head width = 4: 5: 10; frons largely smooth, strongly concave behind antennal sockets, with a developed median groove (Fig. 24h); vertex largely smooth except for a few weak punctures, with some sparse short setae; minimum distance between posterior ocelli: minimum diameter of elliptical posterior ocellus: minimum distance between posterior ocellus and eye = 5: 6: 18; length of malar space 1.0× basal width of mandible; temples largely glabrous except for a few short setae, and subparallel behind eyes (Fig. 24h); in dorsal view length of eye 1.4× temple.

***Mesosoma*.** Length of mesosoma 1.9× its height (Fig. 24c); notauli impressed anterior half, shallow posteriorly (Fig. 24d); mesoscutum smooth, with sparse short setae (Fig. 24d); scutellar sulcus wide, moderately deep, and with crenulae (Fig. 24d); scutellum with dense short setae posteriorly; metanotum strongly convex medially (Fig. 24d); propodeum smooth, without medio-longitudinal groove anteriorly, with a few short crenulae posteriorly, with sparse setae medially, and with dense long setae laterally (Fig. 24d).

***Wings*.** Fore wing (Fig. 24a): SR1: 3-SR: r = 38: 35: 7; 1-SR+M more or less straight, and 1.1× longer than 1-M; 2-SR: 3-SR: r-m = 16: 35: 15; m-cu straight, and 4.0× longer than 2-SR+M; cu-a slightly postfurcal. Hind wing (Fig. 24b): 1r-m transverse; SC+R1: 2-SC+R: 1r-m = 28: 4: 19.

***Legs*.** Length of fore femur: tibia: tarsus = 5: 6: 9; length of hind femur: tibia: basitarsus = 38: 69: 21; length of femur, tibia, and basitarsus of hind leg 3.8, 9.9, and 5.3× their maximum widths, respectively (Fig. 24f).

***Metasoma*.** Length of T I 1.3× its apical width, median area convex and with a medio-longitudinal carina, smooth anteriorly, and longitudinally rugose posteriorly (Fig. 24j); lateral grooves of T I sparsely crenulate (Fig. 24j); T II longitudinally rugose including medio-basal area, but antero-lateral areas smooth posteriorly (Fig. 24e); triangular medio-basal area of T II moderately large, attached to medio-longitudinal carina apically but absent near posterior margin of T II; antero-lateral areas of T II developed, longitudinally rugose anteriorly and smooth posteriorly, anterior grooves weakly and sparsely crenulate (Fig. 24e); second suture deep and crenulate, wide and curved medially, narrow laterally (Fig. 24e); T III largely longitudinally rugose but smooth posteriorly, antero-lateral areas weakly rugose; T III–V with antero-lateral areas, and crenulate transverse subposterior groove (Fig. 24e); T IV–VII smooth, and with dense long setae posteriorly; hypopygium acute apically, reaching level of apex of metasoma; ovipositor sheath 1.1× as long as fore wing.

*Colour*. Head and mesosoma largely yellow (Fig. 23); antenna, eye, and mandible apically black (Fig. 24g); fore and middle legs (except for tarsus apically and claws black) yellow, hind leg black (Fig. 24f); metasoma (except T I and T II dark brown) and ovipositor sheath black (Figs 23, 24e); wing membrane yellow, but black brown apically (hind wing including posterior margin medially), stigmal spot black brown, first subdiscal cell of fore wing with a blackish brown spot medio-posteriorly, pterostigma yellow but apically blackish brown, basal veins yellow and apical veins dark brown (Fig. 24a, b).

*Variation*. Length of body of female 14.9–15.9 mm, of fore wing of female 14.6–15.6 mm, and of ovipositor sheath 16.6–21.0 mm; antenna of female with 64–66 antennomeres; median antennomeres 0.9–1.2× longer than wide; face sometimes with dense short setae; length of mesosoma 1.7–1.9× its height; fore wing vein SR1 0.9–1.1× longer than vein 3-SR; ovipositor sheath 1.1–1.3× as long as fore wing; second-ninth antennomeres sometimes infuscate.

#### Biology.

Unknown.

#### Distribution.

China (Yunnan).

#### Etymology.

Named after the largely longitudinally rugose T I–III: *longitudinalis* is Latin for longitudinal.

### Monilobracon marginatus sp. nov.

Taxon classificationAnimaliaHymenopteraBraconidae

9BE94752-62C6-58F0-8B66-A09810C170D7

http://zoobank.org/9B34C73B-9D24-42ED-BE4D-75EFA497777C

Figures 25 , 26

#### Material examined.

***Holotype***: ♀, China, Yunnan Prov., Xishuangbanna, Xiaomengyang, 850 m, 23.VI.1957, Zang Lingchao, No. IOZ(E)1964556 (IZCAS).

#### Diagnosis.

This new species is very similar to *Monilobraconlongitudinalis* sp. nov., but can be separated from the latter by the following characters: stemmaticum blackish brown (yellow in *M.longitudinalis*); fore wing without a stigmal spot (with a blackish brown stigmal spot); medio-basal area of T II relatively large and without sub-lateral areas (medio-basal are relatively small, and with large sublateral areas); basal half of T IV with striae (smooth); metasomal tergites blackish brown, but posterior margins of T III–VII whitish yellow (metasomal tergites blackish brown, but sublateral areas of T II partly yellowish); ovipositor sheath 2.0× longer than fore wing (1.1–1.3×).

#### Description.

Holotype, ♀, length of body 13.6 mm, of fore wing 12.7 mm, of ovipositor sheath 25.0 mm.

***Head*.** Antenna incomplete, with 19 antennomeres remaining; median antennomeres as long as wide; third antennomere 1.2 and 1.3× longer than fourth and fifth, respectively, the latter 1.2× longer than wide; maxillary palp incomplete; malar suture with sparse short setae, and finely sculptured (Fig. 26i); clypeus height: inter-tentorial distance: tentorio-ocular distance = 7: 9: 8; clypeus with sparse short setae; eye weakly emarginate (Fig. 26g); face coarsely sculptured (Fig. 26g); eye height: shortest distance between eyes: head width = 19: 25: 48; frons largely smooth, strongly concave behind antennal sockets, with a distinct median groove (Fig. 26h); vertex largely smooth except for a few weak punctures, with some sparse short setae; minimum distance between posterior ocelli: minimum diameter of elliptical posterior ocellus: minimum distance between posterior ocellus and eye = 5: 7: 16; length of malar space equal to basal width of mandible; temples largely glabrous except for a few short setae, and weakly narrowed behind eyes (Fig. 28h); in dorsal view length of eye 1.5× temple.

***Mesosoma*.** Length of mesosoma 1.5× its height (Fig. 26c); notauli impressed in anterior half of mesoscutum, shallow posteriorly (Fig. 26d); mesoscutum smooth, with sparse short setae (Fig. 26d); scutellar sulcus rather wide, moderately deep, and with crenulae (Fig. 26d); scutellum with dense short setae posteriorly; metanotum strongly convex medially (Fig. 26d); propodeum smooth, with short medio-longitudinal groove anteriorly, and with few short crenulae posteriorly, with sparse setae medially, and with dense long setae laterally (Fig. 26d).

***Wings*.** Fore wing (Fig. 26a): SR1: 3-SR: r = 36: 26: 7; 1-SR+M weakly and evenly curved, and 1.6× longer than 1-M; 2-SR: 3-SR: r-m = 11: 26: 7; m-cu straight, and 2.0× longer than 2-SR+M; cu-a interstitial. Hind wing (Fig. 26b): 1r-m longitudinal; SC+R1: 2-SC+R: 1r-m = 20: 4: 19.

***Legs*.** Length of fore femur: tibia: tarsus = 29: 32: 46; length of hind femur: tibia: basitarsus = 40: 56: 17; length of femur, tibia and basitarsus of hind leg 5.3, 11.2 and 5.7× their maximum width, respectively (Fig. 26f).

***Metasoma*.** Length of T I 1.2× its apical width, median area convex and strongly longitudinally rugose, with a medio-longitudinal carina (Fig. 26j); lateral grooves of T I crenulate (Fig. 26j); T II longitudinally rugose including medio-basal area, but smooth posterior-laterally (Fig. 26e); triangular medio-basal area of T II very large, attached with medio-longitudinal carina apically absent near posterior margin of T II; antero-lateral areas of T II absent, anterior grooves moderately impressed and crenulate (Fig. 26e); second suture deep and crenulate, wide and curved medially, narrow laterally (Fig. 26e); T III largely longitudinally rugose except for smooth posteriorly, antero-lateral areas weak and smooth posteriorly; T IV longitudinally rugose medio-anteriorly, and with weak antero-lateral areas; T V–VII smooth and antero-lateral areas absent (Fig. 26e); hypopygium acute apically, reaching level of apex of metasoma; ovipositor sheath 2.0× as long as fore wing.

***Colour*.** Head and mesosoma largely yellow (Fig. 25); antenna, eye, stemmaticum and mandible apically, black (Fig. 28g, h); fore and middle legs (but tarsus apically and claws black) yellow, hind leg black (coxa infuscate basally) (Fig. 26f); metasoma largely black, posterior margins of T III–VII whitish yellow (Fig. 26e); ovipositor sheath black (Fig. 25); wing membrane yellow, but grey brown apically and hind wing also medio-posteriorly), pterostigma (but apically blackish brown) and veins yellow (Fig. 26a, b).

#### Biology.

Unknown.

#### Distribution.

China (Yunnan).

#### Etymology.

Named after the whitish yellow posterior margins of the T III–VII: *marginatus* is Latin for margin.

### Parallobracon gen. nov.

Taxon classificationAnimaliaHymenopteraBraconidae

B18B704B-F803-5D49-9A4D-D0D0CAE0973C

http://zoobank.org/3A4A4BF7-6E4A-444E-AD8B-9A7F1D032B3B

Figures 27 , 28

#### Type species.

*Parallobraconprolatus* sp. nov.

#### Diagnosis.

Antennomeres (except scape and pedicel) square; scapus rather slender, and in lateral view without double margin at inner side apically and apex strongly protruding ventrally; eye glabrous, not emarginated; face flattened in lateral view; clypeus flat and with distinct dorsal carina; malar suture present but weak, with dense short setae; labio-maxillary complex normal, not elongate; frons with strong median groove, largely smooth; notauli quite shallow, and impressed anteriorly on disc; scutellar sulcus comparatively narrow, and sparsely crenulate; metanotum convex medially, but without median carina anteriorly; propodeum smooth, and without medio-longitudinal carina or groove; first discal cell of fore wing nearly parallel-sided and ca. 3.0× longer than vein m-cu; vein 1-SR+M of fore wing straight; vein 1r-m of hind wing ca. 5.0× longer than vein 2-SC+R; vein 1-SR of fore wing distinctly oblique and pointing basad of vein cu-a, angle with vein C+SC+R ca. 55°; fore wing vein cu-a weakly postfurcal; vein 2-SC+R of hind wing transverse, distinctly shorter than vein 1r-m; hind wing with densely setae basally; claws simple; legs more or less with sparsely setae; in dorsal view, metasoma ovoid; median area of T I developed and coarsely sculptured posteriorly; medio-basal area of T II wide subbasally and acute apically, latero-basal areas triangular and medium-sized and posterior half of tergite with pair of diverging depressions; second suture deep and wide, crenulate, narrowed and curved upward laterally, weakly curved medially; T III with developed antero-lateral areas and posterior margin of tergite sinuate, and 3.8× wider than its median length (excluding its basal groove); T III–V with crenulate transverse subposterior groove; T III–VII largely smooth; ovipositor with minute ventral teeth and without dorsal nodus.

#### Distribution.

Oriental (China).

#### Etymology.

Named after the nearly parallel-sided first discal cell of the fore wing (*parallelus* is Latin for “sides of equal distance”). Gender: masculine.

#### Note.

This new genus will run in existing keys to *Cyanopterus* Haliday, 1835 (e.g., Belokobylskij 2000), but can be separated from the latter by the following characters: first discal cell of fore wing nearly parallel-sided and elongate (first discal cell of fore wing widened basally in *Cyanopterus*); vein 1r-m of hind wing quite long and ca. 5.0× longer than vein 2-SC+R (vein 1r-m of hind wing at most ca. 2.0× longer than vein 2-SC+R in *Cyanopterus*); vein 1-SR of fore wing distinctly oblique and pointing basad of vein cu-a (less oblique and pointing to vein cu-a in *Cyanopterus*); second submarginal cell of fore wing widened distally (second submarginal cell of fore wing parallel-sided in *Cyanopterus*); apex of scapus strongly protruding ventrally (apex of scapus slightly protruding ventrally in *Cyanopterus*); clypeus with distinct dorsal carina (clypeus usually without dorsal carina in *Cyanopterus*).

### Parallobracon prolatus sp. nov.

Taxon classificationAnimaliaHymenopteraBraconidae

BE215305-9391-53B1-A5E2-6EE2B84FE25C

http://zoobank.org/2E01D4E3-D4CE-48D3-82E7-3A22D5E957F1

Figures 27 , 28

#### Material examined.

***Holotype***: ♀, China, Zhejiang Prov., Hangzhou, 26.VI.1935, Zhu Ruzuo (ZJUH). Paratype. 1♀1♂, China, Zhejiang Prov., Hangzhou, 17.VI.1934, Zhu Ruzuo (ZJUH).

#### Diagnosis.

T II of the new species is similar to that of *Cyanopterusoriens* Belokobylskij, 2000, from Far East Russia (e.g., size and shape of medio-basal area and with pair of diverging depressions), but can be separated by having first discal cell of fore wing parallel-sided and vein 1-SR+M straight (widened basally and vein 1-SR+M weakly bent in *C.oriens*), vein 1r-m of hind wing ca. 5 × longer than vein 2-SC+R (ca. equal in *C.oriens*), vein 1-SR of fore wing distinctly oblique and pointing basad of vein cu-a (less oblique and pointing to vein cu-a in *C.oriens*), second submarginal cell of fore wing widened distally (parallel-sided in *C.oriens*) and apex of scapus strongly protruding ventrally (slightly protruding in *C.oriens*).

#### Description.

Holotype, ♀, length of body 7.6 mm, of fore wing 7.7 mm, of ovipositor sheath 4.9 mm.

**Figure 27.**
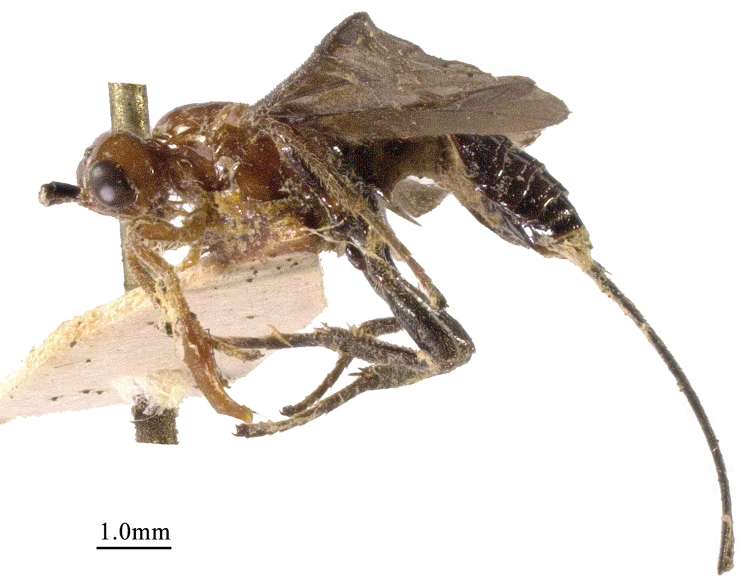
*Parallobraconprolatus* gen. et sp. nov., ♀, holotype, habitus, lateral view.

**Figure 28.**
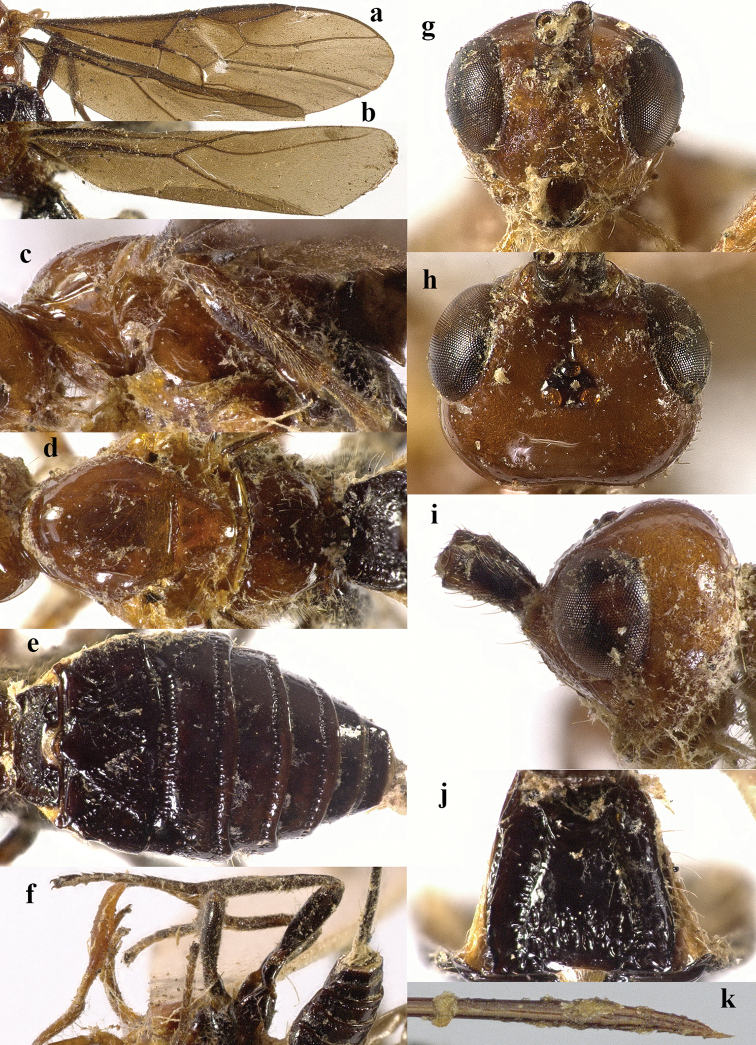
*Parallobraconprolatus* gen. et sp. nov., ♀, holotype (**a–j**), paratype (**k**) **a** fore wing **b** hind wing **c** mesosoma, lateral view **d** mesosoma, dorsal view **e** metasoma, dorsal view **f** hind leg, lateral view **g** head, anterior view **h** head, dorsal view **i** head, lateral view **j** first metasomal tergite, dorsal view **k** apex of ovipositor, lateral view.

***Head*.** Antenna incomplete, only remaining with scapus and pedicellus; malar suture with dense short setae (Fig. 28i); clypeus height: inter-tentorial distance: tentorio-ocular distance = 3: 6: 7; clypeus with sparse short setae; eye weakly emarginated (Fig. 28g); face weakly convex medially and weakly granulate, with some sparse punctures laterally (Fig. 28g); eye height: shortest distance between eyes: head width = 17: 20: 40; frons largely smooth, weakly concave behind antennal sockets, with a strong median groove (Fig. 28h); vertex smooth, but with some sparse short setae; minimum distance between posterior ocelli: minimum diameter of elliptical posterior ocellus: minimum distance between posterior ocellus and eye = 1: 1: 2; temples largely glabrous except for a few short setae, and subparallel behind eyes (Fig. 28h).

***Mesosoma*.** Length of mesosoma 1.9× its height (Fig. 28c); notauli impressed anteriorly half (Fig. 28d); mesoscutum smooth, with a few sparse setae (Fig. 28d); scutellar sulcus narrow, moderately deep, and with crenulae (Fig. 28d); scutellum with sparse short setae posteriorly; metanotum strongly convex medially (Fig. 28d); propodeum smooth, without longitudinal carinae or groove, with sparse setae medially, and with dense long setae laterally (Fig. 28d).

***Wings*.** Fore wing (Fig. 28a): SR1: 3-SR: r = 21: 13: 5; 1-SR+M more or less straight, and 1.9× longer than 1-M; 2-SR: 3-SR: r-m = 14: 26: 13; angle between 1-SR and C+SC+R ca. 75°; m-cu straight; 2-SR+M rather short; cu-a slightly postfurcal. Hind wing (Fig. 28b): SC+R1: 2-SC+R: 1r-m = 17: 3: 13.

***Legs*.** Length of fore femur: tibia: tarsus = 27: 31: 45; length of hind femur: tibia: basitarsus = 37: 53: 21; length of femur, tibia and basitarsus of hind leg 3.5, 6.6 and 5.3× their maximum width, respectively (Fig. 28f); hind tibial spurs 0.35 and 0.40 × as long as hind basitarsus.

***Metasoma*.** Length of T I 1.1× its apical width, median area convex, anteriorly half smooth and posteriorly half coarsely sculptured (Fig. 28j); lateral grooves of T I sparsely crenulate (Fig. 28j); T II largely smooth except medially (Fig. 28e); triangular medio-basal area of T II large and smooth, with a few short oblique carinae connected laterally, and acute apically, but not attached with medio-longitudinal carina; antero-lateral areas of T II developed and smooth, anterior grooves moderately wide and sparsely crenulate (Fig. 28e); second suture deep and crenulate, wide and weakly curved medially, narrow laterally (Fig. 28e); T III with antero-lateral areas; T III–V with crenulate transverse subposterior groove (Fig. 28e); T III–VII largely smooth, and with some spare short setae posteriorly; hypopygium acute apically, not reaching level of apex of metasoma; ovipositor sheath 0.6× as long as fore wing.

***Colour*.** Head and mesosoma largely reddish yellow (Fig. 27); antenna, eye, mandible apically, stemmaticum, propodeum medially and posteriorly, blackish brown (Fig. 28d, g, h); fore legs (but claws dark brown) reddish yellow, middle and hind legs dark brown (Fig. 28f); metasoma and ovipositor sheath black brown (Figs 27, 28e); wing membrane greyish brown, pterostigma and veins dark brown (Fig. 28a, b).

***Variation*.** Length of body of female 7.6–7.7 mm, of fore wing of female 7.7 mm, and of ovipositor sheath 4.2–4.9 mm; antenna of paratype female incomplete, with 30 antennomeres remaining; third antennomere 1.7× longer than its maximum width, 1.2 and 1.3× longer than fourth and fifth, respectively, the latter 1.3× longer than wide; ovipositor sheath 0.5–0.6× as long as fore wing.

***Male*.** Length of body of male 7.8 mm, of fore wing of male 7.9 mm; antenna of male with 44 antennomeres; Length of mesosoma 1.7× its height; fore wing vein cu-a interstitial; length of T I 1.0× its apical width; other characters as in female.

#### Biology.

Unknown.

#### Distribution.

China (Zhejiang).

#### Etymology.

Named after the long and slender scapus which strong elongated ventrally: *prolatus* is Latin for elongated.

### Genus Pseudospinaria

Taxon classificationAnimaliaHymenopteraBraconidae

Enderlein, 1905

685FBB24-7097-50A9-A91E-5FADAB09504B

Figures 29 , 30

Pseudospinaria Enderlein, 1905: 229; Quicke 1987: 127. Type species: Spinariaattenuata Westwood, 1882 (Monobasic and original designation).

#### Diagnosis.

Body medium-sized to large; terminal antennomere slender, strongly acute apically; scapus in lateral view basally rounded and at inner side usually with narrow apical ledge, concave apico-laterally, ventrally shorter than dorsally; eye glabrous, weakly emarginate; face coarsely sculptured; clypeus narrow and without dorsal carina; malar suture moderately developed, often with long and dense setae; labio-maxillary complex normal, not elongate; frons broadly impressed, with some setae and median groove; vertex and temples transversely rugose; notauli complete; mesoscutum with deep punctures near the notauli; metanotum with median carina protruding over propodeum posteriorly, and with a complete mid-longitudinal carina; propodeum coarsely punctate; angle between veins 1-SR and C+SC+R of fore wing ca. 60°; vein r of fore wing long, (nearly) ca. as long as vein 2-SR; fore wing vein 2-SR sinuate; vein 1-R1 of fore wing distinctly longer than pterostigma; fore wing vein cu-a slightly postfurcal; second submarginal cell of fore wing slender, but somewhat widened apically; tarsal claws with an additional tooth; basal lobes of claws rounded; T I without a media-longitudinal carina; T II with wide triangular medio-basal area, area reaching apex of tergite or nearly so; T IV–VI with lateral teeth; T VII of female with medio-apical tooth (absent in male); ovipositor with dorsal nodus and ventral serrations subapically; ovipositor sheath 0.1–0.3× as long as fore wing.

#### Biology.

Unknown.

#### Distribution.

Oriental.

#### Note.

This genus is newly recorded from China. Of the two described species, only one species is recorded from China.

**Figure 29.**
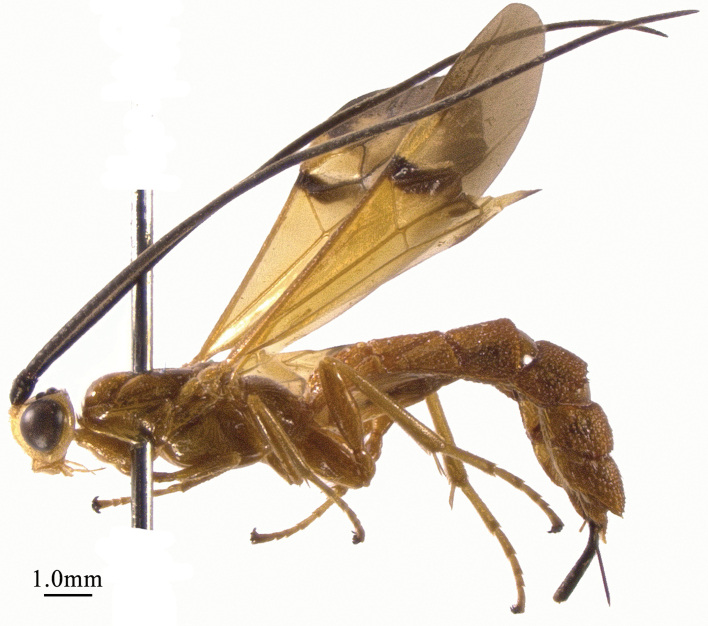
*Pseudospinariaattenuata* (Westwood, 1882), ♀, habitus, lateral view.

**Figure 30.**
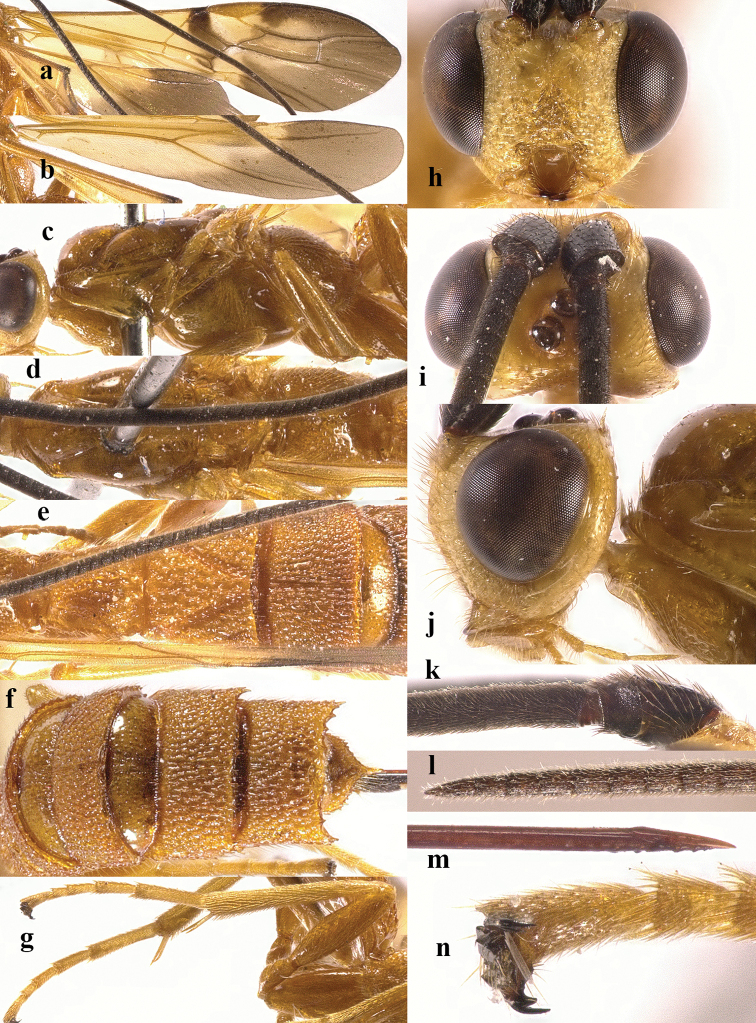
*Pseudospinariaattenuata* (Westwood, 1882). ♀ **a** fore wing **b** hind wing **c** mesosoma, lateral view **d** mesosoma, dorsal view **e** first to third metasomal tergites, dorsal view **f** fourth to seventh metasomal tergites, dorsal view **g** hind leg, lateral view **h** head, anterior view **i** head, dorsal view **j** head, lateral view **k** scapus outer side, lateral view **l** apex of antenna **m** apex of ovipositor, lateral view **n** claws, oblique view.

### Pseudospinaria attenuata

Taxon classificationAnimaliaHymenopteraBraconidae

(Westwood, 1882)

AC347ABD-ABAA-59E0-96F2-3816DDAE71CC

Figures 29 , 30

Spinaria attenuata Westwood, 1882: 30; Szépligeti 1902: 45.
Pseudospinaria attenuata (Westwood): Enderlein, 1905: 229.

#### Material examined.

1♀, China, Yunnan Prov., Xishuangbanna, Xiaomengyang, 850 m, 22.X.1957, Wang Shuyong, No. IOZ(E)1964528 (IZCAS); 1♀, China, Yunnan Prov., Xishuangbanna Menghai, 1100 m, 14.VIII.1957, Wang Shuyong, No. IOZ(E)1964530 (IZCAS); 1♀, China, Yunnan Prov., Xishuangbanna, Lancang, 1000 m, 30.VII.1957, Zang Lingcao, No. IOZ(E)1964529 (IZCAS); 1♀, China, Yunnan Prov., Xishuangbanna, Mengla, 620–650 m, 15.XI.1958, Zhang Yiran, No. IOZ(E)1964527 (IZCAS); 1♀, China, Hainan Prov., Shuiman, 640 m, 25.V.1960, Li Suofu, No. IOZ(E)1964637 (IZCAS).

#### Biology.

Unknown.

#### Distribution.

China (Hainan, Yunnan); Laos, Malaysia.

#### Note.

This species is newly recorded from China.

### Genus Vipiomorpha

Taxon classificationAnimaliaHymenopteraBraconidae

Tobias, 1962

3B68DCFD-51FF-5191-99A9-5488D1F4E3C0

**Figure 31.**
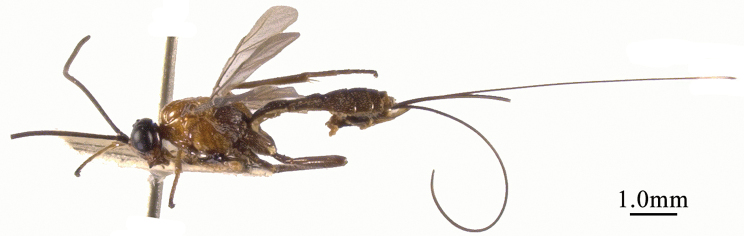
*Vipiomorphasulcata* sp. nov., ♀, holotype, habitus, lateral view.

**Figure 32.**
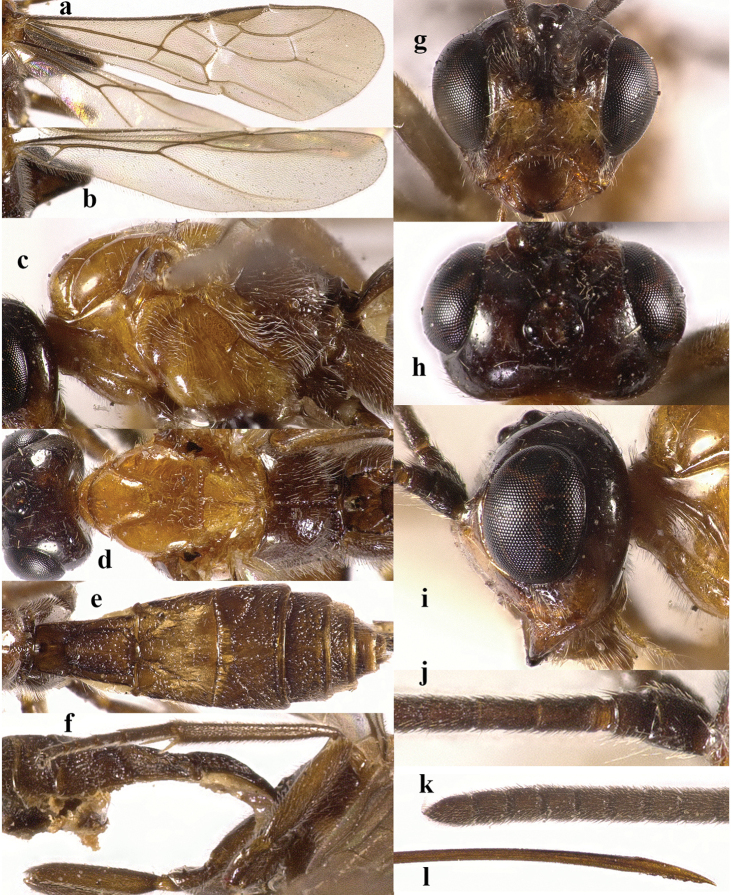
*Vipiomorphasulcata* sp. nov., ♀, holotype **a** fore wing **b** hind wing **c** mesosoma, lateral view **d** mesosoma, dorsal view **e** metasoma, dorsal view **f** hind leg, lateral view **g** head, anterior view **h** head, dorsal view **i** head, lateral view **j** scapus outer side, lateral view **k** apex of antenna **l** apex of ovipositor, lateral view.

**Figure 33.**
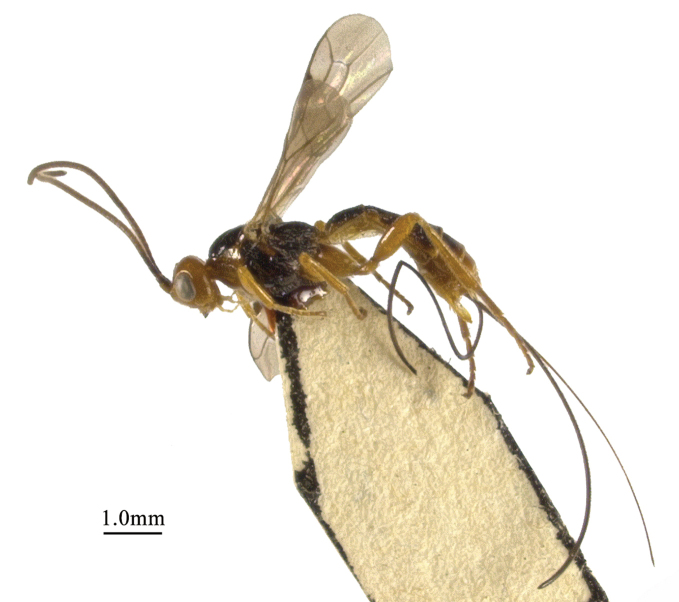
*Vipiomorphaypsilon* Tobias, 1962, ♀, habitus, lateral view.

**Figure 34.**
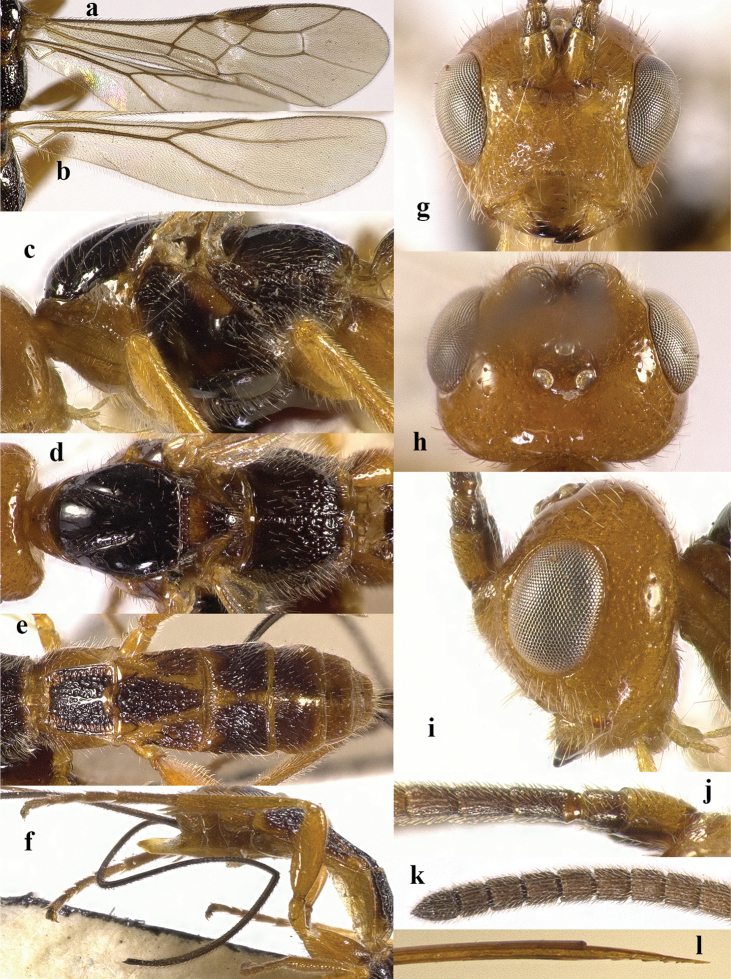
*Vipiomorphaypsilon* Tobias, 1962. ♀ **a** fore wing **b** hind wing **c** mesosoma, lateral view **d** mesosoma, dorsal view **e** metasoma, dorsal view **f** hind leg, lateral view **g** head, anterior view **h** head, dorsal view **i** head, lateral view **j** scapus outer side, lateral view **k** apex of antenna **l** apex of ovipositor, lateral view.

**Figure 35.**
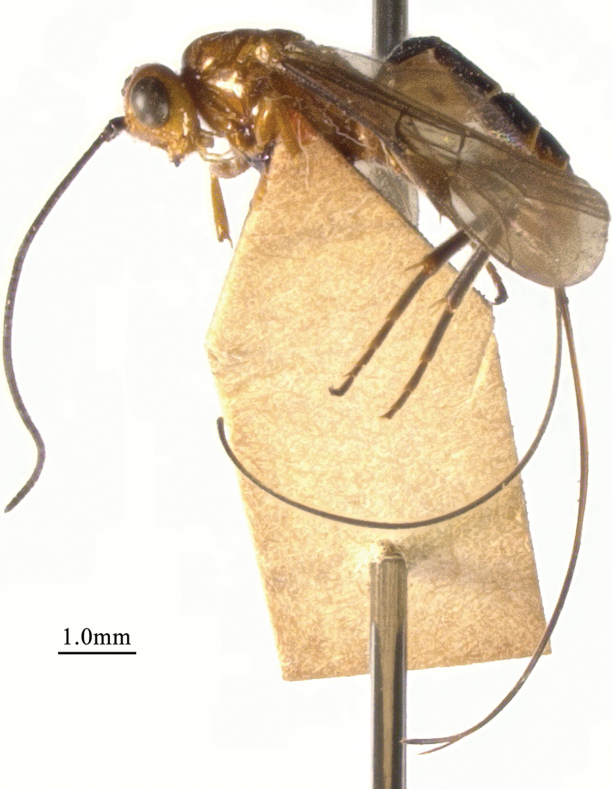
*Vipiomorphayunnanensis* sp. nov., ♀, holotype, habitus, lateral view.

**Figure 36.**
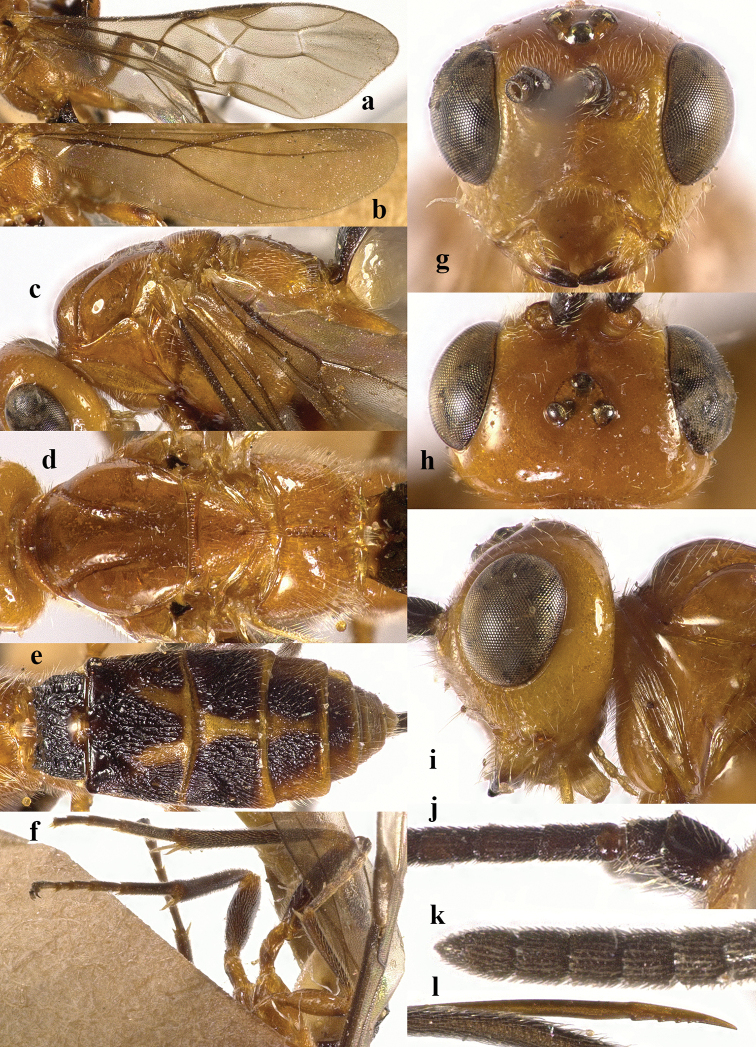
*Vipiomorphayunnanensis* sp. nov., ♀, holotype **a** fore wing **b** hind wing **c** mesosoma, lateral view **d** mesosoma, dorsal view **e** metasoma, dorsal view **f** hind leg, lateral view **g** head, anterior view **h** head, dorsal view **i** head, lateral view **j** scapus outer side, lateral view **k** apex of antenna **l** apex of ovipositor, lateral view.

Figures 31 , 32 , 33 , 34 , 35 , 36

Vipiomorpha Tobias, 1962: 1193. Type-species: Vipiomorphaypsilon Tobias, 1962 (monobasic and original designation).

#### Diagnosis.

Body small sized to medium-sized, and rather slender; terminal antennomere robust and acute apically; in lateral view scapus without double margin at inner side apically, not or slightly concave apico-laterally, ventrally weakly shorter than dorsally; eye glabrous, weakly emarginate; face largely smooth, sometimes with some sparse punctures medially; clypeus moderately narrow, dorsal clypeal carina developed (but in the Afrotropical species *Vipiomorpharugosa* (Szépligeti, 1913) absent); malar suture moderately developed, often with dense setae; labio-maxillary complex normal, not elongate; frons often strongly depressed, with a strong median groove; notauli strongly developed and complete; mesopleuron smooth, rarely with a longitudinal impression posteriorly; metapleuron smooth and shiny; metapleural flange present; propodeum punctate-rugose medially, and more pronounced postero-medially than anteriorly, with a complete medio-longitudinal groove; angle between veins 1-SR and C+SC+R of fore wing ca. 45°; marginal cell of fore wing short and elongate elliptical, vein 1-R1 shorter than pterostigma or ca. as long (at most 1.2× longer); second submarginal cell of fore wing nearly parallel-sided; fore wing veins 1-M and 1-SR+M straight; forewing vein r less than 0.5× length of m-cu; base of hind wing often with a large glabrous area; hind wing vein 1r-m distinctly oblique, and much shorter than vein SC+R1; basal lobes of claws blunt or rounded; metasoma often long and more or less slender; median area of first metasomal strongly rugose, usually with well-developed dorso-lateral carina but without medio-longitudinal carinae; lateral grooves of T I remain far removed from lateral margin of tergite; T II usually with raised smooth, shiny and large antero-lateral areas; remainder of tergite usually strongly rugose; second suture crenulate; T III usually rugose, with smooth antero-lateral areas; T III–V with or without antero-lateral areas; hypopygium extending beyond the apex of the metasomal tergites; ovipositor with dorsal nodus and ventral serrations subapically.

#### Biology.

Unknown.

#### Distribution.

Afrotropical; Oriental; Palaearctic.

#### Note.

This genus is newly recorded from the Oriental region and China; it is a small genus including three species, here we report two new species and one previously described species from China.

### Key to Chinese species of the genus *Vipiomorpha* Tobias

**Table:** 

1	Notauli relatively shallow, smooth (Fig. 32d); T I 1.5× as long as its apical width; metasomal tergites yellowish brown, without spots (Fig. 32e); head largely blackish brown dorsally (Fig. 32h), or at least (male) stemmaticum black brown [China]	***V.sulcata* sp. nov.**
–	Notauli relatively deep, at least crenulate anteriorly (Figs 34d, 36d); T I 1.0–1.2× as long as its apical width; metasomal tergites with distinct spots (Figs 34e, 36e); head yellow dorsally (Figs 34h, 36h)	**2**
2	In dorsal view length of eye 1.5× temple (Fig. 34h); mesoscutum black (Fig. 34d); hind wing vein 2-SC+R 0.7× as long as vein 1r-m (Fig. 34b); median spots of T II not touching lateral spots (Fig. 34e); T V largely smooth (Fig. 34e); scape yellowish brown, with a blackish brown streak dorsally (Fig. 34j); pterostigma blackish brown (Fig. 34a) [China; Korea; Russia]	***V.ypsilon***
–	In dorsal view length of eye 2.3× temple (Fig. 36h); mesoscutum reddish yellow (Fig. 36d); hind wing vein 2-SC+R 1.2× as long as vein 1r-m (Fig. 36b); median spots of T II touching lateral spots (Fig. 36e); T V largely rugose (Fig. 36e); scape black brown (Fig. 36j); pterostigma yellowish brown (Fig. 36a) [China]	***V.yunnanensis* sp. nov.**

### Vipiomorpha sulcata sp. nov.

Taxon classificationAnimaliaHymenopteraBraconidae

918CF7B8-9887-5499-B1F0-1DEA231DAEE2

http://zoobank.org/24D47FE0-D722-4060-B9EF-A74F12F7080B

Figures 31 , 32

#### Material examined.

***Holotype***: ♀, China, Yunnan Prov., Jinghong, 9.IV.1981, He Junhua, No. 811668 (ZJUH). ***Paratype***: 1♂, China, Yunnan Prov., Ruili, 6.V.1981, He Junhua, No. 813014 (ZJUH).

#### Diagnosis.

This new species is very similar to *Vipiomorphaypsilon* Tobias, 1962 [China; Korea; Russia], but can be separated from the latter by the following characters: notauli relatively shallow, smooth, not crenulate (deep, at least crenulate anteriorly, in *V.ypsilon*); T I 1.5× as long as apical width (1.0–1.2×); metasomal tergites yellowish brown, without spots (with distinct spots); T V largely coarsely sculptured (largely smooth); head largely blackish brown dorsally, or at least (male) stemmaticum blackish brown (head yellow dorsally).

#### Description.

Holotype, ♀, length of body 5.4 mm, of fore wing 5.3 mm, of ovipositor sheath 7.0 mm.

***Head*.** Antenna with 42 antennomeres; apical antennomere acute, 1.7× longer than its maximum width (Fig. 32k); third antennomere 1.2 and 1.3× longer than fourth and fifth, respectively, the latter 1.5× longer than wide; median antennomeres ca. 1.5× longer than their widths; malar suture with sparse short setae and with fine punctures (Fig. 32i); clypeus height: inter-tentorial distance: tentorio-ocular distance = 3: 15: 7; clypeus smooth and shiny, with a single row of sparse and long setae; eye weakly emarginate (Fig. 32g); face largely smooth, with some sparse and long setae (Fig. 32g); eye height: shortest distance between eyes: head width = 23: 22: 42; frons smooth, strongly depressed behind antennal sockets, with a strong median groove (Fig. 32h); vertex smooth, but with some sparse short setae; minimum distance between posterior ocelli: minimum diameter of elliptical posterior ocellus: minimum distance between posterior ocellus and eye = 8: 3: 10; temples largely glabrous except for a few short setae, and subparallel immediately behind eyes (Fig. 32h).

***Mesosoma*.** Length of mesosoma 1.6× its height (Fig. 32c); anterior margin of pronotum with a single row of short setae; notauli deeply impressed (Fig. 32d); mesoscutum smooth, with short and moderately dense setae (Fig. 32d); middle lobe of mesoscutum strongly convex medially; scutellar sulcus narrow and deep, with crenulae (Fig. 32d); scutellum with dense short setae posteriorly; metanotum strongly convex medially (Fig. 32d); propodeum smooth, with a complete medio-longitudinal groove, with sparse setae medially, and with dense long setae laterally (Fig. 32d).

***Wings*.** Fore wing (Fig. 32a): r very short; SR1: 3-SR: r = 8: 7: 1; 1-SR+M straight, and 1.2× longer than 1-M; 2-SR: 3-SR: r-m = 6: 11: 6; first submarginal cell of forewing short; vein SR1 ends ca. half-way between pterostigma and wing apex; cu-a interstitial. Hind wing (Fig. 32b): SC+R1: 2-SC+R: 1r-m = 8: 3: 5.

***Legs*.** Length of fore femur: tibia: tarsus = 11: 13: 16; length of hind femur: tibia: basitarsus = 39: 58: 23; length of hind basitarsus 5.75 × its maximum width (Fig. 32f).

***Metasoma*.** Length of T I 1.5× its apical width, median area convex and strongly rugose (Fig. 32e); lateral grooves of T I sparsely crenulate (Fig. 32e); median length of T II 0.7× as long as its apical width; T II strongly rugose but antero-lateral areas smooth (Fig. 32e); T II without medio-basal area; antero-lateral areas of T II developed and smooth, anterior grooves wide, with a few crenulae (Fig. 32e); second suture deep and wide, with crenulae, slightly urved medially (Fig. 32e); median length of T III 0.5× as long as its apical width; T III and T IV with antero-lateral areas (of T IV weak), and crenulate transverse subposterior groove (Fig. 32e); T V with weakly crenulate transverse subposterior groove; T III–V strongly rugose; T VI–VII largely smooth, and with some long setae posteriorly; hypopygium rather acute apically, reaching far beyond level of apex of metasoma; ovipositor sheath 1.32 × as long as fore wing.

***Colour*.** Largely yellowish brown (Fig. 31); head blackish brown except for face and mandible (but apically blackish brown) yellow (Fig. 32g, h); for legs (but claws blackish brown) yellow; ovipositor sheath black (Fig. 31); wing membrane yellow, pterostigma and veins yellowish brown (Fig. 32a, b).

***Male*.** Length of body of male 4.6 mm, of fore wing of male 4.3 mm; antenna of male with 42 antennomeres; length of forewing vein SR1: 3-SR: r = 8: 6: 1; length of T I 1.7× its apical width; T IV with noticeably antero-lateral areas; head largely yellow, area surrounded stemmaticum black brown; other characters similar with the female.

#### Biology.

Unknown.

#### Distribution.

China (Yunnan).

#### Etymology.

Named after the well-developed medio-longitudinal groove of the propodeum: *sulcus* is Latin for groove.

### Vipiomorpha ypsilon

Taxon classificationAnimaliaHymenopteraBraconidae

Tobias, 1962

B39139AA-6AF8-56B4-8169-8A0924427468

Figures 33 , 34

Vipiomorpha ypsilon Tobias, 1962: 1194.

#### Material examined.

14♀♀17♂♂, China, Liaoning Prov., Dalian, 16.VII.1994, Lou Juxian, No. 975837, 952150, 952217, 952170, 952171, 952204, 952169, 952147, 952372, 952359, 952317, 952323, 952329, 952339, 952288, 952206, 952331, 952179, 952206, 952194, 952203, 952335, 952191, 952157, 952360, 952215, 952196, 952276, 952298, 952374 (ZJUH); 1♀1♂, China, Liaoning Prov., Shenyang, Dongling, ?.V–VI.1994, Lou Juxian, No. 947490, 947491 (ZJUH); 6♀♀11♂♂, China, Liaoning Prov., Dalian, 5.IX.1992, Lou Juxian, No. 976211, 976217, 976209, 976014, 976157, 976093, 976154, 976258, 976208, 976248, 975977, 976109, 976232, 976171, 976225, 976200, 976085 (ZJUH); 1♂, China, Liaoning Prov., Shenyang, Dongling, 21.VI.1994, Lou Juxian, No. 947748 (ZJUH); 3♀♀5♂♂, China, Liaoning Prov., Fuxin, 16–23.VIII.1995, Lou Juxian, No. 961180, 961259, 961194, 961182, 961304, 961268, 961391, 961284 (ZJUH); 1♀5♂♂, China, Liaoning Prov., Fuxin, 25.VIII.1994, Lou Juxian, No. 951349, 951389, 951345, 951347, 951398, 951386 (ZJUH); 1♂, China, Jilin Prov., Changchun, 23.VII.1992, Lou Juxian, No. 951051 (ZJUH); 1♂, China, Jilin Prov., Liaoyuan, 21.VII.1991, Lou Juxian, No. 950411 (ZJUH); 1♂, China, Jilin Prov., Liaoyuan, 10.VIII.1990, Lou Juxian, No. 977137 (ZJUH).

#### Biology.

Unknown.

#### Distribution.

China (Jilin, Liaoning); Korea; Russia.

#### Note.

This species is new to China.

### Vipiomorpha yunnanensis sp. nov.

Taxon classificationAnimaliaHymenopteraBraconidae

7B2FB679-9AEF-504D-A423-D7D4D93EB3B1

http://zoobank.org/BF499D14-CA6F-4F54-98EC-ED79AF97EB60

Figures 35 , 36

#### Material examined.

***Holotype***: ♀, China, Yunnan Prov., Jingkan, 18.V.1983, Liao Yichang, No. 841267 (ZJUH).

#### Diagnosis.

This new species is very similar to *Vipiomorphaypsilon* Tobias, 1962 [China; Korea; Russia], but can be separated from the latter by the following characters: in dorsal view length of eye 2.3× temple (1.5× in *V.ypsilon*); mesoscutum reddish yellow (black); hind wing vein 2-SC+R 1.2× as long as vein 1r-m (0.7×); median spots of T II touching lateral spots anteriorly (not touching lateral spots); T V largely rugose (largely smooth); scape blackish brown (yellowish brown, with a black brown streak dorsally); pterostigma yellowish brown (blackish brown).

#### Description.

Holotype, ♀, length of body 6.6 mm, of fore wing 6.0 mm, of ovipositor sheath 8.0 mm.

***Head*.** Antenna with 50 antennomeres; apical antennomere acute, 1.5× longer than its maximum width (Fig. 36k); third antennomere 1.2 and 1.3× longer than fourth and fifth, respectively, the latter 1.6× longer than wide; median antennomeres ca. 1.1× longer than their width; malar suture with dense short setae (Fig. 36i); clypeus height: inter-tentorial distance: tentorio-ocular distance = 4: 11: 7; clypeus smooth and shiny, with a single row of long setae; eye weakly emarginate(Fig. 36g); face largely smooth, with sparse and long setae (Fig. 36g); eye height: shortest distance between eyes: head width = 11: 14: 26; frons largely smooth except for a few weak punctures, strongly depressed behind antennal sockets, with a strong median groove (Fig. 36h); vertex smooth, but with some sparse short setae; minimum distance between posterior ocelli: minimum diameter of elliptical posterior ocellus: minimum distance between posterior ocellus and eye = 4: 3: 8; temples largely smooth except for a few weak punctures, and with some short setae, slightly narrowed immediately behind eyes (Fig. 36h).

***Mesosoma*.** Length of mesosoma 1.5× its height (Fig. 36c); anterior margin of pronotum with a single row of short setae; notauli deeply impressed, with a few weak crenulae (Fig. 36d); mesoscutum smooth, with short and dense setae posteriorly (Fig. 36d); middle lobe of mesoscutum strongly convex medially; scutellar sulcus moderately wide and deep, with crenulae (Fig. 36d); scutellum with a few weak punctures, and with dense short setae posteriorly; metanotum strongly convex medially (Fig. 36d); propodeum largely smooth, with a complete and crenulate medio-longitudinal groove, with sparse setae medially, and with dense long setae laterally (Fig. 36d).

***Wings*.** Fore wing (Fig. 36a): r very short; r: SR1: 3-SR: = 19: 19: 3; 1-SR+M straight, and 1.8× longer than 1-M; 2-SR: 3-SR: r-m = 8: 19: 9; first submarginal cell of forewing short; vein SR1 ends basally of mid-point between pterostigma and wing tip (at ca. 0.44); cu-a interstitial. Hind wing (Fig. 36b): r-m weakly curved; SC+R1: 2-SC+R: 1r-m = 9: 6: 5.

***Legs*.** Length of fore femur: tibia: tarsus = 20: 23: 30; length of hind femur: tibia: basitarsus = 31: 34: 18; length of hind basitarsus 5.7× its maximum width (Fig. 36f).

***Metasoma*.** Length of T I 1.1× its apical width, median area convex and strongly rugose (Fig. 36e); lateral grooves of T I strongly crenulate (Fig. 36e); median length of T II 0.7× as long as its apical width; T II strongly rugose but antero-lateral areas smooth (Fig. 36e); T II without medio-basal area; antero-lateral areas of T II developed and smooth, anterior grooves wide, with a few weak crenulae (Fig. 36e); second suture deep and wide, with crenulae, slightly curved medially (Fig. 36e); median length of T III 0.37 × as long as its apical width; T III–IV with antero-lateral areas (of T IV weak), and crenulate transverse subposterior groove (Fig. 36e); T V with weak crenulate transverse subposterior groove; T III–VI rugose except posteriorly (T V and T VI weakly so); T VII largely smooth, and with some long setae posteriorly; hypopygium rather acute apically, far beyond level of apex of metasoma; ovipositor sheath 1.33 × as long as fore wing.

***Colour*.** Largely yellow (Fig. 35); antenna, eye, mandible apically, claws, middle tibia and tarsus, hind femur, tibia and tarsus and ovipositor sheath black (Figs 35, 36g, f); T I black, T II–IV largely black (except posteriorly and T IV laterally), and with a yellow Y-shaped spot, T V black medio-anteriorly (Fig. 36e); wing membrane infuscate, pterostigma and veins yellowish brown (Fig. 36a, b).

#### Biology.

Unknown.

#### Distribution.

China (Yunnan).

#### Etymology.

Named after its type locality, the southwestern province of Yunnan.

### Genus Zaglyptogastra

Taxon classificationAnimaliaHymenopteraBraconidae

Ashmead, 1900

76D57059-AD47-5C86-9090-A26915234B6C

**Figure 37.**
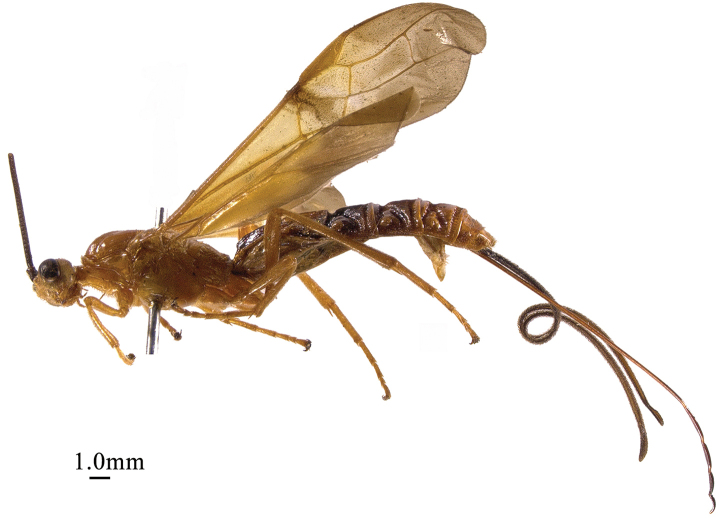
*Zaglyptogastraexilis* sp. nov., ♀, holotype, habitus, lateral view.

**Figure 38.**
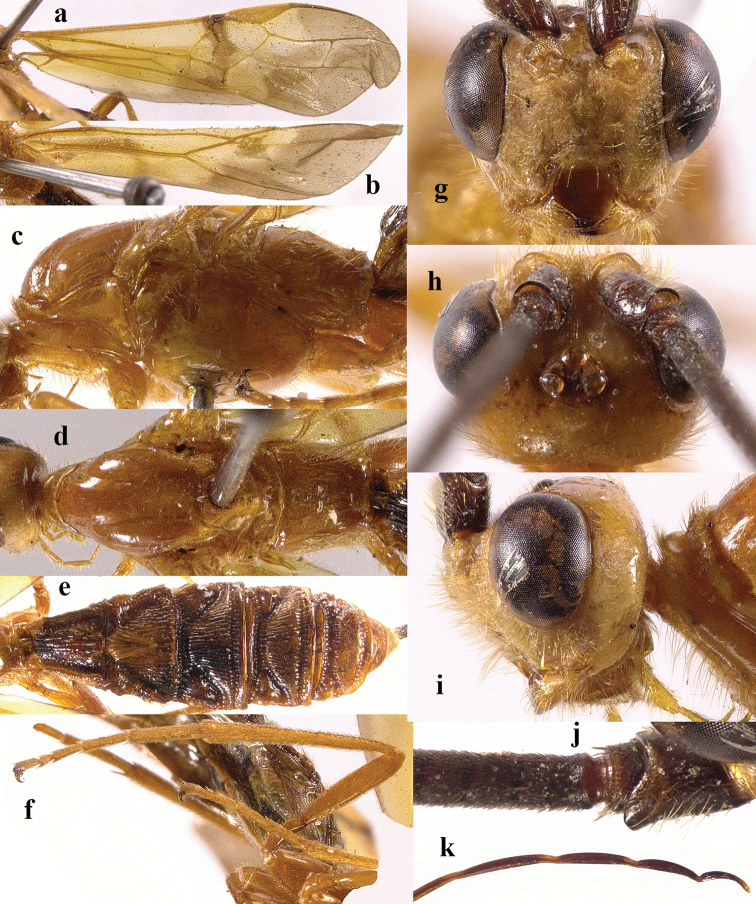
*Zaglyptogastraexilis* sp. nov., ♀, holotype **a** fore wing **b** hind wing **c** mesosoma, lateral view **d** mesosoma, dorsal view **e** metasoma, dorsal view **f** hind leg, lateral view **g** head, anterior view **h** head, dorsal view **i** head, lateral view **j** scapus outer side, lateral view **k** apex of ovipositor, lateral view.

**Figure 39.**
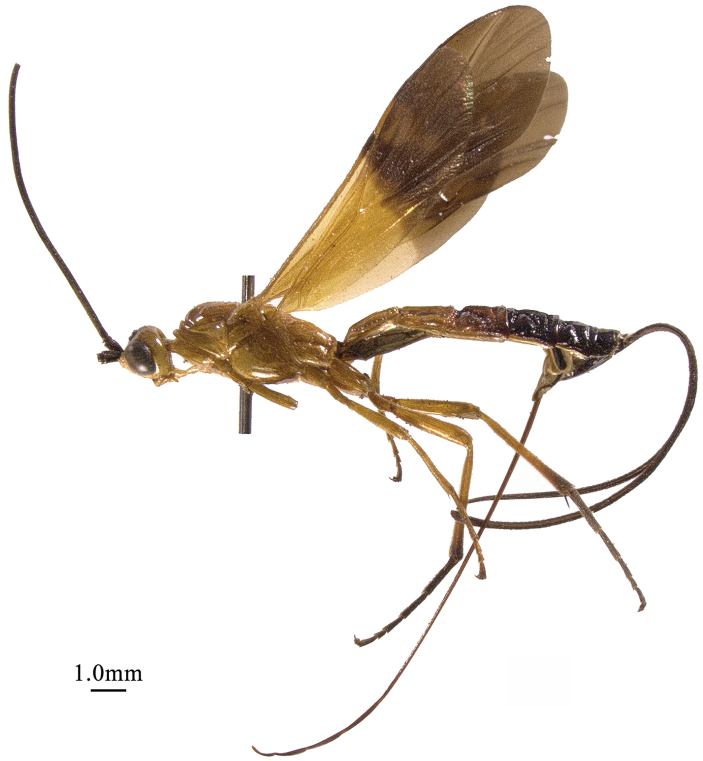
*Zaglyptogastratricolor* sp. nov., ♀, holotype, habitus, lateral view.

**Figure 40.**
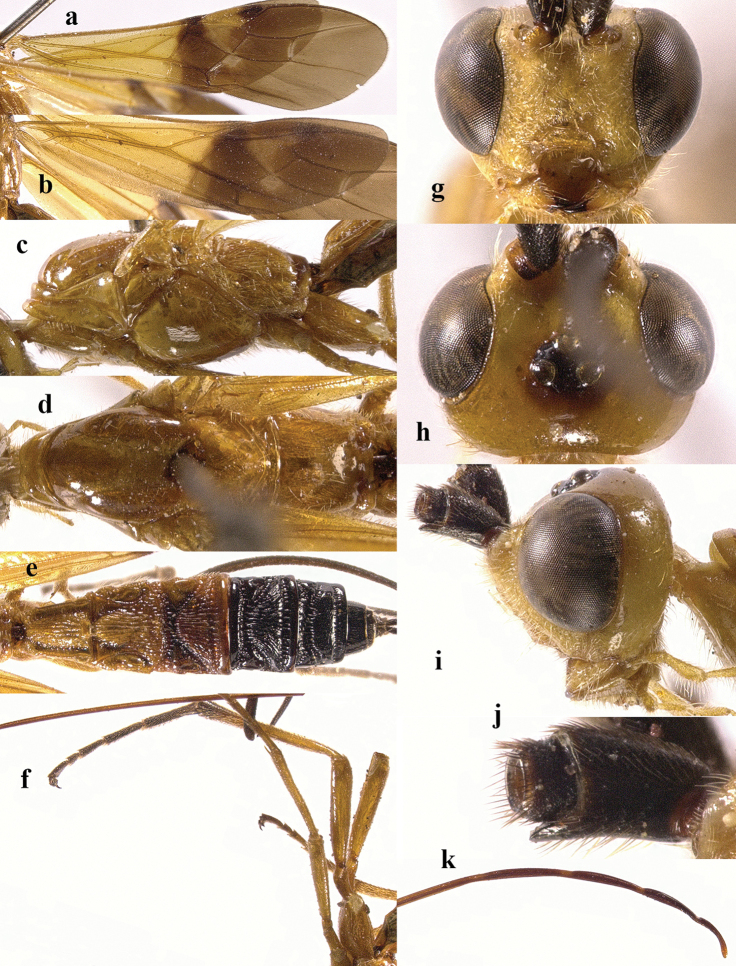
*Zaglyptogastratricolor* sp. nov., ♀, holotype **a** fore wing **b** hind wing **c** mesosoma, lateral view **d** mesosoma, dorsal view **e** metasoma, dorsal view **f** hind leg, lateral view **g** head, anterior view **h** head, dorsal view **i** head, lateral view **j** scapus outer side, lateral view **k** apex of ovipositor, lateral view.

Figures 37 , 38 , 39 , 40

Zaglyptogastra Ashmead, 1900: 137; Quicke 1981: 494, 1987: 136; El-Heneidy and Quicke 1991: 185. Type-species: Zaglyptogastraabbotti Ashmead, 1900 (Monobasic and original designation).
Eumorpha Szépligeti, 1908: 35. Type-species: Eumorphanigripennis Szépligeti, 1908 (Monobasic). Synonymised by El-Heneidy and Quicke 1991: 185.
Holcobracon Cameron, 1909: 19 (not Cameron 1905). Type-species: Holcobraconerythraspis Cameron, 1909 (Monobasic). Synonymised by Quicke 1981: 493.
Calliidia Schulz, 1911: 68 (not Hubner 1806, Frieze 1899). Replacement name for Eumorpha Szépligeti, 1908.
Megagonia Szépligeti, 1906: 582. Type-species: Megagoniaseminigra Szépligeti, 1906 (Designated by Viereck 1914: 90). Synonymised by Quicke 1981: 493.
Holconotus Fahringer, 1928: 19 (not Schmidt-Goebel 1846, Agassiz 1854 or Foerster 1863). Replacement name for Holcobracon Cameron, 1909.
Holcosomius Fahringer, 1935: 634. Replacement name for Holconotus Fahringer, 1928.

#### Diagnosis.

Body medium-sized to large; terminal antennomeres sometimes acute apically; median antennomeres usually wider than long; in lateral view scapus without double margin at inner side apically and concave apico-laterally, ventrally longer than dorsally; eye glabrous, large (smaller in male) and weakly emarginate; face often sculptured; clypeus moderately narrow, often flattened and without dorsal carina; malar suture moderately developed, often rugose; labio-maxillary complex normal, not elongate; frons depressed, with a weak to moderately developed median groove; mesosoma largely smooth and shiny; notauli weak, often only present anteriorly; scutellar sulcus narrow and usually crenulate; mesopleural suture smooth; precoxal suture absent; metanotum convex medially, and with a short median carina, somewhat protruding; propodeum often smooth, without medio-longitudinal carina or groove; angle between veins 1-SR and C+SC+R of fore wing more than 75°; marginal cell moderated long, vein SR1 reaching the wing margin at least 0.8× of distance between pterostigma and wing tip; second submarginal cell of fore wing usually more or less parallel-sided; fore wing vein r-m usually with both a distinct anterior and posterior bulla; fore wing vein 1-SR+M usually strongly bent after arising from 1-M, sometimes more or less evenly curved to straight; fore wing vein cu-a interstitial to distinctly postfurcal; fore wing vein 3-CU1 usually distinctly thickened posteriorly; hind wing vein 1r-m usually longer or at least as long as vein SC+R1, rarely shorter; apex of hind wing vein C+SC+R with more than one especially thickened bristle; claws simple; metasomal tergites usually largely strongly sculptured, rarely smooth; T I without medio-longitudinal carina, dorso-lateral carinae present or absent; T II usually with a weakly to strongly raised medio-basal triangular area which usually has similar sculpture as remainder of T; T II–IV often with strong antero-lateral areas and grooves; T III–V often with strongly crenulate transverse subposterior grooves, rarely absent; hypopygium rather acute apically; ovipositor usually as long as or longer than fore wing, with some (three or four) depressions and upper valve enlarged, subapically without dorsal nodus and ventral serrations (rarely distinct).

#### Biology.

Quicke (1983) reported only one species, *Tryphochariaprinceps* (Blackburn) (Coleoptera: Cerambycidae) as a host of *Z.cristata* (Szépligeti).

#### Distribution.

Afrotropical; Australasian; Oriental; Palaearctic.

#### Note.

This genus is newly recorded from the Oriental region.

### Key to Chinese species of the genus *Zaglyptogastra* Ashmead

**Table:** 

1	Metasomal tergites yellowish brown (Fig. 38e); fore wing vein SR1 relatively short, 1.1× as long as vein 3-SR (Fig. 38a); triangular medio-basal area of T II rather large, nearly reaching posterior margin of tergite (Fig. 38e); pterostigma largely yellow, blackish brown apically (Fig. 38a); hind leg largely yellow, only tarsus blackish brown apically (Fig. 38f); stemmaticum yellow (Fig. 38h); ovipositor sheath with yellowish setae (Fig. 37) [China]	***Z.exilis* sp. nov.**
–	T I–II yellow, T III yellowish brown, T IV–VII black (Fig. 40e); fore wing vein SR1 relatively long, 1.6× as long as vein 3-SR (Fig. 40a); T II without triangular medio-basal area (Fig. 40e); apical 2/5 of pterostigma blackish brown, and basal 3/5 yellow (Fig. 40a); hind tibia apically and tarsus blackish brown (Fig. 40f); stemmaticum blackish brown (Fig. 40h); ovipositor sheath with blackish setae (Fig. 39) [China]	***Z.tricolor* sp. nov.**

### Zaglyptogastra exilis sp. nov.

Taxon classificationAnimaliaHymenopteraBraconidae

1FB49FBF-73BB-5E05-9679-5679679ECDE4

http://zoobank.org/824F1AF8-6127-4BCE-A387-894E9C488B46

Figures 37 , 38

#### Material examined.

***Holotype***: ♀, China, Hainan Prov., Jianfengling, 1.IV.1984, Gu Maobin, No. IOZ(E)1964587 (IZCAS).

#### Diagnosis.

This new species is very similar to *Zaglyptogastraabbotti* Ashmead, 1900 [Indonesia; Malaysia; Thailand], but can be separated from the latter by the following characters: scape blackish brown (brown or yellowish brown dorsally in *Z.abbotti*); wing membrane yellow, greyish brown apically, stigmal spot rather large, enclosing entire vein CU1b (greyish brown area of membrane narrower, and stigmal spot small); T V with striae medio-basally (smooth, only with few punctures, without striae).

#### Description.

Holotype, ♀, length of body 18.6 mm, of fore wing 17.2 mm, of ovipositor sheath 17.0 mm.

***Head*.** Antenna incomplete, with 63 antennomeres remaining; third antennomere 1.4 and 1.6× longer than fourth and fifth, respectively, the latter 0.8× longer than wide; malar suture with dense short setae (Fig. 38i); inter-tentorial distance: tentorio-ocular distance = 7: 5; clypeus largely smooth; eye rather emarginated (Fig. 38g); face coarsely rugose, with some long setae (Fig. 38g); shortest distance between eyes: head width = 24: 42; frons smooth, moderately depressed behind antennal sockets, with a median groove (Fig. 38h); vertex smooth, but with some short setae; minimum distance between posterior ocelli: minimum diameter of elliptical posterior ocellus: minimum distance between posterior ocellus and eye = 5: 7: 12; temples largely smooth except for a few weak punctures, and with dense long setae laterally, subparallel immediately behind eyes (Fig. 38h).

***Mesosoma*.** Length of mesosoma 2.0× its height (Fig. 38c); notauli impressed in anterior half of mesoscutum (Fig. 38d); scutellar sulcus moderately wide and deep, with crenulae (Fig. 38d); scutellum with some short setae posteriorly; propodeum smooth, with sparse setae medially, and with dense long setae laterally (Fig. 38d).

***Wings*.** Fore wing (Fig. 38a): pterostigma 3.5× longer than wide; SR1: 3-SR: r = 39: 35: 7; 1-SR+M slightly curved subbasally, and 1.4× longer than 1-M; 2-SR: 3-SR: r-m = 14: 35: 15; 3-CU1 distinctly thickened posteriorly; cu-a slightly postfurcal. Hind wing (Fig. 38b): 1r-m 1.2× longer than SC+R1; apex of C+SC+R with nine thickened basal bristles; base with a medium-sized glabrous area distal to vein cu-a.

***Legs*.** Length of fore femur: tibia: tarsus = 34: 39: 49; length of fore basitarsus 4.7× its maximally width; length of hind femur: tibia: basitarsus = 37: 64: 23 (Fig. 38f); length of hind basitarsus 6.3× its maximum width.

***Metasoma*.** Length of T I 1.3× its apical width, median area convex and longitudinally rugose (Fig. 38e); lateral grooves of T I strongly crenulate (Fig. 38e); apical width of T II 1.8× as long as its median length; T II strongly rugose but antero-lateral areas smooth (Fig. 38e); T II with large medio-basal area which ends near posterior margin of T II; antero-lateral areas of T II developed and smooth, anterior grooves wide, with a few crenulae (Fig. 38e); second suture deep and wide, with crenulae, more or less straight medially (Fig. 38e); T III–V with strong antero-lateral areas and grooves, and with strong crenulate transverse subposterior grooves; T III and T IV strongly rugose but antero-lateral areas smooth (Fig. 38e); T V weakly rugose antero-medially, smooth laterally and posteriorly; T VI and T VII largely smooth; ovipositor sheath as long as fore wing.

***Colour*.** Head and mesosoma largely yellow (Fig. 37); antenna, eye and mandible apically black (Fig. 38g); legs largely yellow, tarsi apically and claws black (Fig. 38f); ovipositor sheath black, with yellow setae (Fig. 37); T I–IV blackish brown (T II yellowish brown medio-basally), T V–VII yellowish brown (Fig. 38e); wing membrane 2/3 basally yellow and 1/3 apically brownish (in hind wing up to posterior margin); stigmal spot yellowish brown and reaching vein 2-1A; pterostigma yellow except for extreme apex; veins largely yellow, fore wing veins 1-SR, 3-CU1, apical half of 3-SR and 2-M, SR1, 3-M, hind wing veins SR apically, 2-M medially and apically yellowish brown (Fig. 38a, b).

#### Biology.

Unknown.

#### Distribution.

China (Hainan).

#### Etymology.

Named after the rather slender body: *exilis* is Latin for slender.

### Zaglyptogastra tricolor sp. nov.

Taxon classificationAnimaliaHymenopteraBraconidae

199D5A31-A8B6-53B8-9F77-9CEA7157578E

http://zoobank.org/9C6F7ED7-BA6E-4525-B66D-0F4BE8DC451F

Figures 39 , 40

#### Material examined.

***Holotype***: ♀, China, Yunnan Prov., near Jingdong, 1300 m, 17.III.1957, Bangfeiluofu, No. IOZ(E)1964532 (IZCAS).

#### Diagnosis.

This new species is very similar to *Zaglyptogastraplumiseta* (Enderlein, 1920) [Indonesia; Malaysia; Singapore], but can be separated from the latter by the following characters: fore wing vein SR1 relatively long, 1.6× as long as vein 3-SR (at most 1.1× in *Z.plumiseta*); hind leg yellow, tibia apically and tarsus black brown (entirely black); T I–II yellow, T III yellowish brown, T IV–VII black (uniformly black).

#### Description.

Holotype, ♀, length of body 13.2 mm, of fore wing 11.3 mm, of ovipositor sheath 12.8 mm.

***Head*.** Antenna incomplete, 55 antennomeres remaining; third antennomere 1.3 and 1.4× longer than fourth and fifth, respectively, the latter as long as wide; malar suture with dense short setae (Fig. 40i); inter-tentorial distance: tentorio-ocular distance = 12: 7; clypeus largely smooth; eye rather emarginate (Fig. 40g); face coarsely sculptured, with some sparse and long setae (Fig. 40g); shortest distance between eyes: head width = 18: 37; frons smooth, moderately depressed behind antennal sockets, with a median groove (Fig. 40h); vertex smooth, but with some sparse short setae; minimum distance between posterior ocelli: minimum diameter of elliptical posterior ocellus: minimum distance between posterior ocellus and eye = 5: 7: 12; temples largely smooth except for a few weak punctures, and with dense long setae laterally, weakly narrowed immediately behind eyes (Fig. 40h).

***Mesosoma*.** Length of mesosoma 1.9× its height (Fig. 40c); notauli impressed in anterior half of mesoscutum (Fig. 40d); scutellar sulcus moderately wide and deep, with crenulae (Fig. 40d); scutellum with some short setae posteriorly; propodeum smooth, with sparse setae medially, and with dense long setae laterally (Fig. 40d).

***Wings*.** Fore wing (Fig. 40a): pterostigma 3.5× longer than wide; SR1: 3-SR: r = 39: 24: 5; 1-SR+M more or less straight, and 1.5× longer than 1-M; 2-SR: 3-SR: r-m = 13: 24: 11; 3-CU1 distinctly thickened posteriorly; cu-a interstitial. Hind wing (Fig. 40b): 1r-m 1.2× longer than SC+R1; apex of C+SC+R with six thickened basal bristles; base with a small glabrous area distal to vein cu-a.

***Legs*.** Length of fore femur: tibia: tarsus = 42: 49: 64; length of fore basitarsus 5.6× its maximally width; length of hind femur: tibia: basitarsus = 40: 64: 23 (Fig. 40f); length of hind basitarsus 6.6× its maximum width.

***Metasoma*.** Length of T I 1.4× its apical width, median area convex and longitudinally rugose (Fig. 40e); lateral grooves of T I strongly crenulate (Fig. 40e); apical width of T II 1.4× as long as its median length; T II strongly longitudinally rugose but antero-lateral areas smooth (Fig. 40e); T II with large but weakly raised medio-basal area nearly reaching antero-lateral areas; antero-lateral areas of T II narrow and smooth, anterior grooves narrow, with a few sparse crenulae (Fig. 40e); second suture deep and wide, with crenulae, straight medially (Fig. 40e); T III–V with strong antero-lateral areas and grooves, and with strong crenulate transverse subposterior grooves; T III and T IV strongly longitudinally rugose but antero-lateral areas smooth (Fig. 40e); T V weakly rugose antero-medially, smooth laterally and posteriorly; T VI and T VII largely smooth; ovipositor sheath 1.1× longer than fore wing.

***Colour*.** Head and mesosoma largely yellow (Fig. 39); antenna, eye, surroundings of stemmaticum, stemmaticum and mandible apically black (Fig. 40g, h); legs largely yellow, fore and middle tarsi apically, hind tibia apically, hind tarsus, and claws black (Fig. 40f); ovipositor sheath black, with blackish brown setae (Fig. 39); T I and T II yellow, T III yellowish brown, T IV–VII black (but posterior margins of T VI and T VII yellow; Fig. 40e); basal half of wing membrane yellow and apical half blackish brown; stigmal spot blackish brown and reaching vein 2-1A, surroundings of vein 2-SR+M and besides vein r-m subhyaline; basal 3/5 of pterostigma yellow (narrowly brown basally) and apically 2/5 blackish brown; fore wing veins M+CU1, 1-1A, cu-a, 1-M apically, 2-CU1 basally and 2-1A basally yellow, remaining fore wing veins blackish brown, hind wing veins SC+R1 anteriorly, SR (except for external basally blackish brown), 2-M medially and apically blackish brown, remaining hind wing veins yellow (Fig. 40a, b).

#### Biology.

Unknown.

#### Distribution.

China (Yunnan).

#### Etymology.

Named after the tri-coloured metasoma: *tricolor* is Latin for “with three colours”.

## Supplementary Material

XML Treatment for Chaoilta

XML Treatment for Chaoilta breviceps

XML Treatment for Chaoilta himalayensis

XML Treatment for Cyanopterus

XML Treatment for Cyanopterus (Bracomorpha) lucidus

XML Treatment for Cyanopterus (Bracomorpha) ninghais

XML Treatment for Cyanopterus (Bracomorpha) transversus

XML Treatment for Cyanopterus (Bracomorpha) tricolor

XML Treatment for Cyanopterus (Paravipio) jakuticus

XML Treatment for Gammabracon

XML Treatment for Gammabracon uniformis

XML Treatment for Gammabracon wangi

XML Treatment for Ischnobracon

XML Treatment for Ischnobracon guttatus

XML Treatment for Ischnobracon hannongbuai

XML Treatment for Ischnobracon v-macula

XML Treatment for Monilobracon

XML Treatment for Monilobracon longitudinalis

XML Treatment for Monilobracon marginatus

XML Treatment for Parallobracon

XML Treatment for Parallobracon prolatus

XML Treatment for Pseudospinaria

XML Treatment for Pseudospinaria attenuata

XML Treatment for Vipiomorpha

XML Treatment for Vipiomorpha sulcata

XML Treatment for Vipiomorpha ypsilon

XML Treatment for Vipiomorpha yunnanensis

XML Treatment for Zaglyptogastra

XML Treatment for Zaglyptogastra exilis

XML Treatment for Zaglyptogastra tricolor
